# Design, Synthesis, and Antiproliferative Activity of Benzopyran-4-One-Isoxazole Hybrid Compounds

**DOI:** 10.3390/molecules28104220

**Published:** 2023-05-21

**Authors:** Shilpi Gupta, Shang Eun Park, Saghar Mozaffari, Bishoy El-Aarag, Keykavous Parang, Rakesh Kumar Tiwari

**Affiliations:** 1 Center for Targeted Drug Delivery, Department of Biomedical and Pharmaceutical Sciences, Chapman University School of Pharmacy, Harry and Diane Rinker Health Science Campus, Irvine, CA 92618-1908, USA; drshilpigupta.chem@gmail.com (S.G.); mozaf100@mail.chapman.edu (S.M.); bishoy.yousef@gmail.com (B.E.-A.);; 2 Department of Chemistry, Hindu College, Sonipat 131001, India; 3 Biochemistry Division, Chemistry Department, Faculty of Science, Menoufia University, Shebin El-Koom 32512, Egypt

**Keywords:** antiproliferative, apoptosis, benzopyranone, chromone, drug discovery, isoxazole, protein tyrosine kinase

## Abstract

The biological significance of benzopyran-4-ones as cytotoxic agents against multi-drug resistant cancer cell lines and isoxazoles as anti-inflammatory agents in cellular assays prompted us to design and synthesize their hybrid compounds and explore their antiproliferative activity against a panel of six cancer cell lines and two normal cell lines. Compounds **5a**–**d** displayed significant antiproliferative activities against all the cancer cell lines tested, and IC_50_ values were in the range of 5.2–22.2 μM against MDA-MB-231 cancer cells, while they were minimally cytotoxic to the HEK-293 and LLC-PK1 normal cell lines. The IC_50_ values of **5a**–**d** against normal HEK-293 cells were in the range of 102.4–293.2 μM. Compound **5a** was screened for kinase inhibitory activity, proteolytic human serum stability, and apoptotic activity. The compound was found inactive towards different kinases, while it completely degraded after 2 h of incubation with human serum. At 5 μM concentration, it induced apoptosis in MDA-MB-231 by 50.8%. Overall, these findings suggest that new benzopyran-4-one-isoxazole hybrid compounds, particularly **5a**–**d**, are selective anticancer agents, potentially safe for human cells, and could be synthesized at low cost. Additionally, Compound **5a** exhibits potential anticancer activity mediated via inhibition of cancer cell proliferation and induction of apoptosis.

## 1. Introduction

The primary factors influencing cancer treatment are the patient’s cancer type and stage. There are several methods of treatment, including surgery, chemotherapy, radiation, hormone therapy, immunotherapy, and targeted therapy [1,2]. Eliminating cancer cells, limiting their growth, and spread, and ultimately curing the illness or lengthening the patient’s life are the goals of therapy [3]. Despite significant progress in cancer therapy, chemotherapy, which has many inherent drawbacks, remains a primary choice for treatment. To overcome the shortcomings of chemotherapy, including drug resistance, side effects, low effectiveness, lack of personalization, and high drug costs, novel compounds are still needed to enhance efficacy, safety, and personalization while being affordable [4,5,6]. In terms of effectiveness and safety, targeted therapeutic “small molecule” drugs have exceeded traditional chemotherapeutic drugs, and are now often employed to treat cancer [4].

Benzopyran-4-ones (also known as chromones) have been referred to as a “Privileged Scaffold in Drug Discovery” and have been extensively reviewed in the last few years [7,8,9,10,11]. The benzopyran-4-one skeleton has a variety of pharmacological properties, such as anticancer, antiallergic, antitubercular, anti-inflammatory, antidiabetic, antimicrobial, antihypertensive, and anti-HIV, and has been invariably used as a template for the design of novel compounds with diverse therapeutic profiles [7,8,9,10,11]. 3-Substituted benzopyran-4-one derivatives have been identified as potential anticancer agents from biological screening on cancer cell lines. A few noteworthy examples include those bearing imidazolidinone methylamino-4-substituted-1,3-thiazoles and 4-morpholinothieno [3,2-d]pyrimidine substituents [12,13,14,15]. Furthermore, several chromonylthiazolidines were found to have selective cytotoxicity against human epidermoid carcinoma and breast cancer [16]. Additionally, 7-methoxy benzopyran-4-one derivatives were reported to be cytotoxic in several cell lines (HL-60, KB, LLC, LNCaP, LU-1, MCF7, and SW480) [17]. Another class of benzopyran-4-ones, i.e., bis-benzopyran-4-one derivatives, have also shown notable in vitro antiproliferative activity against various human cancer cell lines such as PC-3, NCI-H23, MDA-MB-231, HCT-15, NUGC-3, and ACHN cancer cell lines [18].

Another important heterocyclic skeleton of current interest is isoxazole, which along with some naturally occurring isoxazole derivatives, has interesting biological activities [19,20,21,22]. For example, Ibotenic acid ((S)-2-amino-2-(3-hydroxyisoxazol-5-yl)acetic acid), isolated from the mushroom *Amanita muscaria* [23], is a potent neurotoxin similar to kainic acid and muscimol. The compound is a potent and selective agonist for the γ-aminobutyric acid A (GABA_A_) receptors. Previous reports demonstrated the high neurotoxicity of Ibotenic acid when injected directly into the brains of mice [23,24]. It can cross the blood–brain barrier, may remain unchanged, or may get metabolized into muscimol [25,26]. Isoxazole is also an essential pharmacophore for many drugs, such as leflunomide (antirheumatic drug, DMARD), valdecoxib (COX-2 inhibitor), and zonisamide (anticonvulsant). Isoxazole derivatives have also been explored as potential therapeutic agents against colon cancer [27] and anti-inflammatory agents for in vivo studies [28,29]. Dimethyl isoxazole derivatives have also been identified as a new class of BET bromodomain inhibitors and have shown anti-inflammatory properties in cellular assays [30,31,32,33].

**Figure 1:**
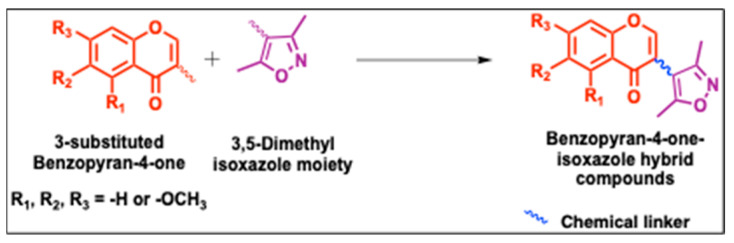
Design of benzopyran-4-one-isoxazole hybrid compounds.

Considering the intriguing biological activities of 3-substituted benzopyran-4-one and isoxazole scaffolds in drug discovery and medicinal chemistry, their fusion into the same framework leading to a hybrid molecule (Figure 1) is of particular interest to instigate a chemical entity that is pharmaceutically more active than the individual constituents. Both individual constituents, i.e., benzopyran-4-ones and isoxazoles, have been studied extensively, but their fusion with different chemical linkers to generate new hybrid compounds has not been studied so far. It was anticipated that their fusion might develop compounds with better biological activity, and thus, it became an area of our interest. Therefore, we hypothesize a new series of compounds where isoxazoles are conjugated to benzopyran-4-one at position 3 with five different chemical linkers, i.e., ester (formed from 3-(hydroxymethyl) substituted-4-oxo-4*H*-1-benzopyran), acetal, amide, reverse ester (formed from (3,5-dimethyl-isoxazol-4-yl)-methanol), and acrylic ester to possess potential pharmacological profiles (Figure 1). As a preliminary study, different linkers were designed to conjugate the isoxazole scaffold to the unsubstituted benzopyran-4-one nucleus; then, a library of compounds was synthesized by substituting the benzene ring of benzopyran-4-one at positions 5-, 6- and 7- with methoxy substituent to study the structure-activity relationship (SAR) and screened them towards their antiproliferative activity against a panel of different cancer cell lines. They were also screened for their kinase inhibitory activity, proteolytic human serum stability, and apoptotic activity to demonstrate their potential as antiproliferative agents.

## 2. Results and Discussion

### 2.1. Chemistry

**Scheme 1:**
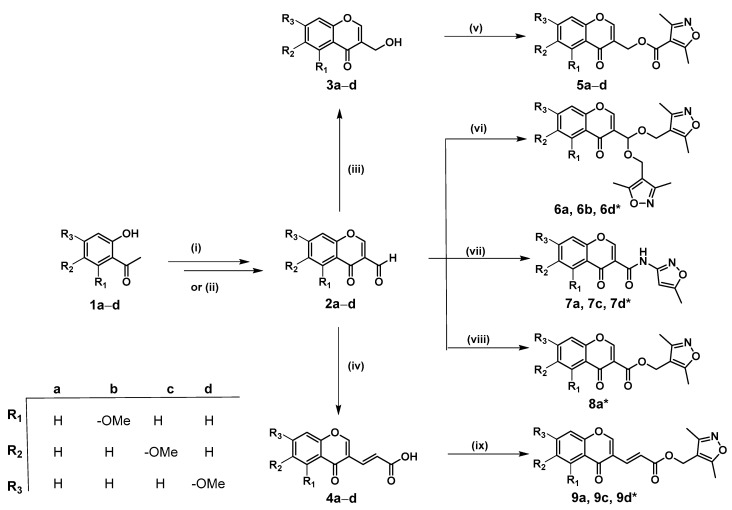
Synthesis of benzopyran-4-one-isoxazole conjugates. Reagents and Conditions: (i) POCl_3_, DMF, 0–60 °C, 13 h (**1**–**3**); (ii) (a) BF_3_-etherate, acetic anhydride, 100 °C, 1 h; (b) POCl_3_, DMF, 0–100 °C, 1 h (**4**); (iii) Basic alumina, 2-propanol, 75 °C, 4 h; (iv) Malonic acid, pyridine, 110 °C, 1 h; (v) 3,5-Dimethylisoxazole-4-carboxylic acid, EDCI, DMAP, DMF, 0 °C-rt, 24 h; (vi) (3,5-Dimethyl-isoxazol-4-yl)-methanol, InF_3_, toluene, 110 °C, 4 h; (vii) (a) NBS, AIBN, 100 °C, 1 h; (b) 3-Amino-5-methylisoxazole, 0 °C-rt, 0.5 h; (viii) (a) NBS, AIBN, 100 °C, 1 h; (b) (3,5-Dimethyl-isoxazol-4-yl)-methanol, 0 °C-rt, 0.5 h; (ix) EDCI, DMAP, DMF, 0 °C, (3,5-dimethyl-isoxazol-4-yl)-methanol, rt, 24 h. * Compounds **6c**, **7b**, **8b**-**d**, and **9b** were not obtained via the synthetic protocols followed due to changes in the reactivity of the intermediates on -OMe substitution at different positions on the chromone nucleus.

Scheme 1 depicts the synthetic strategies used for benzopyran-4-one-isoxazole conjugates. They were synthesized in two to three steps, i.e., first, the unsubstituted/substituted (R = -H/-OMe) 3-formyl benzopyran-4-ones were synthesized from the substituted ortho-hydroxy acetophenones (**1a**–**d**) and a formylating reagent (POCl_3_-DMF) via Vilsmeier–Haack reaction, respectively [34]. Benzopyran-4-one-3-carbaldehyde (**2a**) and 5/6-methoxy-benzopyran-4-one-3-carbaldehydes (**2b**,**c**) were synthesized readily and in good yields via the normal Vilsmeier–Haack formylation reaction. However, the yields and purity of 7-methoxy benzopyran-4-one-3-carbaldehyde (**2d**) were very low, so a modified two-step Vilsmeier–Haack reaction, according to the literature procedures, was used to improve the yield and purity of the Compound **2d** [35].

In the second step, the 3-formyl group of benzopyran-4-one-3-carbaldehydes (**2a**–**d**) was further modified to generate either a hydroxy methyl group (**3a**–**d**) or an enoic acid group (**4a**–**d**). The unmodified (**2a**–**d**)/modified benzopyran-4-one-3-carbaldehydes (**3a**–**d**, **4a**–**d**) were then further reacted with various methyl-substituted isoxazole derivatives under different reaction conditions to generate a library of 14 compounds with different linkers containing unsubstituted and methoxy substituted benzopyran-4-one rings (Scheme 1).

A total of 14 benzopyran-4-one-isoxazole conjugates were synthesized, out of which 13 are new, and one is known (**5c**), while out of 12 benzopyran-4-one precursors, 11 are reported in the literature. A few of the proposed hybrid compounds (*6c, **7b**, **8b**–**d**, and **9b**) were not obtained via the synthetic protocols followed due to the changes in the reactivity of the intermediates (**2a**–**d** and **4a**–**d**) on -OMe substitution at different positions on the benzopyran-4-one nucleus. Compounds **5a**–**d**, **6a**, **6b**, **6d**, **7a**, **7c**, **7d**, **8a**, **9a**, **9c**, and **9d** were synthesized and purified with the laboratories techniques and were further fully characterized on the basis of their ^1^H NMR, ^13^C NMR, ^13^C-DEPT NMR, IR, and Q-TOF mass spectral data (cf. Supplementary Materials (SM), page no. 2–65). The structures of known compounds were confirmed via a comparison of their spectral data with those reported in the literature.

Compounds **5a**–**d** were analyzed via high-performance liquid chromatography (HPLC) at λ_max_ 254 nm, using two different methods to check the purity and retention time. All four compounds showed a single peak with different retention times (cf. Supplementary Figures S27.1–S27.4 and Table S1, page no. 66–70), indicating high purity.

### 2.2. Biological Evaluation

#### 2.2.1. In Vitro Cytotoxicity against Cancer Cells

**Figure 2:**
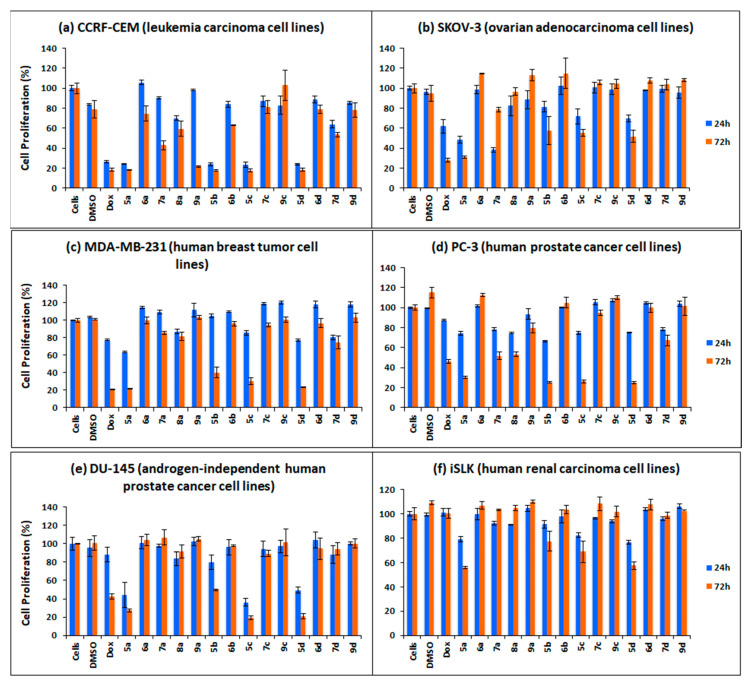
In vitro cytotoxicity of benzopyran-4-one-isoxazole conjugates (at a concentration of 25 μM) towards (**a**) human leukemia carcinoma (CCRF-CEM), (**b**) human ovarian adenocarcinoma (SKOV-3), (**c**) human breast tumor (MDA-MB-231), (**d**) human prostate cancer (PC-3), (**e**) androgen-independent human prostate cancer (DU-145), and (**f**) human renal carcinoma (iSLK) cell lines using DMSO and standard anticancer drug doxorubicin (Dox) (at a concentration of 5 μM) as controls after 24 and 72 h. The results are shown as the percentage of control that has no compound (set at 100%). All the experiments were performed in triplicate.

All the 14 hybrid compounds synthesized via Scheme 1, along with the intermediates and starting compounds, were tested in vitro for their cytotoxicity against a panel of 6 human cancer cell lines. This panel involved human leukemia carcinoma (CCRF-CEM), human ovarian adenocarcinoma (SKOV-3), human breast tumor (MDA-MB-231), human prostate cancer (PC-3), androgen-independent human prostate cancer (DU-145), and human renal carcinoma (iSLK) cell lines. The compounds were tested at a concentration of 25 μM for incubation periods of 24 and 72 h. Standard anticancer drug doxorubicin at a concentration of 5 μM and DMSO were used as controls in all the cell assays. The antiproliferative profile of benzopyran-4-one-isoxazole conjugates is shown in Figure 2 (cf. Supplementary Materials for the data obtained for intermediates and starting materials Figure S28.1–S28.6, page no. 71–76).

Among all the 14 compounds tested, a series of benzopyran-4-one-isoxazole esters, i.e., **5a**, **5b**, **5c,** and **5d**, exhibited selective antiproliferative activity against the panel of all six cancer cell lines tested (Figure 2a–f). Although among them, compound **5a** exhibited exceptional selectivity and cytotoxicity in all the cell lines tested. However, these conjugates, corresponding intermediates, and starting materials were almost inactive in the six tested cancer cell lines (cf. Supplementary Materials, Figure S28.1–S28.6, page no. 71–76). Table S2 (cf. Supplementary Materials, page no. 79) highlights the percentage inhibition of the cancer cell lines on treatment with doxorubicin (at a concentration of 5 μM) and conjugates **5a**–**d** (at a concentration of 25 μM) after 72 h of incubation.

The structure–activity relationship of the benzopyran-4-one—isoxazole hybrid compounds was evaluated from the data in Table S2 (Supplementary Materials, page no. 79) and Figure 2, which clearly indicates that the conjugation of benzopyran-4-one with isoxazole via a particular ester linkage, i.e., esterification of 3,5-dimethylisoxazole-4-carboxylic acid with 3-(hydroxymethyl) substituted-4-oxo-4*H*-1-benzopyrane (**5a**–**d**) had significantly higher selectivity toward the cancer cell lines compared to the hybrid compounds obtained via other chemical linkers, such as acetal (**6a**, **6b**, and **6d**), amide (**7a**, **7c**, and **7d**), reverse ester (**8a**), and acrylic ester (**9a**, **9c**, and **9d**). Furthermore, the structure–activity relationship indicates that substitution of a methoxy group at 5/6/7- position of benzopyran-4-one moiety decreases the activity slightly (**5b**, **5c**, and **5d**), while no substitution on the aromatic ring (**5a**) leads to higher selectivity and cytotoxicity against the cancer cell lines tested. Compounds **5a**–**d** led to high percentage inhibition (81–83%) of CCRF-CEM cancer cells, while a moderately high percentage of antiproliferative activity for the other five cell lines. Comparing the data for **5a** with **5b**–**d**, compound **5a** had the highest percentage of inhibition (44–81%) among this series of compounds.

#### 2.2.2. In Vitro Cytotoxicity against the Non-Cancerous Cell Lines

**Figure 3:**
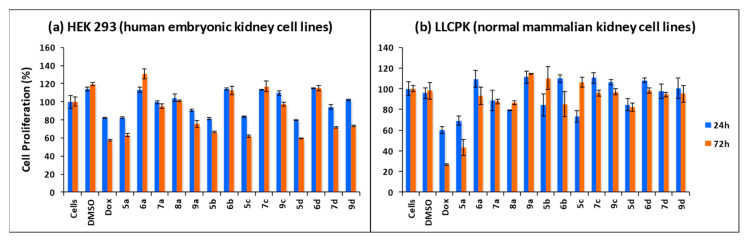
In vitro cytotoxicity of benzopyran-4-one-isoxazole conjugates (at a concentration of 25 μM) towards (**a**) human embryonic kidney (HEK-293) and (**b**) normal pig kidney (LLCPK) cell lines using DMSO and standard anticancer drug doxorubicin (at a concentration of 5 μM) as controls after 24 and 72 h. The results are shown as the percentage of control that has no compound (set at 100%). All the experiments were performed in triplicate.

The compounds were further screened for their in vitro cytotoxicity against non-cancerous normal cell lines, viz., human embryonic kidney (HEK-293) and normal pig kidney (LLCPK) cell lines. These cell lines were selected to determine whether the compounds had selectivity against cancerous cells in contrast to non-cancerous cells. The data obtained is shown in Figure 3a–b (cf. Supplementary Materials for the data obtained for intermediates and starting materials Figure S28.7–S28.8, page no. 77–78).

The inhibition percentages of the non-cancerous/normal cell lines on treatment with Dox (at a concentration of 5 μM) and conjugates **5a**–**d** (at a concentration of 25 μM) are represented in Table S3 (cf. Supplementary Materials, page no. 80). The data obtained clearly indicates the selectivity of this series of conjugates (**5a**–**d**) towards cancer cell lines (Table S2, cf. Supplementary Materials, page no. 79) in contrast to non-cancerous cell lines. From the data in Table S3 (cf. Supplementary Materials, page no. 80), it can be inferred that these compounds show a considerable safety margin towards non-cancerous/normal cells in contrast to cancerous cells when tested under identical experimental conditions.

#### 2.2.3. Half-Maximal Inhibitory Concentration (IC_50_)

Compounds **5a**–**d** exhibited potent growth inhibitory activity against a panel of six cancer cell lines and weak to no inhibitory activity against two normal cell lines. These compounds were further analyzed for their IC_50_ values against five selected cell lines. The data is displayed in Table 1. The compounds displayed weak activity on embryonic kidney (HEK-293) cell lines (>100–200 μM). However, compounds **5a**–**d** displayed potent growth inhibitory activity against all other four cell lines (3–51 μM), again confirming their selectivity towards cancerous cells in contrast to non-cancerous/normal cells. For example, compound **5a** showed potent antiproliferative activity against all the cancer cell lines with IC_50_ in the range of 5.6–17.84 μM and IC_50_ of 293.2 μM against normal cell lines (HEK-293). Similarly, compound **5c** was also potent against all the cancer cell lines with IC_50_ in the range of 3.3–12.92 μM and IC_50_ of 222.1 μM against normal cell lines (HEK-293). However, compounds **5b** and **5d** were a few folds less active against respective cancer cell lines (IC_50_ = 14.77–51.15 μM for **5b** and IC_50_ 5.2–16.1 μM for **5d**) and had low antiproliferative activity against normal HEK-293 cell lines (IC_50_ were in the range of 102.4 and 191.5 μM, respectively). Furthermore, compound **5c** showed 3–12-fold potency for CCRF-CEM cell lines compared to **5a**, **5b**, and **5d**, whereas compound **5a** showed 2–4-fold potency for MDA-MB-231 cell lines compared to **5c** and **5d**. Compounds **5a**, **5c**, and **5d** were similar in potency against PC3 and DU-145 compared to **5b**, which showed threefold weaker activity against DU-145 cell lines. Overall, the safety index of compounds was more than 10-fold based on IC_50_ against cancer and normal cell lines.

#### 2.2.4. NCI-60 Cell Lines Test

**Figure 4:**
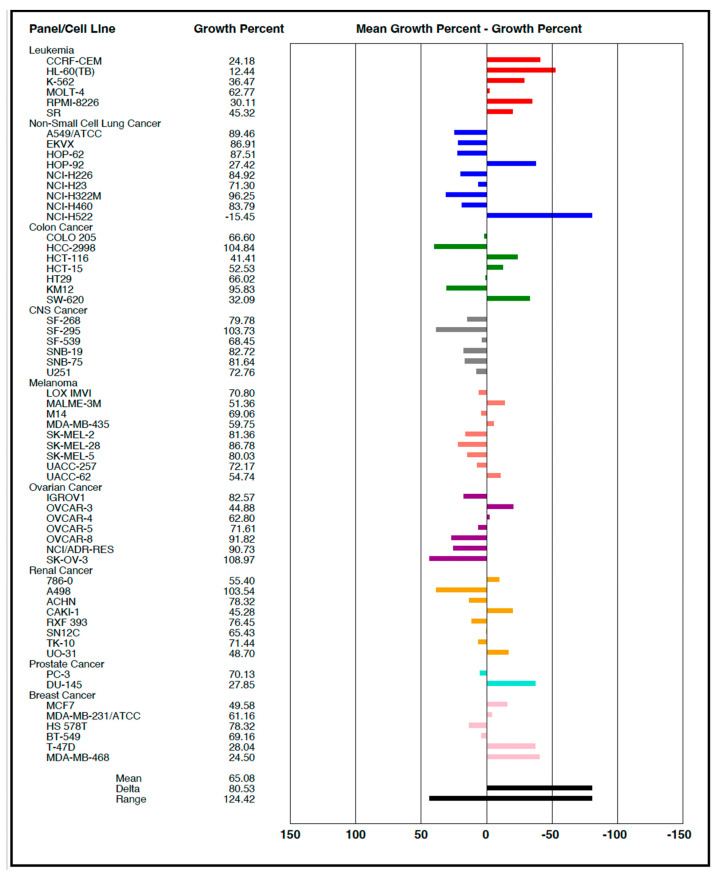
In vitro cytotoxicity activity of benzopyran-4-one-isoxazole conjugates (at a concentration of 10 μM) towards 60 different human cancer cell lines.

Among all the compounds tested, the best antiproliferative results were obtained from compound **5a**. To further investigate the potency of this compound against different cancer cell lines, it was screened in a single dose (10 μM) experiment on 60 different cancer cell lines by NCI, USA (Figure 4). The data on screening of compound **5a** against 60 cancer cell lines for leukemia, non-small lung, colon, CNS, melanoma, ovarian, renal, prostate, and breast cancer cell lines showed the potency of compound **5a** at a dose of 10 μM against leukemia and breast cancer cell lines. The results reveal that compound **5a** could serve as a feasible target (Figure 4) for further investigation in the next round of studies.

### 2.3. Kinase Inhibition Assay

Substituted benzopyran analogs have demonstrated protein kinase inhibitory activity [36]. In our previous studies [37], 4-oxo-4*H*-1-benzopyran derivatives showed Src kinase inhibitory activity with the IC_50_ of 52–57 µM. LY294002 has been found to be a selective inhibitor for phosphatidylinositol 3-kinase (PI3K) inhibitor with a cellular IC_50_ of about 1.4 µM. Therefore, selected compound **5a** was screened against several protein kinases to explore the potential of these hybrid compounds as kinase inhibitors. The screening of compound **5a** was performed on a panel of 9 kinases, viz., ABL1, c-Kit, c-Src, CDK2/cyclin A1, CSK, EGFR, mTOR/FRAP1, p38a/MAPK14, PKCa, and PI3K, at a dose of 20 μM. The respective positive controls were used for each kinase, and the kinase reaction was performed at 200 μM ATP concentration for all the kinases except for PI3K, which was performed at 10 µM ATP (Table 2). The results indicated that compound **5a** was found to be inactive towards all the kinases screened. It appeared that compound **5a** containing a 3-position substitution with an isoxazole ring and a 4-position with a carbonyl group has a detrimental effect on kinase inhibition. Previously 3-substituted benzopyran compound with a short alkyl chain of urea (NH-CO-NH-Et/Me/iPr) was reported with Abl kinase inhibitory activity in the range of 4–27 μM [36]. While compound **5a**, containing an ester linker and 2,4-methyl isoxazole moiety at the 3-substitution on the benzopyran ring, showed a detrimental effect due to a lack of hydrogen bond interaction with the key residues in the kinase enzyme binding pocket. Most active benzopyran analogs with kinase inhibitory activity have 2-position substitution, such as LY294002 [38]. Due to prominent antiproliferative activity, these compounds may have some other targets for anticancer activity.

### 2.4. Serum Stability

These compounds were further explored towards degradation by proteolytic enzymes towards degradation. Compound **5a** was investigated for proteolytic stability in the presence of human serum. It was incubated with human serum at 37 °C, and aliquots of the reaction mixture were analyzed via HPLC at different time points up to 2 h. The concentration of Compound **5a** was measured by integrating the area under the curve (AUC) and correspondingly calculated from the standard curve of **5a** at different concentrations (cf. Supplementary Materials, page no. 81–82).

**Figure 5:**
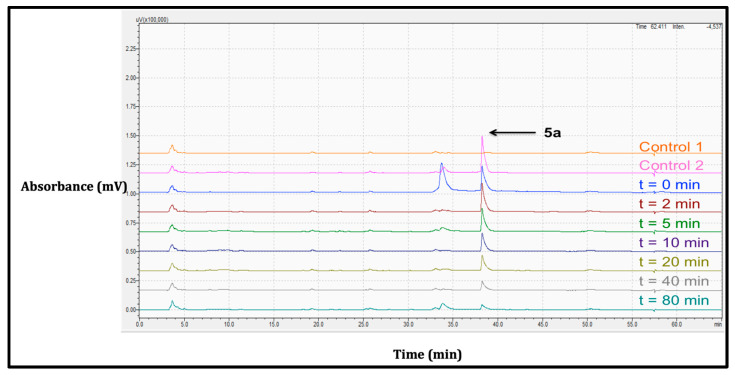
RP-HPLC chromatograms of conjugate **5a** after incubation with human serum. The Conjugate was incubated with human serum for different time intervals of 0, 2, 5, 10, 20, 40, and 80 min before HPLC analysis. Conjugate **5a** appears at 38 min, which degrades with time. Degradation products appear at around 34 min.

The HPLC chromatogram of serum incubated with compound **5a** for 80 min was illustrated in Figure 5. Compound **5a** was completely degraded after 2 h of incubation with human serum. Aliquots from the reaction solution were taken at intervals of 0, 2, 5, 10, 20, 40, 80, and 120 min after incubation with human serum.

**Figure 6:**
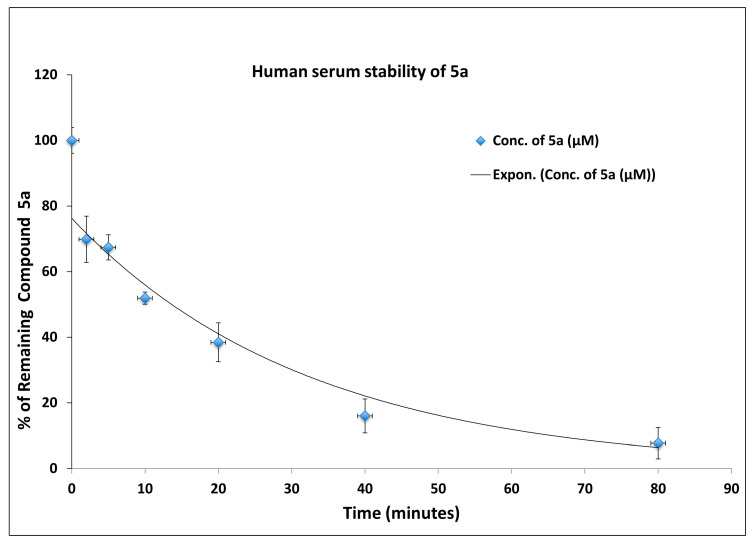
Human serum stability of **5a** at 37 °C at various time intervals (n = 3).

Figure 6 shows the plots of human serum stability of **5a** vs. % remaining of compound **5a,** and the logarithm of the concentration of **5a** vs. time in minutes (cf. Supplementary Materials, page no. 85) for the calculation of the half-life of **5a** in human serum. The results indicate that Compound **5a** has a half-life of nearly 13.6 min (cf. Supplementary Materials, page no. 84). The short half-life is an indication that this series of compounds stay for a short period in physiological conditions, which means that there is a further need for modification for the next generation of benzopyran-4-one-isoxazole hybrid compounds to increase their stability in human serum.

### 2.5. Apoptosis Detection by Flow Cytometry Analysis

**Figure 7:**
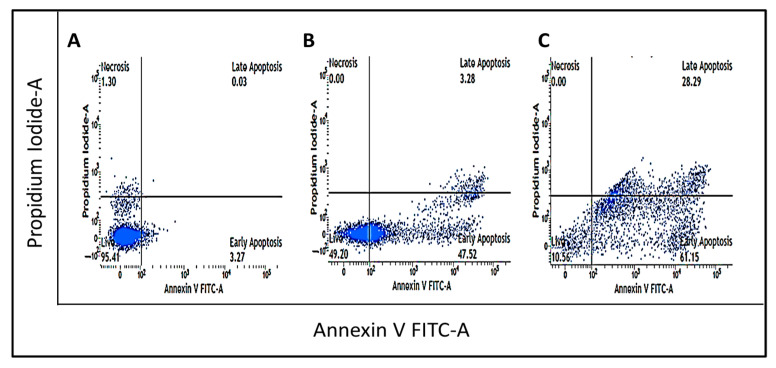
Annexin V-FITC analysis of MDA-MB-231 cells untreated (**A**), treated with **5a** (5 μM) (**B**), and doxorubicin (5 μM) (**C**) for 72 h. Results represent one of three independent experiments.

**Figure 8:**
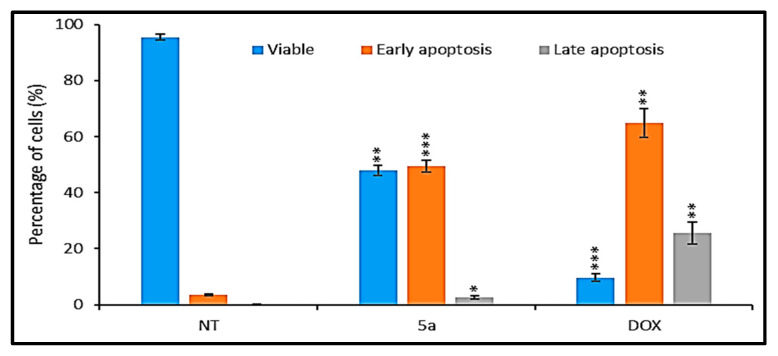
MDA-MB-231 cell distribution after the treatment with **5a** (5μM) and doxorubicin (DOX) (5 μM) for 72 h. Values are presented as means ± SD of triplicate experiments. * *p* < 0.05, ** *p* < 0.01, and *** *p* < 0.001 compared with the non-treated (NT) control cells.

In accordance with the MTS assay, compound **5a** had promising antiproliferative action. Therefore, we attempted to study the mechanism triggering cell death. It is established that inducing apoptosis is one of the most important methods to lead to cell death via chemotherapy; hence, we performed phosphatidylserine cytosine analysis to check the induction of apoptosis by compound **5a** on MDA-MB-231 cells. MDA-MB-231 cells were treated with 5 μM of compound **5a** and Dox (5 μM) as a positive control and incubated for 72 h, then double-stained carefully by membrane-linked protein Annexin-V fluorescein isothiocyanate (FITC) and propidium iodide (PI) for flow cytometry analysis. Normally, in the early stages of apoptosis, phosphatidylserine flips from the inner cell membrane to the surface of the cell membrane. The exposed phosphatidylserine could be recognized by an extracellular calcium-dependent phospholipid-binding protein Annexin-V; hence, FITC-labeled Annexin-V could bind specifically to phosphatidylserine and be used to detect apoptosis cells by flow cytometry. PI is a nucleic acid dye that can only cross the membrane of advanced apoptosis and necrotic cells and redden the nucleus. Consequently, when the treated cells are double stained by the two fluorescent dyes, early and late apoptosis and necrotic cells can be clearly distinguished. As shown in Figure 7, untreated control MDA-MB-231 cells had high observed viability of 95.41% (Figure 7A). After the treatment with compound **5a** (5 μM), the percentage of apoptosis cells increased from 3.27% (in untreated control MDA-MB-231 cells) to 47.52% for early apoptosis and from 0.03% (in untreated control MDA-MB-231 cells) to 3.28% for late apoptosis (Figure 7B); in comparison, doxorubicin (5 μM) induced 61.15% for early apoptosis and 28.29% for late apoptosis to MDA-MB-231 cells (Figure 7C). These findings illustrated that compound **5a** mainly leads to the early apoptosis of MDA-MB-231 cells. The comparison of MDA-MB-231 cell distribution after **5a** and doxorubicin treatment 72 h was shown in Figure 8.

## 3. Materials and Methods

### 3.1. Instrumentation

Nuclear magnetic resonance spectroscopy: The ^1^H NMR and ^13^C NMR spectra were recorded on Bruker Acend 400 spectrometer (Billerica, MA, USA), operating at 400.15 MHz for ^1^H and 100.62 MHz for ^13^C. The spectra were calibrated using the solvent peaks. The chemical shift values were expressed in δ (ppm) scale relative to tetramethylsilane (TMS) as an internal reference, and the coupling constant values (*J*) were given in Hz. Assignments were also made from DEPT (distortionless enhancement by polarization transfer) (underlined values).

Infrared spectroscopy: Infrared spectra were recorded on Bruker Alpha FT-IR spectrophotometer (Billerica, MA, USA); the frequency values were recorded in cm^−1^ scale.

Mass spectroscopy: The mass spectra were recorded on Bruker Q-TOF LC/MS mass spectrophotometer (Billerica, MA, USA); the data were reported as *m*/*z* (% of relative intensity of the most important fragments).

UV-Vis Spectroscopy: UV data were recorded in methanol on Shimadzu, UV-2600/2700 UV-VIS recording spectrophotometer (Shimadzu Corporation, MD, USA).

Analytical HPLC: The purity of the final products (>97% purity) was verified by high-performance liquid chromatography (HPLC) equipped with a UV detector. Chromatograms were obtained in an HPLC/DAD system, Jasco instrument (pumps model 880-PU and solvent mixing model 880–30, Tokyo, Japan), equipped with a commercially prepacked Nucleosil RP-18 analytical column (250 mm Å~4.6 mm, 5 μm, Macherey-Nagel, Duren, Germany) and UV detection (Jasco model 875-UV) at the maximum wavelength of 254 nm. The mobile phase consisted of acetonitrile/water (gradient mode, room temperature) at a flow rate of 1 mL/min. The chromatographic data were processed with a Compaq computer fitted with CSW 1.7 software (DataApex, Podohradska, Czech Republic).

### 3.2. Materials

2′-Hydroxyacetophenone was supplied by Acros Organics; 2′-hydroxy-6′-methoxyacetophenone, 2′-hydroxy-5′-methoxyacetophenone, and 2′-hydroxy-4′-methoxyacetophenone, 3,5-dimethylisoxazole-4-carboxylic acid by Alfa Aesar; 3-amino-5-methyisoxazole by TCI and (3,5-dimethyl-isoxazol-4-yl)-methanol by Oakwood chemicals Inc, Estill, SC, USA, respectively. All other chemicals and solvents used for the conjugate synthesis were of analytical grade and purchased from Sigma-Aldrich, Milwaukee, WI, USA. Analytical TLCs were performed on precoated Merck silica gel 60 F254 (Merck) plates with layer thickness of 0.2 mm. For analytical control, ethyl acetate/petroleum ether system was used. The spots were visualized under UV detection (254 nm and 366 nm), iodine vapor, and also by spraying with potassium permanganate stain (prepared by dissolving 1.5 g of KMNO_4_, 10 g K_2_CO_3_, and 1.25 mL 10% NaOH in 200 mL water). Column chromatography was performed on silica gel (100–200 mesh) provided by Merck (MilliporeSigma, Burlington, MA, USA) or on aluminum oxide, activated, basic from Sigma Aldrich. Following the workup, the organic phases were dried over Na_2_SO_4_. The recrystallization solvents were ethyl acetate, dichloromethane, or ethyl ether/n-hexane. Solvents were evaporated on a Buchi rotavapor, Buchi Corporation, New Castle, DE, USA.

### 3.3. Synthesis

#### 3.3.1. General Procedure for the Synthesis of 3-Formylbenzopyran-4-one (**2a**–**2c**) [39,40]

A solution of substituted (R = H/OMe) *o*-hydroxyacetophenone (**1a**/**1b**/**1c**, 0.1 mol) was dissolved in anhydrous DMF (80 mL), placed in a two-neck round bottom flask, and freshly distilled POCl_3_ (80 mL) was added dropwise through a dropping funnel over 30 min with vigorous stirring in an ice-water bath maintained at 0–5 °C. The reaction mixture was then stirred at 60 °C for 13 h. The reaction was monitored by TLC [ethyl acetate–petroleum ether 50:50] to completion, and the mixture was decomposed by pouring over 500 mL of ice water after the reaction was completed. The resulting residue was collected by filtration, washed with water, and recrystallized from ethanol–water. The 3 formyl-substituted benzopyran-4-ones are listed below.

*4-Oxo-4H-1-benzopyran-3-yl-carboxaldehyde* (**2a**). Yield = 69%; *R*_f_ = 0.72 [ethyl acetate–petroleum ether 50:50]; IR (neat) ν_max_: 3715, 3696, 3058, 2971, 2868, 2845, 2825, 2365, 2341, 2170, 2033, 1979, 1694, 1645, 1613, 1562, 1462, 1418, 1363, 1338, 1311, 1271, 1236, 1192, 1148, 1055, 1032, 1012, 849, 767, 744 and 690 cm^−1^; UV/vis (MeOH) λ: 297, 238 nm; ^1^H NMR (400 MHz, CDCl_3_): δ 10.37 (s, 1H, C*H*O), 8.53 (s, 1H, H-2), 8.28 (dd, *J* = 7.9, 1.5 Hz, 1H, H-5), 7.75 (ddd, *J* = 8.7, 7.2, 1.7 Hz, 1H, H-7), 7.56–7.46 (m, 2H, H-6 and H-8); ^13^C NMR (101 MHz, CDCl_3_): δ 188.61 (CHO), 175.98 (C-4 C=O), 160.63 (C-2), 156.21 (C-8a), 134.84 (C-7), 126.66(C-6), 126.18 (C-5), 125.32 (C-4a), 120.33 (C-3), 118.63 (C-8); HRMS (ESI-TOF) (*m*/*z*) C_10_H_6_O_3_ calculated, 197.021, found, 197.140 [M + Na]^+^; calculated, 212.995, found, 213.125 [M + K]^+^ [41].

*5-Methoxy-4-oxo-4H-1-benzopyran-3-carboxaldehyde* (**2b**). Yield = 89%; *R*_f_ = 0.31 [ethyl acetate–petroleum ether 50:50]; IR (neat) ν_max_: 3708, 3679, 2970, 2923, 2866, 2844, 2826, 2166, 2074, 2050, 2027, 1977, 1963, 1698, 1653, 1610, 1562, 1539, 1476, 1456, 1434, 1415, 1345, 1272, 1055, 1032, 1013 and 765 cm^−1^; UV/vis (MeOH) λ: 315, 256 nm; ^1^H NMR (400 MHz, CDCl_3_): δ 10.35 (s, 1H, CHO), 8.39 (s, 1H, H-2), 7.62 (t, *J* = 8.4 Hz, 1H, H-7), 7.07 (dd, *J* = 8.4, 0.7 Hz, 1H, H-8), 6.90 (d, *J* = 8.4 Hz, 1H, H-6), 4.01 (s, 3H, CH_3_); ^13^C NMR (101 MHz, CDCl_3_): δ 189.30 (CHO), 175.97 (C-4 C=O), 160.51 (C-5), 158.88 (C-2), 158.23 (C-8a), 135.08 (C-7), 121.38 (C-3), 115.71 (C-4a), 110.56 (C-6), 108.08 (C-8), 56.72 (OCH_3_); HRMS (ESI-TOF) (*m*/*z*) C_11_H_8_O_4_ calculated, 205.050, found 205.128 [M + H]^+^; calculated, 227.032, found, 227.119 [M + Na]^+^; and calculated, 243.006, found, 243.100 [M + K]^+^ [40,42].

*6-Methoxy-4-oxo-4H-1-benzopyran-3-carboxaldehyde* (**2c**). Yield = 51%; *R*_f_ = 0.73 [ethyl acetate–petroleum ether 50:50]; IR (neat) ν_max_: 3710, 3697, 3656, 3635, 2970, 2940, 2923, 2866, 2844, 2826, 2362, 2342, 2165, 2073, 2050, 2025, 1977, 1652, 1481, 1457, 1437, 1345, 1321, 1055, 1032 and 1014 cm^−1^; UV/vis (MeOH) λ: 324, 240 nm; ^1^H NMR (400 MHz, CDCl_3_): δ 10.39 (s, 1H, C*H*O), 8.52 (s, 1H, H-2), 7.63 (d, *J* = 2.9 Hz, 1H, H-5), 7.46 (d, *J* = 9.1 Hz, 1H, H-8), 7.31 (dd, *J* = 9.1, 3.0 Hz, 1H, H-7), 3.91 (s, 3H, -OCH_3_); ^13^C NMR (101 MHz, CDCl_3_): δ 188.91 (CHO), 176.01 (C-4 C=O), 160.36 (C-2), 158.08 (C-6), 151.09 (C-8a), 126.25 (C-4a), 124.60 (C-7), 120.13 (C-8), 119.73 (C-3), 105.58 (C-5), 56.18 (OCH_3_); HRMS (ESI-TOF) (*m*/*z*) C_11_H_8_O_4_ calculated, 205.050, found 205.105 [M + H]^+^, and calculated, 227.032, found, 227.093 [M + Na]^+^ [40,43].

#### 3.3.2. 7-Methoxy-4-oxo-4*H*-1-benzopyran-3-carboxaldehyde (**2d**)

7-methoxy-4-oxo-4*H*-1-benzopyran-3-carbaldehyde was synthesized according to the literature-reported procedure [25]. A solution of 2′-hydroxy-4′-methoxyacetophenone 1d (3.8 g, 23 mmol), BF_3_-etharate (5.6 mL, 46 mmol), and acetic anhydride (8.6 mL, 92 mmol) was taken in the round bottom flask. The reaction mixture was heated at 100 °C for 1 h. The precipitate was collected and washed 3 times with diethyl ether. The resulting precipitate was filtered to afford difluorodioxaborin as brown-colored crystals. Yield = 98.15%; ^1^H NMR (400 MHz, CDCl_3_) δ 7.63 (d, *J* = 9.2 Hz, 1H, H-5), 6.58 (dd, *J* = 9.2, 2.4 Hz, 1H, H-6), 6.46 (d, *J* = 2.4 Hz, 1H, H-8), 3.93 (s, 3H, -OCH_3_), 2.73 (s, 3H, -CH_3_). In the second step, POCl_3_ (5.6 mL) was added to 20 mL of DMF at 0 °C, followed by the addition of difluorodioxaborin (4.8 g, 22 mmol) in 5 mL DMF. The reaction mixture was stirred at 100 °C for 1 h and then poured on crushed ice (250 g). The mixture was then refrigerated overnight. The resulting precipitate was filtered and washed with water. The crude product was recrystallized from acetone to afford (2d) as brown-colored crystals. Yield = 64%; *R*_f_ = 0.67 [ethyl acetate–petroleum ether 50:50]; IR (neat) ν_max_: 3712, 3696, 3655, 3635, 2969, 2923, 2866, 2844, 2827, 2362, 2342, 2165, 2074, 2027, 1978, 1703, 1653, 1619, 1559, 1528, 1502, 1439, 1363, 1347, 1304, 1277, 1244, 1187, 1141, 1055, 1031, 1015, 935, 841, 800 and 765 cm^−1^; UV/vis (MeOH) λ: 291, 247, 241 nm; ^1^H NMR (400 MHz, CDCl_3_): δ 10.37 (s, 1H, C*H*O), 8.47 (s, 1H, H-2), 8.20 (d, *J* = 8.9 Hz, 1H, H-5), 7.05 (dd, *J* = 8.9, 2.4 Hz, 1H, H-6), 6.91 (d, *J* = 2.4 Hz, 1H, H-8), 3.93 (s, 3H, -OCH_3_); ^13^C NMR (101 MHz, CDCl_3_): δ 189.06 (CHO), 175.47 (C-4 C=O), 165.05 (C-2), 160.39 (C-7), 158.13 (C-8a), 127.69 (C-5), 120.40 (C-3), 118.98 (C-8a), 115.68 (C-6), 101.27 (C-8), 56.17 (OCH_3_); HRMS (ESI-TOF) (*m*/*z*) C_11_H_8_O_4_ calculated, 205.050, found 205.147 [M + H]^+^, calculated, 227.032, found, 227.140 [M + Na]^+^; and calculated, 243.006, found, 243.121 [M + K]^+^ [40].

#### 3.3.3. General Procedure for the Synthesis of 3-(Hydroxymethyl) Substituted-4-oxo-4*H*-1-benzopyrane (**3a**–**3d**) [43]

A mixture of 4-oxo-4*H*-1-benzopyran-3-yl-carboxaldehyde (**2a**, 5 g, 28.7 mmol) or methoxy-substituted 4-oxo-4*H*-1-benzopyran-3-yl-carboxaldehyde (**2b**/**2c**/**2d**, 5 g, 24.5 mmol), basic alumina (50 g) and 2-propanol (300 mL) were taken in a round bottom flask. The reaction mixture was heated at 75 °C for 4 h. The reaction was monitored by TLC [ethyl acetate–petroleum ether 50:50] to completion, and then the reaction mixture was filtered by pouring over celite. The solvent was evaporated in vacuum, and the resulting precipitate was purified by column chromatography to afford **3a**–**3d** as yellow solid. The substituted 3-(hydroxymethyl) benzopyran-4-ones are listed below:

*3-(Hydroxymethyl)-4-oxo-4H-1-benzopyrane* (**3a**). Yield = 52%; *R*_f_ = 0.41 [ethyl acetate–petroleum ether 50:50]; IR (neat) ν_max_: 3359, 3088, 2952, 2723, 2367, 2343, 1635, 1605, 1571, 1466, 1425, 1407, 1350, 1315, 1271, 1234, 1216, 1187, 1162, 1109, 1062, 1025, 974, 946, 927, 847, 805, 758, 724, 708, 686 and 632 cm^−1^; UV/vis (MeOH) λ: 296, 249 nm; ^1^H NMR (400 MHz, CDCl_3_): δ 8.22 (dd, *J* = 8.0, 1.7 Hz, 1H, H-5), 7.95 (s, 1H, H-2), 7.69 (ddd, *J* = 8.7, 7.1, 1.7 Hz, 1H, H-7), 7.55–7.36 (m, 2H, H-6 and H-8), 4.59 (s, 1H, -C*H_2_*OH), 2.85 (brs, 1H, OH); ^13^C NMR (101 MHz, CDCl_3_): δ 178.65 (C-4 C=O), 156.75 (C-8a), 152.88 (C-2), 134.10 (C-7), 125.75 (C-5), 125.45 (C-6), 123.98 (C-4a), 123.40 (C-3), 118.39 (C-8), 58.89 (CH_2_OH); HRMS (ESI-TOF) (*m*/*z*) C_10_H_8_O_3_ calculated, 199.037, found 199.109 [M + Na]^+^ [43].

*3-(Hydroxymethyl)-5-methoxy-4-oxo-4H-1-benzopyrane* (**3b**). Yield = 33%; *R*_f_ = 0.07 [ethyl acetate–petroleum ether 50:50]; IR (neat) ν_max_: 3698, 3679, 3662, 3427, 2969, 2922, 2866, 2844, 2827, 2361, 2341, 2074, 2027, 1644, 1605, 1566, 1547, 1526, 1512, 1475, 1436, 1347, 1297, 1269, 1182, 1160, 1055, 1032, 1013, 893, 804 and 694 cm^−1^; UV/vis (MeOH) λ: 315, 254, 226 nm; ^1^H NMR (400 MHz, CDCl_3_): δ 7.79 (s, 1H, H-2), 7.56 (t, *J* = 8.4 Hz, 1H, H-7), 7.02 (d, *J* = 8.5 Hz, 1H, H-8), 6.81 (d, *J* = 8.3 Hz, 1H, H-6), 4.50 (s, 2H, -*CH_2_*OH), 3.99 (s, 3H, -OCH_3_), 3.25 (s, 1H, -OH); ^13^C NMR (101 MHz, CDCl_3_): δ 178.85 (C-4 C=O), 160.05 (C-5), 158.78 (C-8a), 150.90 (C-2), 134.27 (C-7), 124.32 (C-3), 114.64 (C-4a), 110.40 (C-6), 106.44 (C-8), 59.20 (CH_2_OH), 56.56 (OCH_3_); HRMS (ESI-TOF) (*m*/*z*) C_11_H_10_O_4_ calculated, 207.066, found 207.061 [M + H]^+^; calculated, 229.048, found, 229.043 [M + Na]^+^; and calculated, 245.022, found, 245.017 [M + K]^+^ [44].

*3-(Hydroxymethyl)-6-methoxy-4-oxo-4H-1-benzopyrane* (**3c**). Yield = 43%; *R*_f_ = 0.32 [ethyl acetate–petroleum ether 50:50]; IR (neat) ν_max_: 3697, 3679, 3656, 3635, 3618, 3404, 2922, 2867, 2845, 2363, 2342, 2165, 2074, 2050, 1637, 1609, 1581, 1546, 1527, 1512, 1485, 1458, 1438, 1395, 1375, 1344, 1322, 1269, 1246, 1200, 1150, 1102, 1065, 1031, 1015, 891, 823, 766 and 707 cm^−1^; UV/vis (MeOH) λ: 324, 239, 231 nm; ^1^H NMR (400 MHz, CDCl_3_): δ 7.96 (s, 1H, H-2), 7.49 (d, *J* = 3.0 Hz, 1H, H-5), 7.36 (d, *J* = 9.2 Hz, 1H, H-8), 7.22 (dd, *J* = 9.2, 3.1 Hz, 1H, H-7), 4.50 (s, 2H, -C*H_2_*OH), 3.84 (s, 3H, -OCH_3_); ^13^C NMR (101 MHz, CDCl_3_): δ 178.24 (C-4 C=O), 157.05 (C-6), 153.41 (C-2), 151.64 (C-8a), 124.29 (C-4a), 124.22 (C-7), 122.68 (C-3), 119.74 (C-8), 104.48 (C-5), 57.47 (CH_2_OH), 55.89 (OCH_3_); HRMS (ESI-TOF) (*m*/*z*) C_11_H_10_O_4_ calculated, 207.0656, found 207.194 [M + H]^+^; and calculated, 229.048, found, 229.190 [M + Na]^+^ [43,44].

*3-(Hydroxymethyl)-7-methoxy-4H-1-benzopyran-4-one* (**3d**). Yield = 12%; *R*_f_ = 0.28 [ethyl acetate–petroleum ether 50:50]; IR (neat) ν_max_: 3713, 3696, 3655, 3635, 3618, 3403, 2922, 2867, 2845, 2363, 2342, 2165, 2073, 2030, 1977, 1641, 1605, 1565, 1546, 1524, 1496, 1442, 1400, 1376, 1344, 1319, 1275, 1242, 1206, 1174, 1095, 1055, 1032 and 1014 cm^−1^; UV/vis (MeOH) λ: 280, 247 nm; ^1^H NMR (400 MHz, CDCl_3_): δ 8.12 (d, *J* = 8.9 Hz, 1H, H-5), 7.86 (s, 1H, H-2), 6.99 (dd, *J* = 8.9, 2.4 Hz, 1H, H-6), 6.84 (d, *J* = 2.4 Hz, 1H, H-8), 4.56 (s, 2H, -OCH_2_-), 3.91 (s, 3H, -OCH_3_); ^13^C NMR (101 MHz, CDCl_3_): δ 178.07 (C-4 C=O), 164.46 (C-7), 158.61 (C-8a), 152.29 (C-2), 127.12 (C-5), 123.16 (C-3), 117.90 (C-4a), 114.99 (C-6), 100.40 (C-8), 59.01 (CH_2_OH), 55.99 (OCH_3_); HRMS (ESI-TOF) (*m*/*z*) C_11_H_10_O_4_ calculated, 229.048, found 228.949 [M + Na]^+^; and calculated, 245.022, found, 246.102 [M + K]^+^ [44].

#### 3.3.4. General Procedure for the Synthesis of Substituted (E)-3-(4-Oxo-4*H*-1-benzopyran-3-yl)acrylic Acid (**4a**–**4d**) [45]

A mixture of 4-oxo-4*H*-1-benzopyran-3-yl-carboxaldehyde (**2a**, 5 g, 28.7 mmol) or methoxy-substituted 4-oxo-4*H*-1-benzopyran-3-yl-carboxaldehyde (**2b**/**2c**/**2d**, 5 g, 24.5 mmol), malonic acid (6.2 g, 28.7 mmol for **2a**/5.3 g, 24.5 mmol for **2b**/**2c**/**2d**), and pyridine (20 mL) was heated at 110 °C. After an hour, additional malonic acid (3.1 g, 14.36 mmol for **2a**/2.6 g, 12.23 mmol for **2b**/**2c**/**2d**) was added to the reaction mixture. After 30 min, the solvent was evaporated in vacuum and the resulting precipitate was recrystallized from acetone to afford **4a**–**d**.

*(E)-3-(4-Oxo-4H-1-benzopyran-3-yl)acrylic acid* (**4a**). Yield = 74%; *R*_f_ = 0.69 [ethyl acetate]; IR (neat) ν_max_: 3364, 2955, 2919, 2848, 2666, 2367, 2343, 1659, 1611, 1562, 1547, 1464, 1425, 1410, 1360, 1323, 1300, 1249, 1015, 987, 858, 784, 760, 710, 684, 657 and 614 cm^−1^; UV/vis (MeOH) λ: 289, 266 nm; ^1^H NMR (400 MHz, DMSO): δ 12.34 (brs, 1H, -COO*H*), 8.87 (s, 1H, H-2), 8.13 (dd, *J* = 8.0, 1.5 Hz, 1H, H-5), 7.84 (ddd, *J* = 8.7, 7.1, 1.7 Hz, 1H, H-7), 7.70 (dd, *J* = 8.4, 0.6 Hz, 1H, H-8), 7.54 (ddd, *J* = 8.1, 7.2, 1.1 Hz, 1H, H-6), 7.43 (d, *J* = 15.9 Hz, 1H, H-1′), 7.12 (d, *J* = 15.9 Hz, 1H, H-2′);^13^C NMR (101 MHz, DMSO): δ 175.32 (C-4 C=O), 167.78 (C-3′ C=O), 159.83 (C-2), 155.12 (C-8a), 135.90 (C-1′), 134.56 (C-7), 126.08 (C-8), 125.45 (C-6), 123.51 (C-4a), 121.35 (C-5), 118.54 (C-3), 118.14 (C-2′); HRMS (ESI-TOF) (*m*/*z*) C_12_H_8_O_4_ calculated, 239.032, found 239.179 [M + Na]^+^; and calculated, 255.006, found, 255.163 [M + K]^+^ [46,47].

*(E)-3-(5-Methoxy-4-oxo-4H-1-benzopyran-3-yl)acrylic acid* (**4b**). Yield = 24%; *R*_f_ = 0.31 [ethyl acetate]; IR (neat) ν_max_: 3698, 3679, 3656, 3634, 3399, 2941, 2867, 2843, 2363, 2342, 2075, 1642, 1604, 1472, 1437, 1408, 1358, 1269, 1178, 1068, 1031, 1015, 872, 812, 776, 689, and 650 cm^−1^; UV/vis (MeOH) λ: 299, 254 nm; ^1^H NMR (400 MHz, MeOD): δ 8.42 (s, 1H, H-2), 7.68 (t, *J* = 8.4 Hz, 1H, H-7), 7.48 (d, *J* = 15.9 Hz, 1H, H-1′), 7.09 (m, 3H, H-2′, H-8 and H-6), 3.95 (s, 3H, -OCH_3_); ^13^C NMR (101 MHz, MeOD): δ 177.60 (C-4 C=O), 170.53 (C-3′ C=O), 161.46 (C-5), 159.06 (C-8a), 158.62 (C-2), 137.48 (C-1′), 136.11 (C-7), 122.05 (C-3), 121.11 (C-2′), 115.25 (C-4a), 111.15 (C-6), 108.63 (C-8), 56.75 (OCH_3_); HRMS (ESI-TOF) (*m*/*z*) C_13_H_10_O_5_ calculated, 247.061, found 247.214 [M + H]^+^; and calculated, 269.043, found, 269.211 [M + Na]^+^ [37].

*(E)-3-(6-Methoxy-4-oxo-4H-1-benzopyran-3-yl)acrylic acid* (**4c**). Yield = 81%; *R*_f_ = 0.72 [ethyl acetate]; IR (neat) ν_max_: 3696, 3679, 3655, 3634, 3346, 3064, 2970, 2866, 2844, 2362, 2342, 2165, 2074, 2050, 1695, 1646, 1611, 1564, 1547, 1484, 1454, 1436, 1421, 1395, 1374, 1343, 1320, 1296, 1212, 1197, 1159, 1055, 1031, 1014, 941, 867, 830, 791, 726, 678 and 610 cm^−1^; UV/vis (MeOH) λ: 245 nm; ^1^H NMR (400 MHz, DMSO): δ 12.37 (s, 1H, -COOH), 8.83 (s, 1H, H-2), 7.65 (d, *J* = 9.1 Hz, 1H, H-8), 7.47–7.40 (m, 3H, H-2′, H-7 and H-5), 7.10 (d, *J* = 15.9 Hz, 1H, H-1′), 3.86 (s, 3H, -OCH_3_); ^13^C NMR (101 MHz, DMSO): δ 174.96 (C-4 C=O), 167.81 (C-3′ C=O), 159.51 (C-2), 156.97 (C-6), 149.86 (C-8a), 136.02 (C-2′), 124.24 (C-4a), 123.53 (C-3), 121.11 (C-7), 120.14 (C-8), 117.31 (C-1′), 105.22 (C-5), 55.79 (OCH_3_); HRMS (ESI-TOF) (*m*/*z*) C_13_H_10_O_5_ calculated, 247.061, found 247.215 [M + H]^+^; calculated, 269.043, found, 269.211 [M + Na]^+^; and calculated, 285.017, found, 285.195 [M + K]^+^ [48].

*(E)-3-(7-Methoxy-4-oxo-4H-1-benzopyran-3-yl)acrylic acid* (**4d**). Yield = 75%; *R*_f_ = 0.66 [ethyl acetate]; IR (neat) ν_max_: 3696, 3678, 3655, 3634, 3345, 2947, 2866, 2843, 2362, 2341, 2164, 2073, 2032, 1701, 1652, 1620, 1563, 1546, 1524, 1496, 1439, 1420, 1345, 1317, 1277, 1247, 1180, 1096, 1055, 1030, 1015, 836, 684, 671, 654, 551 and 503 cm^−1^; UV/vis (MeOH) λ: 266 nm; ^1^H NMR (400 MHz, DMSO): δ 12.23 (s, 1H, -COOH), 8.76 (s, 1H, H-2), 7.99 (d, *J* = 8.9 Hz, 1H, H-5), 7.38 (d, *J* = 15.9 Hz, 1H, H-2′), 7.15 (d, *J* = 2.3 Hz, 1H, H-8), 7.11–7.06 (m, 2H, H-7 and H-1′), 3.89 (s, 3H, -OCH_3_); ^13^C NMR (101 MHz, DMSO): δ 174.55 (C-4 C=O), 167.80 (C-3′ C=O), 164.01 (C-7), 159.29 (C-2), 156.94 (C-8a), 135.96 (C-2′), 126.86 (C-5), 121.20 (C-1′), 118.01 (C-3), 117.25 (C-4a), 115.22 (C-6), 100.85 (C-8), 56.20 (OCH_3_); HRMS (ESI-TOF) (*m*/*z*) C_13_H_10_O_5_ calculated, 247.0606, found 247.082 [M + H]^+^; calculated, 269.043, found, 269.065 [M + Na]^+^; and calculated, 285.017, found, 285.041 [M + K]^+^ [49].

#### 3.3.5. General Procedure for the Synthesis of Conjugates **5a**–**d**

A mixture of 3,5-dimethylisoxazole-4-carboxylic acid (1.0 g, 7 mmol), EDCI (2 g, 10.6 mmol), and DMF (10 mL) was stirred at 0 °C. After 30 min, DMAP (catalytic) was added, and the reaction was stirred for another 10 min. Then, 3-(hydroxymethyl)-4*H*-1-benzopyran-4-one (**3a**, 0.62 g, 3.5 mmol) or methoxy substituted 3-(hydroxymethyl)-4*H*-1-benzopyran-4-one (**3b**/**3c**/**3d**, 0.73 g, 3.5 mmol) was added, and the reaction was stirred overnight. TLC [ethyl acetate–petroleum ether 50:50] was used to monitor the reaction to completion. After completion of the reaction, the solvent was evaporated in a vacuum, and the resulting precipitate was dissolved in ethyl acetate and washed 3 times with water. The solvent was dried over MgSO_4_ and evaporated, and the residue was purified via column chromatography to afford **5a**–**5d** as white to off-white solids.

*(4-Oxo-4H-1-benzopyran-3-yl)methyl 3,5-dimethylisoxazole-4-carboxylate* (**5a**). Yield = 71%; *R*_f_ = 0.79 [ethyl acetate–petroleum ether 50:50]; IR (neat) ν_max_: 2954, 2923, 2867, 2384, 2349, 2298, 2249, 2215, 2167, 2147, 2032, 1995, 1976, 1961, 1900, 1715, 1652, 1610, 1575, 1500, 1464, 1425, 1407, 1381, 1348, 1299, 1253, 1217, 1190, 1168, 1146, 1104, 1055, 1032, 1013, 953, 914, 874, 849, 802, 760, 705, 689, 658 and 627 cm^−1^; UV/vis (MeOH) λ: 293, 245 nm; ^1^H NMR (400 MHz, CDCl_3_): δ 8.25 (dd, *J* = 8.0, 1.5 Hz, 1H, H-5), 8.15 (s, 1H, H-2), 7.70 (ddd, *J* = 8.7, 7.1, 1.7 Hz, 1H, H-7), 7.53–7.36 (m, 2H, H-6 and H-8), 5.22 (s, 2H. –COOC*H_2_*), 2.63 (s, 3H, -CH_3_), 2.41 (s, 3H, -CH_3_); ^13^C NMR (101 MHz, CDCl_3_): δ 176.69 (C-4 C=O), 175.75 (C-5′), 162.56 (C-3′ C=O), 160.10 (C-3′′), 156.61 (C-8a), 156.29 (C-2), 134.19 (C-7), 126.11 (C-5), 125.73 (C-6), 124.27 (C-4a), 119.45 (C-3), 118.41 (C-8), 108.65 (C-4′), 58.36 (C-1′), 13.59 (CH_3_), 11.92 (CH_3_); HRMS (ESI-TOF) (*m*/*z*) C_16_H_13_NO_5_ calculated, 322.0691, found 322.0622 [M + Na]^+^; and calculated, 338.0431, found, 338.0361 [M + K]^+^.

*(5-Methoxy-4-oxo-4H-1-benzopyran-3-yl)methyl 3,5-dimethylisoxazole-4-carboxylate* (**5b**). Yield = 25%; *R*_f_ = 0.32 [ethyl acetate–petroleum ether 50:50]; IR (neat) ν_max_: 3697, 3679, 3656, 3635, 2923, 2867, 2845, 2362, 2342, 2165, 2075, 2027, 1717, 1656, 1607, 1574, 1546, 1524, 1476, 1459, 1438, 1408, 1377, 1357, 1298, 1273, 1240, 1186, 1107, 1055, 1032, 1014, 922, 850, 810, 778, 742 and 701 cm^−1^; UV/vis (MeOH) λ: 314, 254, 224 nm; ^1^H NMR (400 MHz, CDCl_3_): δ 8.00 (s, 1H, H-2), 7.56 (t, *J* = 8.4 Hz, 1H, H-7), 7.02 (dd, *J* = 8.5, 0.8 Hz, 1H, H-8), 6.83 (d, *J* = 8.3 Hz, 1H, H-6), 5.18 (s, 2H, -OCH_2_-), 3.98 (s, 3H, -OCH_3_), 2.64 (s, 3H, -CH_3_), 2.42 (s, 3H, -CH_3_); ^13^C NMR (101 MHz, CDCl_3_): δ 176.20 (C-4 C=O), 175.71 (C-5′′), 162.64 (C-3′), 160.23, 160.14 (C-5 and C-8a), 158.64 (C-3′′), 154.36 (C-2), 134.26 (C-7), 120.49 (C-3), 114.92 (C-4a), 110.31 (C-8), 108.68 (C-4′′), 106.84 (C-6), 58.36 (C-1′), 56.62 (OCH_3_), 13.66 (CH_3_), 11.99 (CH_3_); HRMS (ESI-TOF) (*m*/*z*) C_17_H_15_NO_6_ calculated, 329.0899, found 329.0978 [M]^+^; calculated, 352.0797, found, 352.0741 [M + Na]^+^; and calculated, 368.4063, found, 368.0479 [M + K]^+^.

*(6-Methoxy-4-oxo-4H-1-benzopyran-3-yl)methyl 3,5-dimethylisoxazole-4-carboxylate* (**5c**). Yield = 32%; *R*_f_ = 0.82 [ethyl acetate–petroleum ether 50:50]; IR (neat) ν_max_: 3712, 3696, 3679, 3656, 3635, 2923, 2867, 2844, 2362, 2342, 2164, 2073, 2030, 1978, 1714, 1643, 1610, 1547, 1525, 1484, 1457, 1437, 1403, 1371, 1345, 1320, 1300, 1249, 1203, 1173, 1152, 1107, 1055, 1017, 900, 866, 840, 796, 774, 734, 702 and 627 cm^−1^; UV/vis (MeOH) λ: 320, 248 nm; ^1^H NMR (400 MHz, CDCl_3_): δ 8.14 (s, 1H, H-2), 7.60 (d, *J* = 3.1 Hz, 1H, H-5), 7.41 (d, *J* = 9.2 Hz, 1H, H-8), 7.28 (dd, *J* = 9.2, 3.1 Hz, 1H, H-7), 5.23 (s, 2H, -OCH_2_-), 3.90 (s, 3H, -OCH_3_), 2.64 (s, 3H, -CH_3_), 2.42 (s, 3H, -CH_3_); ^13^C NMR (101 MHz, CDCl_3_): δ 176.57 (C-4 C=O), 175.74 (C-5′′), 162.59 (C-3′ C=O), 160.11 (C-3′′), 157.38 (C-6), 156.07 (C-2), 151.48 (C-8a), 124.89 (C-4a), 124.29 (C-7), 119.83 (C-8), 118.59 (C-3), 108.68 (C-4′′), 105.08 (C-5), 58.45 (C-1′), 56.11 (OCH_3_), 13.59 (CH_3_), 11.93 (CH_3_); HRMS (ESI-TOF) (*m*/*z*) C_17_H_15_NO_6_ calculated, 330.0978, found, 330.0932 [M + H]^+^; calculated, 352.0797, found, 352.0744 [M + Na]^+^; and calculated, 368.4063, found, 368.0484 [M + K]^+^.

*(7-Methoxy-4-oxo-4H-1-benzopyran-3-yl)methyl 3,5-dimethylisoxazole-4-carboxylate* (**5d**). Yield = 63%; *R*_f_ = 0.77 [ethyl acetate–petroleum ether 50:50]; IR (neat) ν_max_: 3711, 3696, 3679, 3655, 3634, 2970, 2923, 2866, 2844, 2826, 2362, 2341, 2165, 2073, 2031, 1979, 1562, 1545, 1524, 1511, 1495, 1477, 1457, 1345, 1322, 1055, 1032 and 1013 cm^−1^; UV/vis (MeOH) λ: 295, 248, 240 nm; ^1^H NMR (400 MHz, CDCl_3_): δ 8.15 (d, *J* = 8.9 Hz, 1H, H-5), 8.07 (s, 1H, H-2), 7.00 (dd, *J* = 8.9, 2.4 Hz, 1H, H-6), 6.85 (d, *J* = 2.4 Hz, 1H, H-8), 5.21 (s, 2H, -OCH_2_-), 3.91 (s, 3H, -OCH_3_), 2.63 (s, 3H, -CH_3_), 2.42 (s, 3H, -CH_3_); ^13^C NMR (101 MHz, CDCl_3_): δ 176.00 (C-4 C=O), 175.74 (C-5′′), 164.50 (C-7), 162.60 (C-3′ C=O), 160.13 (C-3′′), 158.42 (C-8a), 155.81 (C-2), 127.51 (C-5), 119.32 (C-3), 118.15 (C-4a), 115.01 (C-6), 108.70 (C-4′′), 100.58 (C-8), 58.37 (C-1′), 56.03 (OCH_3_), 13.60 (CH_3_), 11.94 (CH_3_); HRMS (ESI-TOF) (*m*/*z*) C_17_H_15_NO_6_ calculated, 330.0978, found 330.0929 [M + H]^+^; calculated, 352.0797, found, 352.0742 [M + Na]^+^; and calculated, 368.4063, found, 368.0480 [M + K]^+^.

#### 3.3.6. General Procedure for the Synthesis of Conjugates **6a**, **6b**, and **6d**

Conjugates **6a**, **6b**, and **6d** were prepared by the general procedure adopted from the literature report [50]. A mixture of 4-oxo-4*H*-1-benzopyran-3-yl-carboxaldehyde (**2a**, 0.5 g, 2.9 mmol) or methoxy-substituted 4-oxo-4*H*-1-benzopyran-3-yl-carboxaldehyde (**2b**/**2c**/**2d**, 0.58 g, 2.9 mmol), (3,5-Dimethyl-isoxazol-4-yl)-methanol (0.80 g, 6.3 mmol), InF_3_ (24 mg, 1.43 mmol, 5 mol%) and Toluene (5 mL) was taken in a round bottom flask fitted with a reflux condenser. The mixture was refluxed for 5 h (125 °C). After completion of the reaction, the reaction mixture was filtered to remove the catalyst and washed with toluene. The solvent was then evaporated in a vacuum, and the resulting precipitate was purified via column chromatography on basic alumina to afford **6a**, **6b**, and **6d** as white solids. Conjugate **6c** was not obtained during the reaction as Starting Material **2c** was left unreacted.

*3-(Bis((3,5-dimethylisoxazol-4-yl)methoxy)methyl)-4H-1-benzopyran-4-one* (**6a**). Yield = 13%; *R*_f_ = 0.48 [ethyl acetate–petroleum ether 50:50]; IR (neat) ν_max_: 2925, 1650, 1611, 1574, 1465, 1425, 1386, 1344, 1312, 1265, 1217, 1178, 1146, 1079, 1026, 883, 848, 818, 761, 741, 698, 674 and 640 cm^−1^; UV/vis (MeOH) λ: 303, 297, 227 nm; ^1^H NMR (400 MHz, CDCl_3_): δ 8.23 (dd, *J* = 8.0, 1.5 Hz, 1H, H-5), 8.13 (s, 1H, H-2), 7.70 (ddd, *J* = 8.7, 7.1, 1.7 Hz, 1H, H-7), 7.59–7.36 (m, 2H, H-6 and H-8), 5.83 (s, 1H, H-1′), 4.43 (dd, *J* = 35.3, 11.9 Hz, 2H, H-6′′), 2.36 (s, 6H, 2xCH_3_), 2.26 (s, 6H, 2xCH_3_); ^13^C NMR (101 MHz, CDCl_3_): δ 176.12 (C-4 C=O), 167.58 (C-5′′), 159.86 (C-3′′), 156.40 (C-8a), 154.93 (C-2), 134.23 (C-7), 126.01 (C-6), 125.78 (C-5), 124.23 (C-4a), 121.56 (C-3), 118.38 (C-8), 110.78 (C-4′′), 95.73 (C-1), 58.19 (C-6′′), 11.19 (CH_3_), 10.28 (CH_3_); HRMS (ESI-TOF) (*m*/*z*) C_22_H_22_N_2_O_6_ calculated, 433.1376, found 433.1302 [M + Na]^+^; and calculated, 449.1115, found, 449.1039 [M + K]^+^.

*3-(Bis((3,5-dimethylisoxazol-4-yl)methoxy)methyl)-5-methoxy-4H-1-benzopyran-4-one* (**6b**). Yield = 11%; *R*_f_ = 0.12 [ethyl acetate–petroleum ether 50:50]; IR (neat) ν_max_: 3708, 3679, 2969, 2923, 2866, 2844, 2827, 2350, 2322, 2165, 2073, 2029, 1976, 1653, 1606, 1572, 1540, 1475, 1456, 1432, 1375, 1346, 1324, 1293, 1270, 1211, 1162, 1054, 1032, 101, 886, 803, 776, 736, 700 and 677 cm^−1^; UV/vis (MeOH) λ: 315, 255, 225 nm; ^1^H NMR (400 MHz, CDCl_3_): δ 7.96 (s, 1H, H-2), 7.56 (t, *J* = 8.4 Hz, 1H, H-7), 7.02 (dd, *J* = 8.5, 0.8 Hz, 1H, H-8), 6.83 (d, *J* = 8.3 Hz, 1H, H-6), 5.78 (s, 1H, H-1′), 4.41 (dd, *J* = 43.3, 11.9 Hz, 4H, H-6′′), 3.98 (s, 3H, -OCH_3_), 2.36 (s, 6H, 2X –CH_3_), 2.25 (s, 6H, 2X –CH_3_); ^13^C NMR (101 MHz, CDCl_3_): δ 175.86 (C-4 C=O), 167.52 (C-5′′), 160.20 (C-5), 159.87 (C-3′′), 158.39 (C-8a), 152.99 (C-2), 134.26 (C-7), 122.94 (C-3), 114.86 (C-4a), 110.92 (C-4′′), 110.31 (C-6), 106.89 (C-8), 96.03 (C-1′), 58.58 (C-6′′), 56.64 (OCH_3_), 11.21 (CH_3_), 10.29 (CH_3_); HRMS (ESI-TOF) (*m*/*z*) C_23_H_24_N_2_O_7_ calculated, 441.1662, found 441.1602 [M + H]^+^; calculated, 463.1481, found, 463.1481 [M + Na]^+^; and calculated, 479.1221, found, 479.1156 [M + K]^+^.

*3-(Bis((3,5-dimethylisoxazol-4-yl)methoxy)methyl)-7-methoxy-4H-1-benzopyran-4-one* (**6d**). Yield = 10%; *R*_f_ = 0.42 [ethyl acetate–petroleum ether 50:50]; IR (kbr) ν_max_: 3711, 3696, 3678, 3655, 2970, 2923, 2866, 2844, 2826, 2362, 2341, 2165, 2074, 2028, 1978, 1544, 1524, 1511, 1476, 1457, 1345, 1323, 1054, 1032 and 1013 cm^−1^; UV/vis (MeOH) λ: 295, 248, 240, 223 nm; ^1^H NMR (400 MHz, CDCl_3_): δ 8.12 (d, *J* = 8.9 Hz, 1H, H-5), 8.04 (d, *J* = 0.7 Hz, 1H, H-2), 6.99 (dd, *J* = 8.9, 2.4 Hz, 1H, H-6), 6.84 (d, *J* = 2.4 Hz, 1H, H-8), 5.82 (d, *J* = 0.7 Hz, 1H, H-1′), 4.42 (dd, *J* = 37.6, 11.9 Hz, 4H, H-6′′), 3.90 (s, 3H, -OCH_3_), 2.35 (s, 6H, 2X-CH_3_), 2.26 (s, 6H, 2X -CH_3_); ^13^C NMR (101 MHz, CDCl_3_): δ 175.45 (C-4 C=O), 167.56 (C-7), 164.50 (C-5′′), 159.87 (C-3′′), 158.19 (C-8a), 154.45 (C-2), 127.36 (C-5), 121.41 (C-3), 118.05 (C-4a), 115.08 (C-6), 110.83 (C-4′′), 100.50 (C-1′), 95.82 (C-8), 58.18 (C-6′′), 56.02 (OCH_3_), 11.18 (CH_3_), 10.26 (CH_3_); HRMS (ESI-TOF) (*m*/*z*) C_23_H_24_N_2_O_7_ calculated, 441.1662, found 441.1594 [M + H]^+^; calculated, 463.1481, found, 463.1410 [M + Na]^+^; and calculated, 479.1221, found, 479.1145 [M + K]^+^.

#### 3.3.7. General Procedure for the Synthesis of Conjugates 7**a**, 7**c**, and 7**d**

Conjugates **7a**, **7c**, and **7d** were prepared via the general procedure adopted from the literature report [51]. A mixture of 4-oxo-4*H*-1-benzopyran-3-yl- carboxaldehyde (**2a**, 0.5 g, 2.9 mmol) or methoxy substituted 4-oxo-4*H*-1-benzopyran-3-yl- carboxaldehyde (**2b**/**2c**/**2d**, 0.59 g, 2.9 mmol), NBS (0.61 g, 3.44 mmol), and AIBN (20 mg) in CCl_4_ (25 mL) was taken in a round bottom flask and refluxed for 1 h. The mixture was then cooled in ice-water bath, followed by the addition of 3-amino-5-methyl isoxazole (0.56 g, 5.74 mmol). After stirring the reaction mixture for 30 min at room temperature, the mixture was diluted with CH_2_Cl_2_ (100 mL), washed with water, dried with MgSO_4_, and the solvent evaporated. The resulting precipitate was purified by column chromatography to afford **7a**, **7c**, and **7d** as solid products. For Conjugate **7b**, Starting Material **2b** reacted and gave a very complicated reaction as indicated by TLC, and Product **7b** could not be isolated in pure form.

*N-(5-Methylisoxazol-3-yl)-4-oxo-4H-1-benzopyran-3-carboxamide* (**7a**). Yield = 31%; *R*_f_ = 0.69 [ethyl acetate–petroleum ether 50:50]; IR (neat) ν_max_: 3207, 3146, 3074, 2956, 2922, 2869, 1689, 1634, 1614, 1567, 1548, 1465, 1436, 1397, 1377, 1346, 1313, 1268, 1252, 1206, 1177, 1142, 1123, 1104, 1054, 1033, 1013, 939, 912, 887, 862, 832, 806, 764, 718 and 644 cm^−1^; UV/vis (MeOH) λ: 295, 242 nm; ^1^H NMR (400 MHz, CDCl_3_): δ 11.80 (s, 1H, NH), 9.03 (s, 1H, H-2), 8.35 (dd, *J* = 8.0, 1.7 Hz, 1H, H-5), 7.80 (ddd, *J* = 8.7, 7.1, 1.7 Hz, 1H, H-7), 7.67–7.45 (m, 2H, H-6 and H-8), 6.75 (s, 1H, CH), 2.43 (s, 3H, CH_3_); ^13^C NMR (101 MHz, CDCl_3_): δ 176.96 (C-4 C=O), 169.95 (C-1′ C=O), 163.24 (C-2), 161.10 (C-5), 157.71 (C-3′′), 156.24 (C-8a), 135.21 (C-7), 126.86, 126.64 (C6 and C-5), 124.16 (C-4a), 118.61 (C-8), 115.28 (C-3), 97.16 (C-4′′), 12.84 (CH_3_); HRMS (ESI-TOF) (*m*/*z*) C_14_H_10_N_2_O_4_ calculated, 271.0719, found 271.0676 [M + H]^+^; calculated, 293.0538, found, 293.0490 [M + Na]^+^; and calculated, 309.0279, found, 309.0230 [M + K]^+^.

*6-Methoxy-N-(5-methylisoxazol-3-yl)-4-oxo-4H-1-benzopyran-3-carboxamide* (**7c**). Yield = 25%; *R*_f_ = 0.76 [ethyl acetate–petroleum ether 50:50]; IR (neat) ν_max_: 3697, 3679, 3662, 3573, 2969, 2922, 2866, 2844, 2826, 2361, 2341, 2165, 2073, 2050, 1977, 1690, 1611, 1560, 1546, 1512, 1478, 1457, 1434, 1374, 1346, 1307, 1262, 1160, 1142, 1054, 1032, 1014 and 722 cm^−1^; UV/vis (MeOH) λ: 326, 237 nm; ^1^H NMR (400 MHz, CDCl_3_): δ 11.84 (s, 1H, -NH-), 9.00 (s, 1H, H-2), 7.66 (d, *J* = 3.0 Hz, 1H, H-5), 7.52 (d, *J* = 9.2 Hz, 1H, H-8), 7.36 (dd, *J* = 9.2, 3.1 Hz, 1H, H-7), 6.75 (s, 1H, CH), 3.94 (s, 3H, -OCH_3_), 2.43 (s, 3H, -CH_3_); ^13^C NMR (101 MHz, CDCl_3_): δ 176.75 (C-4 C=O), 169.95 (C-5′′), 162.82 (C-2), 161.31 (C-1′), 158.18 (C-6), 157.77 (C-3′′), 151.14 (C-8a), 125.23 (C-7), 124.96 (C-4a), 120.01 (C-8), 114.52 (C-3), 105.55 (C-5), 97.17 (C-4′′), 56.24 (OCH_3_), 12.82 (CH_3_); HRMS (ESI-TOF) (*m*/*z*) C_15_H_12_N_2_O_5_ calculated, 323.0644, found 323.0599 [M + Na]^+^; and calculated, 339.0383, found, 339.0330 [M + K]^+^.

*7-Methoxy-N-(5-methylisoxazol-3-yl)-4-oxo-4H-1-benzopyran-3-carboxamide* (**7d**). Yield = 73%; *R*_f_ = 0.65 [ethyl acetate–petroleum ether 50:50]; IR (neat) ν_max_: 3712, 3696, 3678, 3655, 2922, 2867, 2846, 2362, 2341, 2164, 2031, 1690, 1652, 1615, 1560, 1547, 1508, 1437, 1377, 1304, 1277, 1204, 1154, 1092, 1055, 1031, 1015, 940, 889, 849, 794, 763, 735, 671, 638, 610, 564 and 505 cm^−1^; UV/vis (MeOH) λ: 293, 255, 247, 227 nm; ^1^H NMR (400 MHz, CDCl_3_): δ 11.91 (s, 1H, -NH-), 8.94 (s, 1H, H-2), 8.23 (d, *J* = 9.0 Hz, 1H, H-5), 7.09 (dd, *J* = 9.0, 2.4 Hz, 1H, H-6), 6.95 (d, *J* = 2.4 Hz, 1H, H-8), 6.73 (d, *J* = 0.5 Hz, 1H, -CH-), 3.94 (s, 3H, -OCH_3_), 2.43 (s, 3H, -CH_3_); ^13^C NMR (101 MHz, CDCl_3_): δ 176.23 (C-4 C=O), 169.90 (C-5″), 165.28 (C-7), 162.75 (C-2), 161.33 (C-1′), 158.17, 157.76 (C-8a and C-3″), 127.99 (C-5), 117.79 (C-4a), 116.14 (C-6), 115.16 (C-3), 100.77 (C-8), 97.17 (C-4″), 56.23 (OCH_3_), 12.82 (CH_3_); HRMS (ESI-TOF) (*m*/*z*) C_15_H_12_N_2_O_5_ calculated, 301.0824, found 301.0776 [M + H]^+^; calculated, 323.0644, found, 323.0591 [M + Na]^+^; and calculated, 339.0383, found, 339.0330 [M + K]^+^.

#### 3.3.8. Synthesis of Conjugate **8a**

Conjugate **8a** was prepared via the general procedure adopted from the literature report [51]. A mixture of 4-oxo-4*H*-1-benzopyran-3-yl- carboxaldehyde (**2a**, 0.5 g, 2.9 mmol), NBS (0.61 g, 3.44 mmol), and AIBN (20 mg) in CCl_4_ (25 mL) was taken in a round bottom flask and refluxed for 1 h. The mixture was then cooled in an ice-water bath, followed by the addition of (3,5-dimethyl-isoxazol-4-yl)-methanol (0.73 g, 5.74 mmol). After stirring the reaction mixture for 30 min at room temperature, the mixture was diluted with CH_2_Cl_2_ (100 mL), washed with water, dried with MgSO_4_, and the solvent evaporated. The resulting precipitate was purified by column chromatography to afford **8a** as white solid. For the Conjugates **8b**, **8c**, and **8d**, Starting Materials **2b**, **2c**, and **2d** reacted and gave very complicated reactions, as indicated by TLC′s, and Products **8b**, **8c**, and **8d** could not be isolated in pure form.

*(3,5-Dimethylisoxazol-4-yl)methyl 4-oxo-4H-1-benzopyran-3-carboxylate* (**8a**). Yield = 29%; *R*_f_ = 0.5 [ethyl acetate–petroleum ether 50:50]; IR (neat) ν_max_: 3055, 2957, 2923, 2867, 2350, 2082, 1705, 1655, 1614, 1569, 1508, 1463, 1426, 1383, 1339, 1311, 1291, 1263, 1201, 1151, 1115, 1103, 1085, 1056, 1033, 971, 957, 938, 900, 873, 855, 813, 791, 771, 750, 739, 686, 675 and 634 cm^−1^; UV/vis (MeOH) λ: 293, 225 nm; ^1^H NMR (400 MHz, CDCl_3_): δ 8.62 (s, 1H, H-2), 8.26 (dd, *J* = 8.0, 1.5 Hz, 1H, H-5), 7.70 (ddd, *J* = 8.7, 7.1, 1.7 Hz, 1H, H-7), 7.50–7.44 (m, 2H, H-6 and H-8), 5.13 (s, 2H, -OCH_2_), 2.48 (s, 3H, CH_3_), 2.37 (s, 3H, CH_3_); ^13^C NMR (101 MHz, CDCl_3_): δ 173.29 (C-4 C=O), 168.80 (C-1′ C=O), 163.31 (C-3′′), 162.12 (C-2), 159.97 (C-5′′), 155.69 (C-8a), 134.45 (C-7), 126.69, 126.52 (C-6 and C-5), 125.20 (C-4a), 118.30 (C-8), 115.97 (C-3), 109.67 (C-4′′), 56.07 (C-6′′ OCH_2_), 11.34 (CH_3_), 10.26 (CH_3_); HRMS (ESI-TOF) (*m*/*z*) C_16_H_13_NO_5_ calculated, 300.0872, found 300.0825 [M + H]^+^; calculated, 322.0691, found, 322.0640 [M + Na]^+^; and calculated, 338.0431, found, 338.0378 [M + K]^+^.

#### 3.3.9. General Procedure for the Synthesis of Conjugates **9a**, **9c**, and **9d**

A mixture of (*E*)-3-(4-oxo-4*H*-1-benzopyran-3-yl) acrylic acid (**4a**, 0.5 g, 2.3 mmol) or methoxy substituted (*E*)-3-(4-oxo-4*H*-1-benzopyran-3-yl) acrylic acid (**4b**/**4c**/**4d**, 0.57 g, 2.3 mmol), EDCI (1.33 g, 6.9 mmol) and DMF (10 mL) were stirred at 0 °C. After 30 min, a catalytic amount of DMAP was added and stirred for an additional 10 min. Then, (3,5-dimethyl-isoxazol-4-yl)-methanol (0.35 g, 2.8 mmol) was added, and the reaction was stirred overnight. After completion of the reaction, the solvent was evaporated under vacuum, and the resulting precipitate was dissolved in ethyl acetate and washed 3 times with water. The solvent was dried over MgSO_4_, evaporated, and the residue was purified via column chromatography to afford **9a**, **9c**, and **9d** as solid products.

*(3,5-Dimethylisoxazol-4-yl)methyl€-3-(4-oxo-4H-1-benzopyran-3-yl)acrylate* (**9a**). Yield = 2%; *R*_f_ = 0.71 [ethyl acetate–petroleum ether 50:50]; IR (neat) ν_max_: 3698, 3679, 3656, 2954, 2922, 2867, 2846, 2362, 2341, 1713, 1658, 1616, 1564, 1464, 1406, 1376, 1356, 1309, 1290, 1265, 1245, 1216, 1155, 1054, 1032, 1013, 857, 762 and 687 cm^−1^; UV/vis (MeOH) λ: 290, 267 nm; ^1^H NMR (400 MHz, CDCl_3_): δ 8.27 (dd, *J* = 8.0, 1.6 Hz, 1H, H-5), 8.11 (s, 1H, H-2), 7.70 (ddd, *J* = 8.6, 7.1, 1.7 Hz, 1H, H-7), 7.50–7.43 (m, 2H, H-6 and H-8), 7.35 (q, *J* = 15.8 Hz, 2H, H-1′ and H-2′), 5.01 (s, 2H, -*C*H_2_-OCO-), 2.45 (s, 3H, CH_3_), 2.31 (s, 3H, CH_3_); ^13^C NMR (101 MHz, CDCl_3_): δ 176.04 (C-4 C=O), 168.52 (C-3′ C=O), 167.24 (C-3″), 159.96 (C-5″), 157.91 (C-2), 155.64 (C-8a), 136.45 (C-1′), 134.26 (C-7), 126.47, 126.10 (C-5 and C-6), 124.32 (C-4a), 121.56 (C-8), 119.26 (C-3), 118.27 (C-2′), 110.04 (C-4″), 55.30 (C-6″ OCH_2_), 11.28 (CH_3_), 10.21 (CH_3_); HRMS (ESI-TOF) (*m*/*z*) C_18_H_15_NO_5_ calculated, 326.1028, found 326.0973 [M + H]^+^; calculated, 348.0848, found, 348.0788 [M + Na]^+^; and calculated, 364.0587, found, 364.0527 [M + K]^+^.

*(3,5-Dimethylisoxazol-4-yl)met€(E)-3-(6-methoxy-4-oxo-4H-1-benzopyran-3-yl)acrylate* (**9c**). Yield = 8%; *R*_f_ = 0.78 [ethyl acetate–petroleum ether 50:50]; IR (neat) ν_max_: 3712, 3696, 3679, 3655, 3634, 3618, 3593, 3571, 3379, 2968, 2923, 2867, 2845, 2362, 2342, 2163, 2072, 2030, 1978, 1711, 1656, 1609, 1566, 1546, 1525, 1512, 1483, 1457, 1436, 1401, 1369, 1341, 1287, 1245, 1198, 1165, 1145, 1101, 1055, 1032, 1012, 954, 866, 839, 823, 727, 671 and 603 cm^−1^; UV/vis (MeOH) λ: 244, 225 nm; ^1^H NMR (400 MHz, CDCl_3_): δ 8.10 (s, 1H, H-2), 7.62 (d, *J* = 3.1 Hz, 1H, H-5), 7.41 (dd, *J* = 11.8, 4.7 Hz, 2H, H-1′ and H-2′), 7.33–7.27 (m, 2H, H-7 and H-8), 5.01 (s, 2H, -OCH_2_), 3.91 (s, 3H, -OCH_3_), 2.46 (s, 3H, -CH_3_), 2.32 (s, 3H, -CH_3_); ^13^C NMR (101 MHz, CDCl_3_): δ 175.90 (C-4 C=O), 168.53 (C-3′ C=O), 167.31 (C-3″), 159.99 (C-5″), 157.69 (C-6), 157.67 (C-2), 150.52 (C-8a), 136.68 (C-1′), 125.03 (C-4a), 124.28 (C-2′), 121.30 (C-7), 119.71 (C-8), 118.45 (C-3), 110.08 (C-4″), 105.55 (C-5), 56.13 (OCH_3_), 55.32 (C-6′′ OCH_2_), 11.30 (CH_3_), 10.23 (CH_3_); HRMS (ESI-TOF) (*m*/*z*) C_19_H_17_NO_6_ calculated, 356.1134, found 356.1086 [M + H]^+^; calculated, 378.0954, found, 378.0904 [M + Na]^+^; and calculated, 394.0693, found, 394.0643 [M + K]^+^.

*(3,5-Dimethylisoxazol-4-yl)€hyl(E)-3-(7-methoxy-4-oxo-4H-1-benzopyran-3-yl)acrylate* (**9d**). Yield = 8%; *R*_f_ = 0.68 [ethyl acetate–petroleum ether 50:50]; IR (neat) ν_max_: 3751, 3710, 3696, 3678, 3655, 3634, 2970, 2923, 2866, 2844, 2826, 2362, 2341, 2165, 2073, 2029, 1978, 1544, 1523, 1511, 1495, 1476, 1457, 1345, 1322, 1055, 1032 and 1013 cm^−1^; UV/vis (MeOH) λ: 293, 255, 223 nm; ^1^H NMR (400 MHz, CDCl_3_): δ 8.17 (d, *J* = 8.9 Hz, 1H, H-5), 8.03 (s, 1H, H-2), 7.34 (q, *J* = 15.8 Hz, 2H, H-1′ and H-2′), 7.01 (dd, *J* = 8.9, 2.4 Hz, 1H, H-6), 6.85 (d, *J* = 2.4 Hz, 1H, H-8), 5.00 (s, 2H, -OCH_2_-), 3.91 (s, 3H, -OCH_3_), 2.45 (s, 3H, -CH_3_), 2.31 (s, 3H, -CH_3_); ^13^C NMR (101 MHz, CDCl_3_): δ 175.43 (C-4 C=O), 168.52 (C-3′ C=O), 167.31 (C-3″), 164.55 (C-7), 159.99 (C-5″), 157.49 (C-2), 157.43 (C-8a), 136.62 (C-1′), 127.88 (C-5), 121.41 (C-2′), 119.22 (C-3), 118.18 (C-4a), 115.30 (C-6), 110.08 (C-4″), 100.52 (C-8), 56.08 (OCH_3_), 55.28 (C-6″ OCH_2_), 11.29 (CH_3_), 10.21 (CH_3_); HRMS (ESI-TOF) (*m*/*z*) C_19_H_17_NO_6_ calculated, 356.1134, found 356.1084 [M + H]^+^; calculated, 378.0954, found, 378.0899 [M + Na]^+^; and calculated, 394.0693, found, 394.0636 [M + K]^+^.

### 3.4. Cytotoxicity Assay of Conjugates

Cell viability assays were performed using the MTS protocol. The in vitro cytotoxicity of the conjugates was evaluated using six human cancer cell lines, one human normal kidney cell line, and one mammalian normal kidney cell line, which included human leukemia carcinoma cell lines (CCRF-CEM), human ovarian adenocarcinoma cell lines (SKOV-3), human breast tumor cell lines (MDA-MB-231), human prostate cancer cell lines (PC-3), androgen-independent human prostate cancer cell lines (DU-145), human renal carcinoma (iSLK), human embryonic kidney cell lines (HEK-293), and normal mammalian kidney cell line (LLCPK) to determine the toxicity of the synthesized conjugates. CCRF-CEM cells were seeded at 50,000 cells in 0.1 mL per well in 96-well plates, and in the case of SKOV-3, MDA-MB-231, PC-3, DU-145, HEK-293, and LLCPK cell lines, the cells were seeded at 5000 cells in 0.1 mL per well in 96-well plates. The CCRF-CEM cells were seeded in RPMI-1640 media containing 2mM L-glutamine, 1.5 g/L sodium bicarbonate, supplemented with fetal bovine serum (FBS) 10% media, SKOV-3, MDA-MB-231, and LLCPK cells were seeded in MEME media with Earle’s salts and sodium bicarbonate, without L-glutamine, supplemented with fetal bovine serum (FBS) 10%, PC-3 cells were seeded in DMEM:F-12 containing 1:1 mixture with L-glutamine with HEPES, supplemented with fetal bovine serum (FBS) 10%, and DU-145 and HEK-293 cells were seeded in DMEM containing 4500 mg/L glucose, L-glutamine, and sodium bicarbonate, without sodium pyruvate, supplemented with FBS (10%), 24 h prior to the experiment. The compounds at a final concentration of 25 µM (higher concentration stock solution prepared in DMSO) were added to each well in triplicate and incubated for 24 and 72 h at 37 °C in a humidified atmosphere (95% air and 5% CO_2_). The cell viability was then determined by measuring the absorbance of the formazan product at 490 nm using a microplate reader using a SpectraMax M2 microplate spectrophotometer, Molecular Devices, San Jose, CA, USA. The percentage of cell survival was calculated as [(OD value of cells treated with the test mixture of compounds) − (OD value of culture medium)]/[(OD value of control cells) − (OD value of culture medium)] × 100%. IC_50_ values of compounds were calculated using non-linear regression analysis in GraphPad Prism (Boston, MA, USA).

### 3.5. In Vitro Screening for Compound ***5a***

Compound **5a** was screened in a single dose (10 μM) against NCI-60 cell panel by Developmental Therapeutics Program, National Cancer Institute, Division of Cancer Treatment and Diagnosis, Bethesda, MD, USA, as reported. The cell lines in the NCI-60 panel include leukemia, non-small lung, colon, CNS, melanoma, ovarian, renal, prostate, and breast cancer cell lines. The data was reported for one dose as the mean of the percent growth of treated cells related to the no-drug control.

### 3.6. Protein Kinase Assay

Compound **5a** was tested at a single dose of 20 μM against 9 kinases, viz., Abl1, c-Kit, c-Src, CDK2/cyclin A1, CSK, EGFR, mTOR/FRAP1, p38a/MAPK14, PKCa, and PI3K using our previously reported protocol [52,53] in duplicate by Reaction Biology Corporation. The respective positive controls, e.g., staurosporine for all the kinases, except SB202190 for P38a/MAP14 and P1-103 for mTOR/FRAP1, were used, and the kinase reaction was performed at 200 μM ATP concentration for all the kinases except for PI3K, which was performed at 10 µM ATP.

### 3.7. Serum Stability

HPLC analysis was carried out to evaluate the proteolytic stability of selected conjugate **5a** in the presence of human serum. First, different concentrations of Compound **5a** were analyzed using the analytical HPLC, and a standard curve was plotted for the different concentrations of Compound **5a** versus the area under the curve. Second, the stability experiment was performed by the following procedure. Biological conditions were mimicked by suspending human serum (250 μL) in RPMI media (700 μL) in Eppendorf tube (1.5 mL). The mixture was equilibrated to 37 ± 1 °C for 15 min, and then 50 μL of conjugate stock solution (1 mM in DMSO) was added to it. As Control 1, one vial was treated with 50 μL of DMSO without Compound **5a**, and as Control 2, another vial was treated with Compound **5a** but without any human serum (instead used 250 μL of deionized water). The initial time was recorded, and 100 μL aliquots of the reaction mixture were removed and added to methanol (200 μL) for precipitation of serum proteins in human serum. The solution turned cloudy, which was cooled down to 4 °C for 15 min. It was then spun at 500× *g* for 15 min to pellet the serum proteins. A 50 μL of the supernatant was injected into an RP-HPLC Vydac C18 column using an autoinjector. A linear gradient from 10% to 100% acetonitrile/water in 65 min with a flow rate of 1.0 mL/min was used, and the absorbance of the eluting peaks was detected at 254 nm. An aliquot of reaction solution (100 μL) was removed after regular time intervals of 2, 5, 10, 20, 40, and 80 min, and the procedure was repeated. The area under the curve was used to calculate the percentage of remaining Compound **5a** at a given time. The relative percentage of Compound **5a** was then plotted on a graph to obtain the stability in the human serum at the particular time intervals of incubation. Then, using the standard curve and relative percentage curve, the half-life of Compound **5a** was calculated.

### 3.8. Cell Apoptosis Assay

The assay was detected using a commercially available kit (FITC Annexin V Apoptosis Detection Kit II, BD Biosciences, San Jose, CA, USA) per the manufacturer’s instructions. Briefly, MDA-MB-231 cells were seeded in 6-well plates at a concentration of 1 × 10^6^ cells/well for 24 h; after that, cells were treated with **5a** (5 µM) for 72 h. Dox (5 µM) was used as a positive control. After the treatment period, cells were washed with cold phosphate-buffered saline (PBS) twice. The cells were collected after trypsin treatment and centrifugated to get a pellet, and a binding buffer (1X) was used to resuspend the cells. Annexin V (5 μL) and propidium iodide (PI) (5 μL) were mixed with 100 μL of the suspended cells (1 × 10^6^ cells/mL), followed by 15 min incubation at room temperature in the dark. The mixture was diluted by adding 400 μL of 1× binding buffer, and the cells were analyzed using flow cytometry, FACS Verse (BD Biosciences, USA). The experiment was repeated three times independently.

## 4. Conclusions

This present study reports the synthesis of 14 new benzopyran-4-one-isoxazole hybrid compounds exploring five different chemical linkers such as ester (formed from 3-(hydroxymethyl) substituted-4*H*-1-benzopyran-4-one), acetal, amide, reverse ester (formed from (3,5-dimethyl-isoxazol-4-yl)-methanol) and acrylic ester for the design strategy. Various spectroscopic analyses and HPLC techniques confirmed the structure and compounds’ purity. Four hybrid compounds (**5a**–**d**) displayed moderate to high antiproliferative activities against CCRF-CEM, SKOV-3, MDA-MB-231, PC-3, DU-145, and iSLK cell lines tested and were in the range of 5.2–22.2 μM against MDA-MB-231 cells, while they were minimally cytotoxic to the human embryonic kidney (HEK-293) and porcine kidney (LLC-PK1) cell lines. The IC_50_ values of conjugates (**5a**–**d**) against normal HEK-293 cells were 293.2, 191.5, 222.1, and 102.4 μM, respectively. The SAR suggests that the hybrid compounds formed via ester linkage from 3-(hydroxymethyl) substituted-4*H*-1-benzopyran-4-one and 3,5-dimethylisoxazole-4-carboxylic acid were the compounds with significant antiproliferative activities and the least cytotoxicity to normal cell lines amongst all other chemical linkages, i.e., acetal, amide, reverse ester, and acrylic ester, that were screened. Compound **5a** showed the highest antiproliferative activity against all the cancer cell lines tested, further indicating that the methoxy substituent on the benzopyran-4-one moiety reduces the antiproliferative activity. Screening of **5a** on NCI 60 cell lines showed good antiproliferative activity against leukemia and breast cancer cell lines at 10 μM. Compound **5a** did not show protein tyrosine kinase inhibitory activity and had a half-life of 13.6 min in human serum. Moreover, it induces apoptosis in MDA-MB-231 cells by 50.8% compared to untreated cells. Further studies will be performed to understand the mechanism of induction of apoptosis by these compounds for developing a newer generation of anticancer compounds.

## Figures and Tables

**Table 1:** IC_50_ (μM) of selected compounds **5a**–**d** against human leukemia carcinoma (CCRF-CEM), human breast cancer (MDA-MB-231), human prostate cancer (PC-3), androgen-independent human prostate cancer (DU-145), and human embryonic kidney (HEK-293) cell lines.

IC_50_^a^ (μM)					
	CCRF-CEM	MDA-MB-231	PC3	DU-145	HEK-293
**5a**	11.55	5.604	17.84	15.28	293.2
**5b**	36.46	22.2	14.77	51.15	191.5
**5c**	3.315	11.03	12.92	12.23	222.1
**5d**	16.16	5.263	12.7	14.58	102.4

**^a^** The average IC_50_ values were calculated from three independent tests performed in triplicate.

**Table 2:** Screening of compound **5a** against protein kinases *.

Protein Kinases	% Enzyme Activity (Relative to DMSO Controls)
ABL1	96.87
c-Kit	95.32
c-Src	113.33
CDK2/cyclin A1	105.34
CSK	93.03
EGFR	99.84
mTOR/FRAP1	111.70
p38a/MAPK14	83.11
PKCa	102.63
PI3K	109.63
(p110a/p65a)	

* Compound **5a** was screened at 20 µM in duplicate. DMSO was used as a negative control.

## Data Availability

The data presented in this study are available in the article and Supplementary Material.

## Supplementary Materials

The following supporting information can be downloaded at: https://www.mdpi.com/article/10.3390/molecules28104220/s1. Figures S1.1–S26.3: ^1^H and ^13^C NMR (including selected DEPT) spectroscopy data; Figures S27.1–S27.4: analytical HPLC analysis; Table S1: HPLC retention time of 5a–d; Figures S28.1–S28.8: cytotoxicity graphs of starting materials and intermediates, Tables S2–S3: antiproliferative activities for Dox and 5a–5d; Figures S29.1–S30: serum stability studies of Compound **5a**; Table S4: serum stability data of Compound **5a**; Page 84: calculation of half-life of **5a** in human serum.

## Sample Availability

Samples of Compounds **5a**–**d**, **6a**–**b**, **6d**, **7a**, **7c**–**d**, **8a**, **9a**, and **9c**–**d** are available from the authors.
