# Supplementary material for: Design, Synthesis, and Antiproliferative Activity of Benzopyran-4-One-Isoxazole Hybrid Compounds
Source: Molecules. 2023 May 21;28(10):4220. doi: 10.3390/molecules28104220 (PMC10224329; doi:10.3390/molecules28104220)

Design, Synthesis, and Antiproliferative Activity of Benzopyran-4-one-Isoxazole Hybrid Compounds

Shilpi Gupta,[a,b] Shang Eun Park,[a] Saghar Mozaffari,[a] Bishoy El-Aarag,[a,c] Keykavous Parang,[a] and Rakesh Kumar Tiwari[a,*]

[a]Center for Targeted Drug Delivery, Department of Biomedical and Pharmaceutical Sciences, Chapman University School of Pharmacy, Harry and Diane Rinker Health Science Campus, Irvine, CA 92618-1908, USA;

[b]Department of Chemistry, Hindu College, Sonipat, Haryana 131001, India;

[c]Biochemistry Division, Chemistry Department, Faculty of Science, Menoufia University, Shebin El-Koom 32512, Egypt

Table of Contents:

^{1}H & ^{13}C NMR &/or DEPT-135 spectra of compounds 2-9	2-65
Analytical HPLC analysis of compounds 5a-5d	66-70
Cytotoxicity data for starting materials and intermediates	71-78
Antiproliferative activity (%) data on treatment with Dox & 5a-5d	79
Anti-proliferative activity (%) data on non-cancerous cell lines	80
Serum stability studies data	81-85

Figure S1.1. ^{1}H NMR spectra of compound 2a

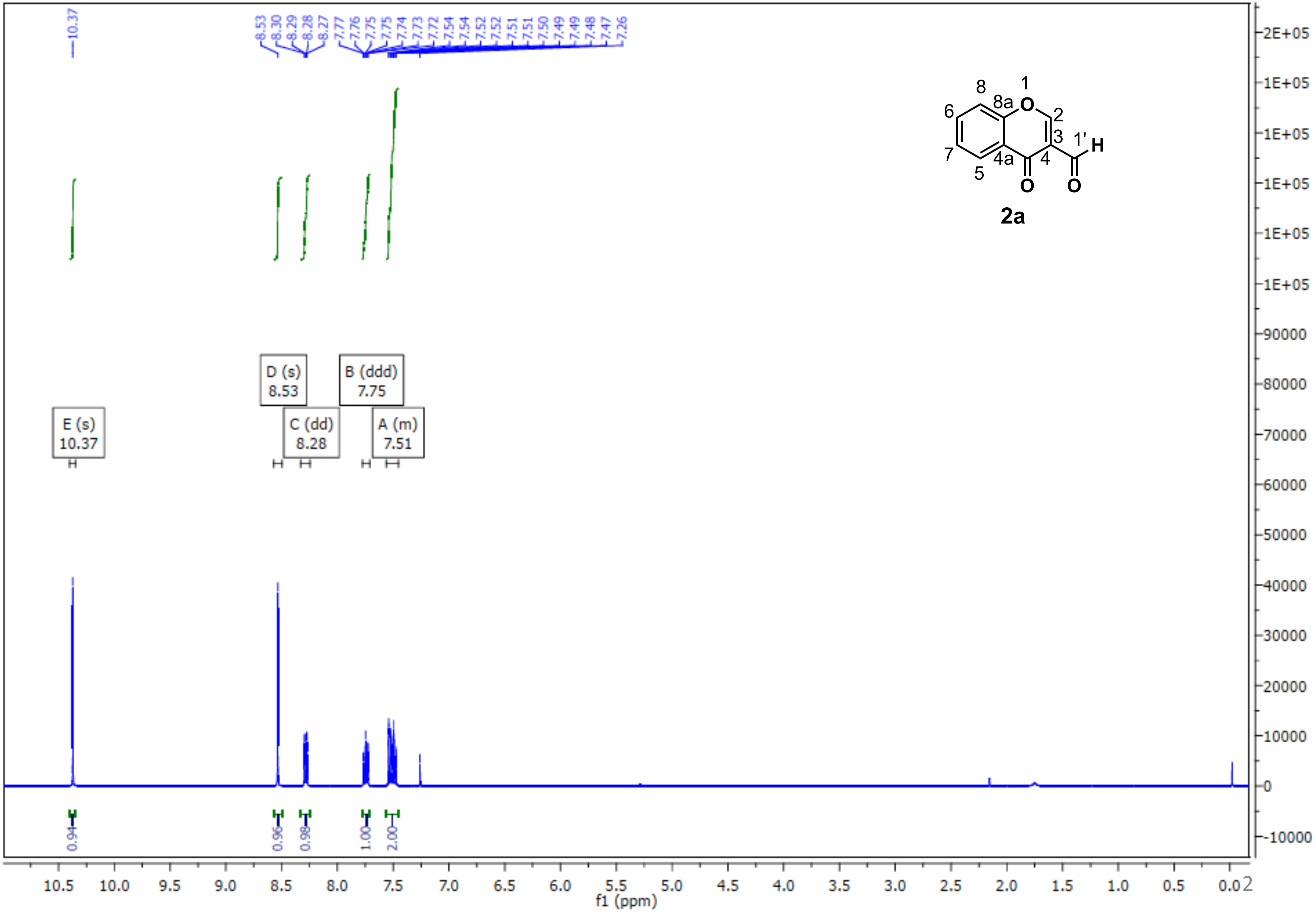

Figure S1.2. ^{13}C NMR spectra of compound 2a

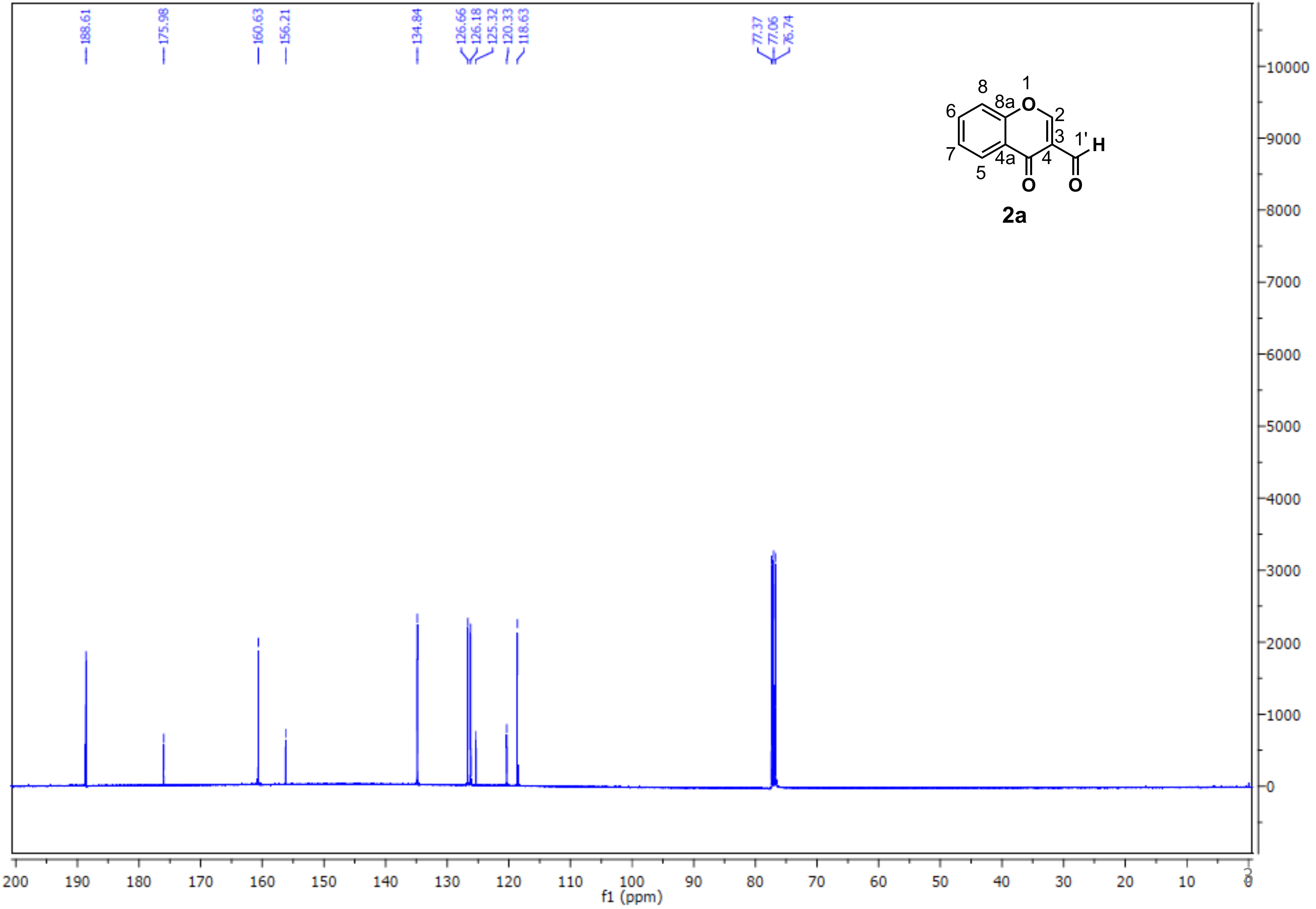

Figure S2.1. ^1H NMR spectra of compound 2b

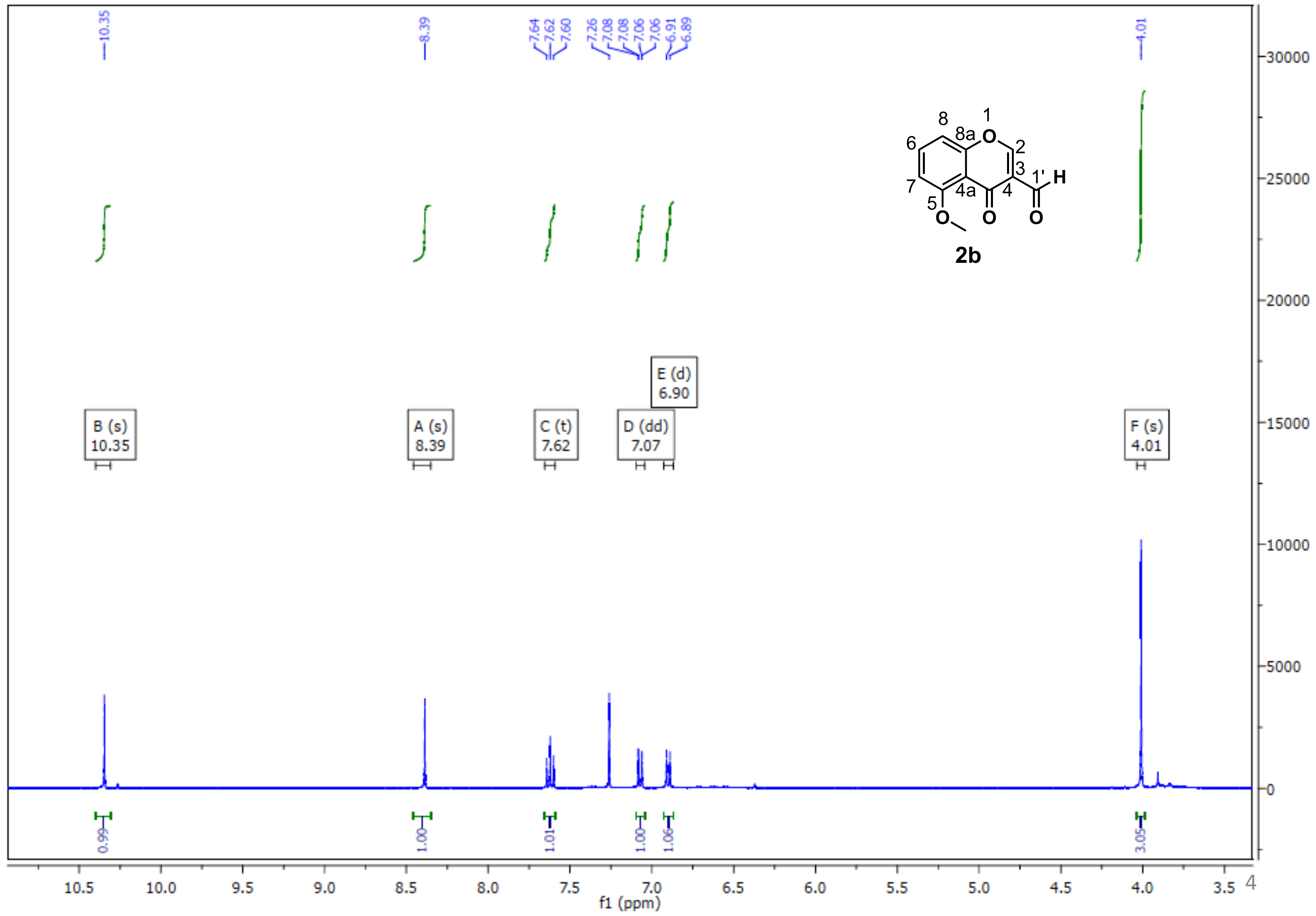

Figure S2.2. ^{13}C NMR spectra of compound 2b

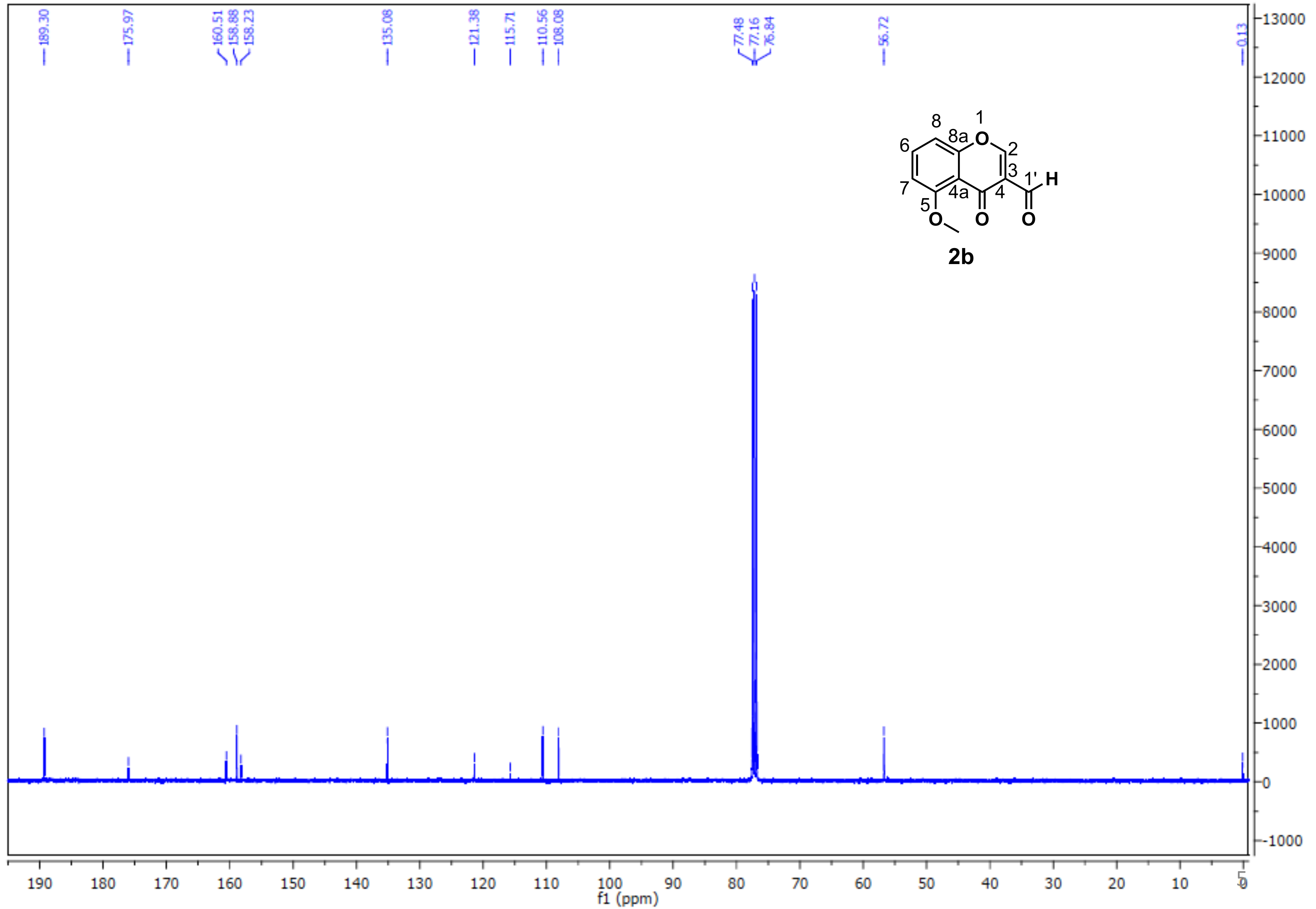

Figure S3.1. ^{1}H NMR spectra of compound 2c

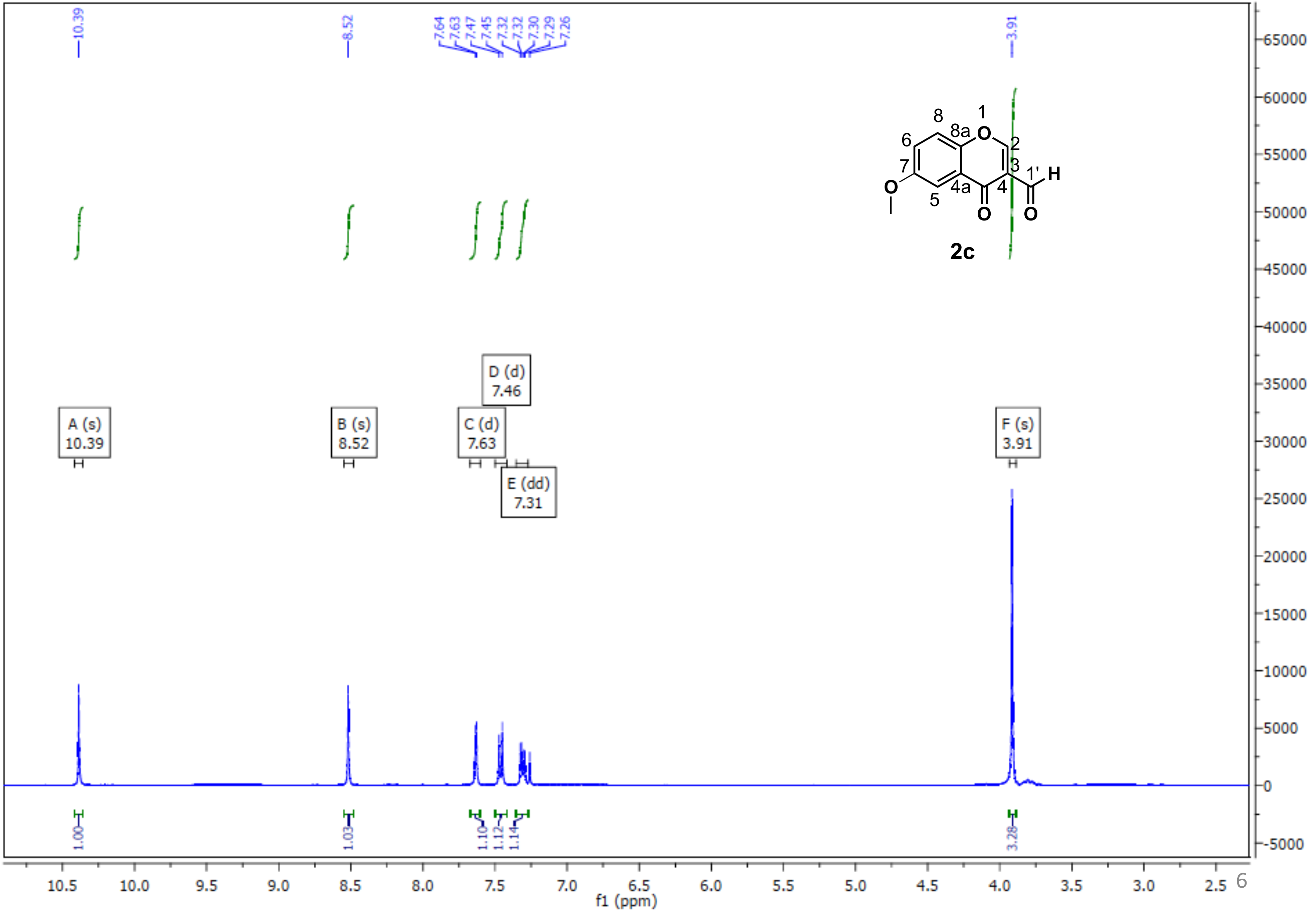

Figure S3.2. ^{13}C NMR spectra of compound 2c

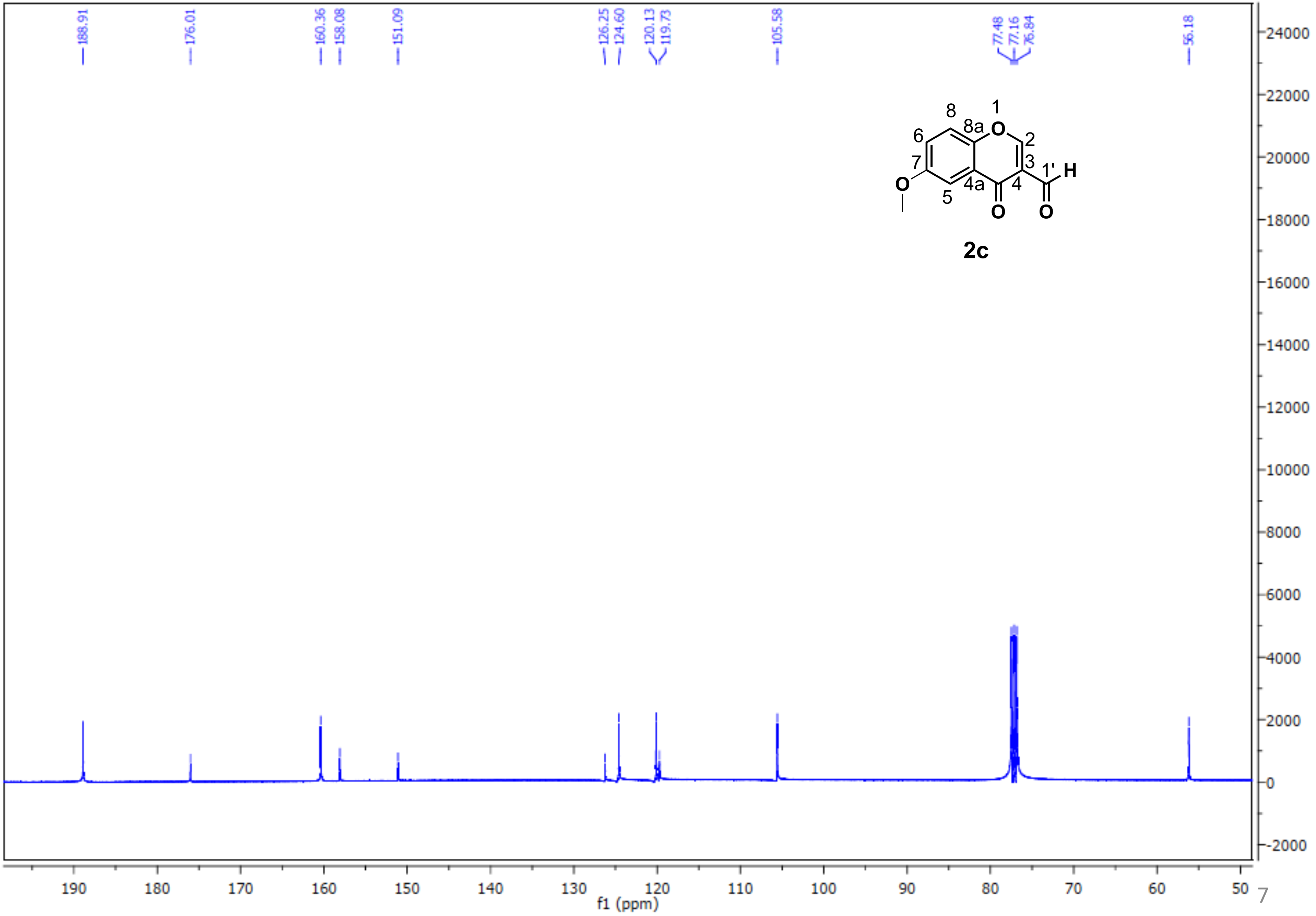

Figure S4.1. ^{1}H NMR spectra of compound 2d intermediate

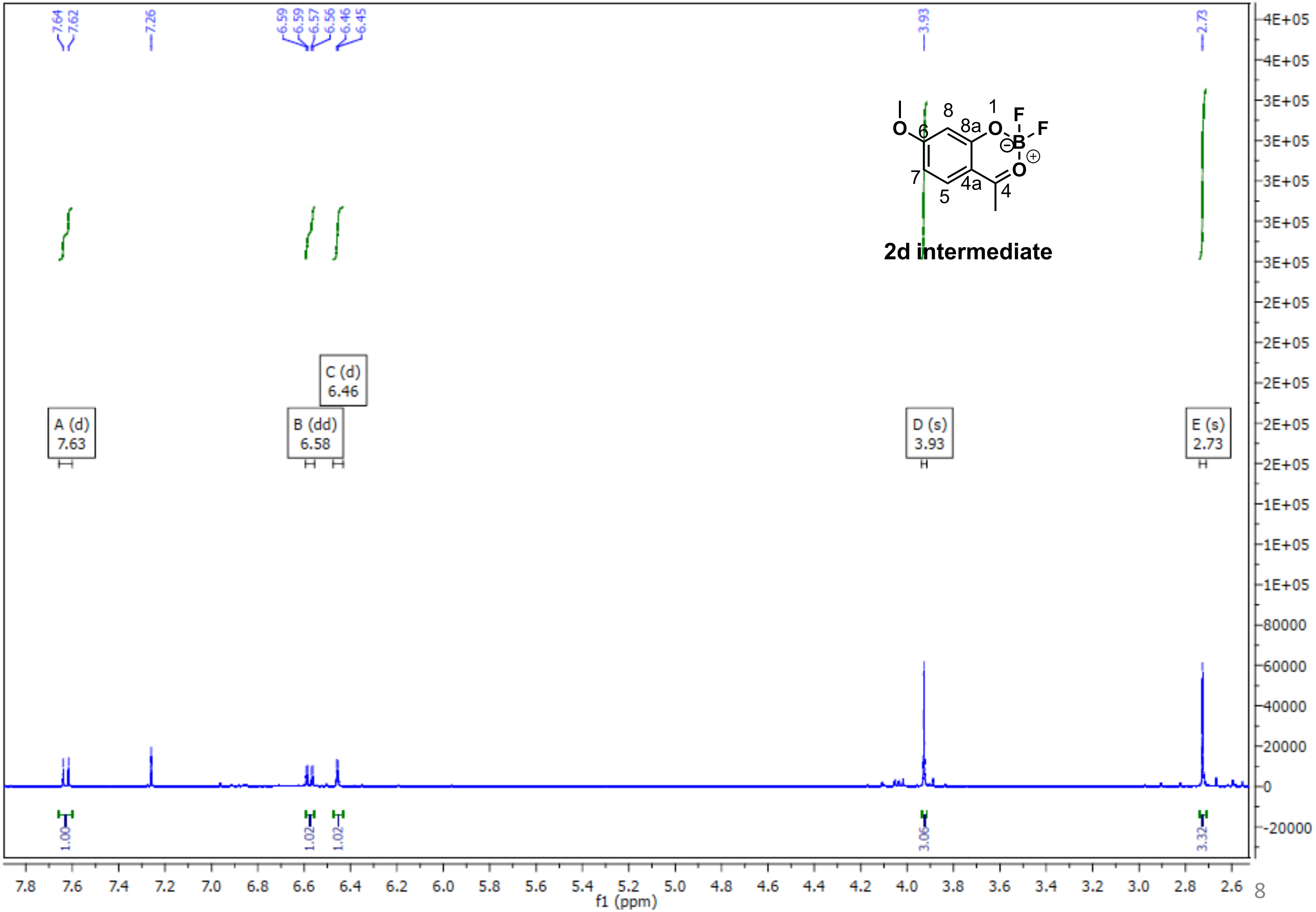

Figure S4.2. ^{1}H NMR spectra of compound 2d

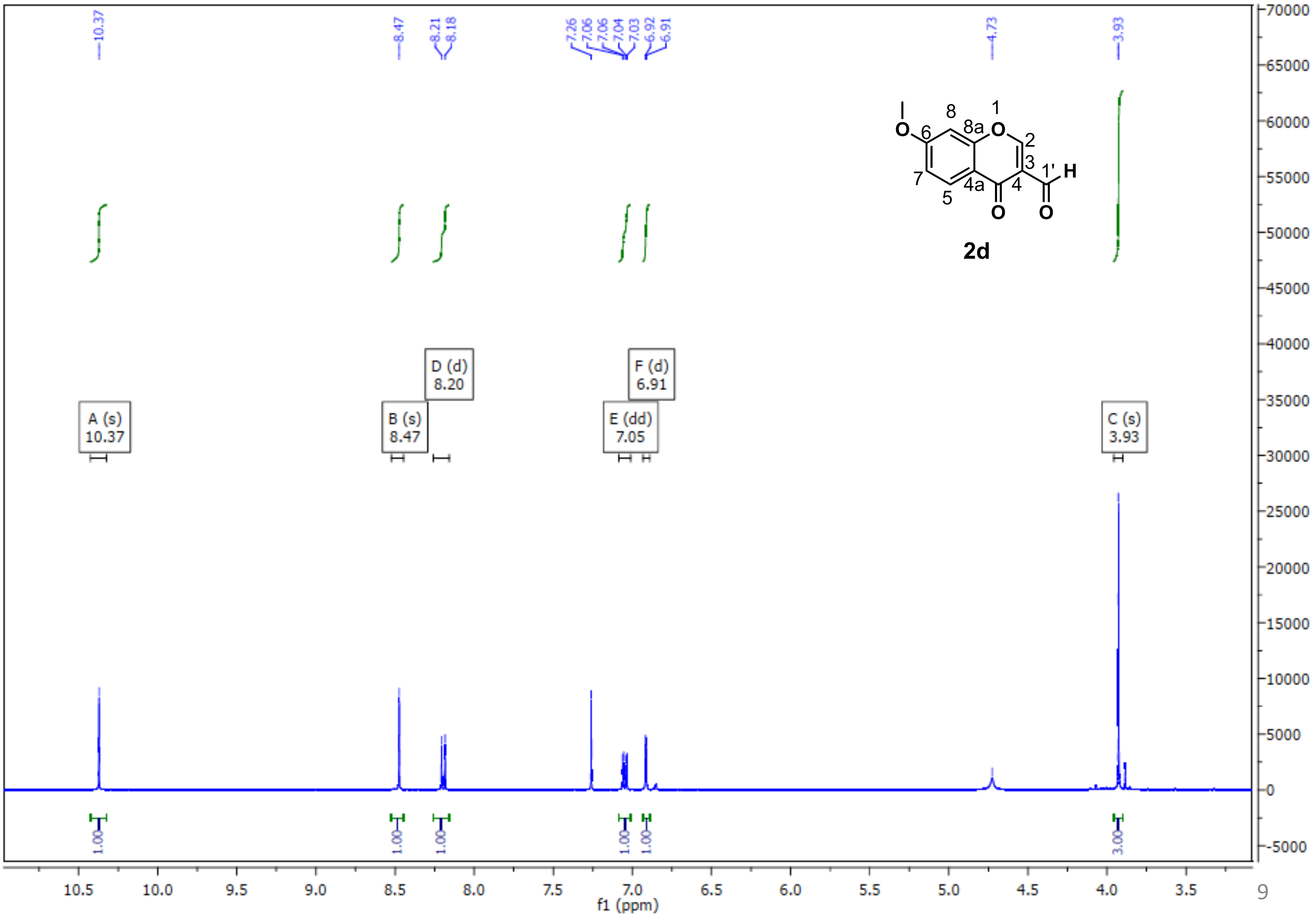

Figure S4.3. ^{13}C NMR spectra of compound 2d

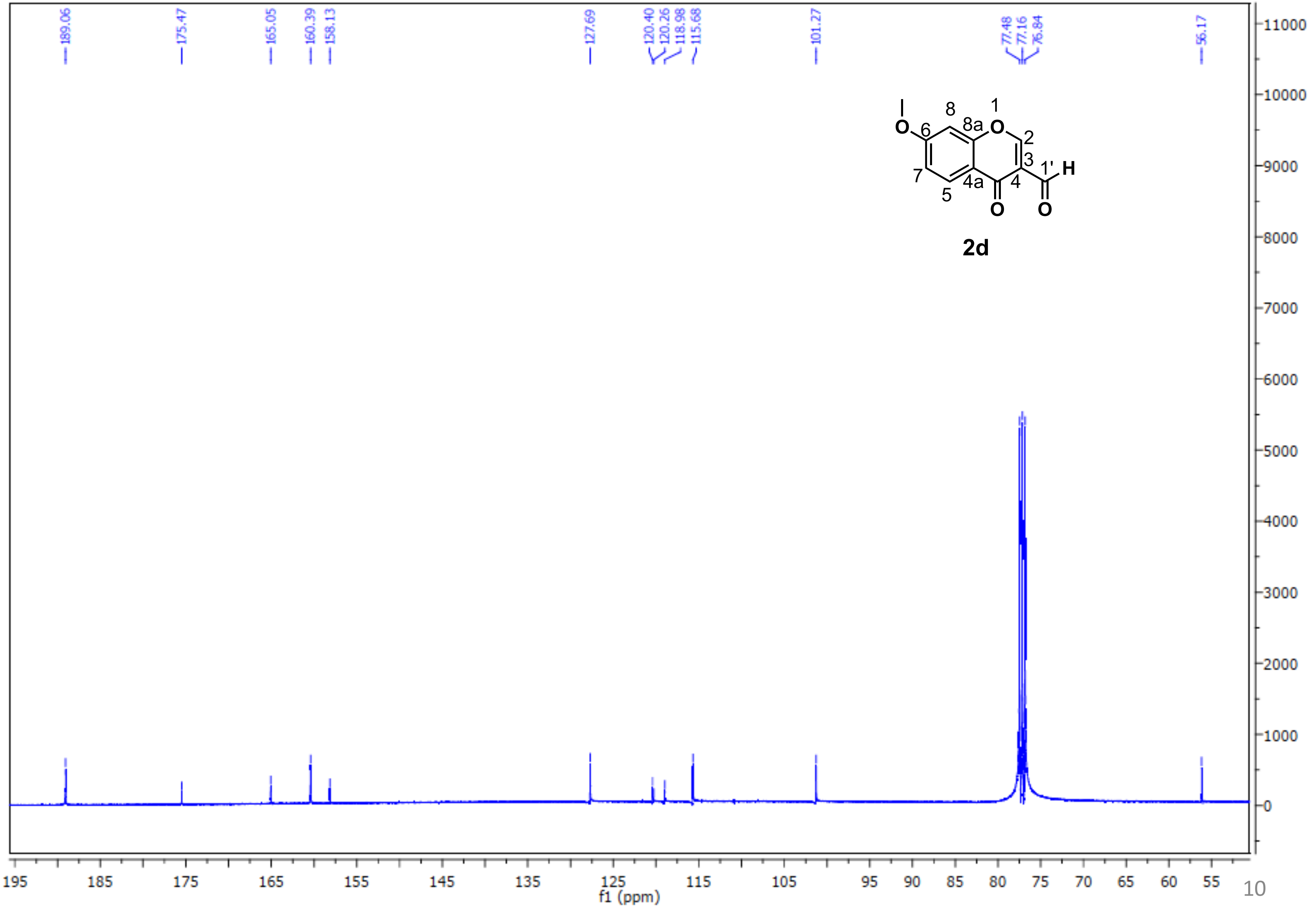

Figure S5.1. ^{1}H NMR spectra of compound 3a

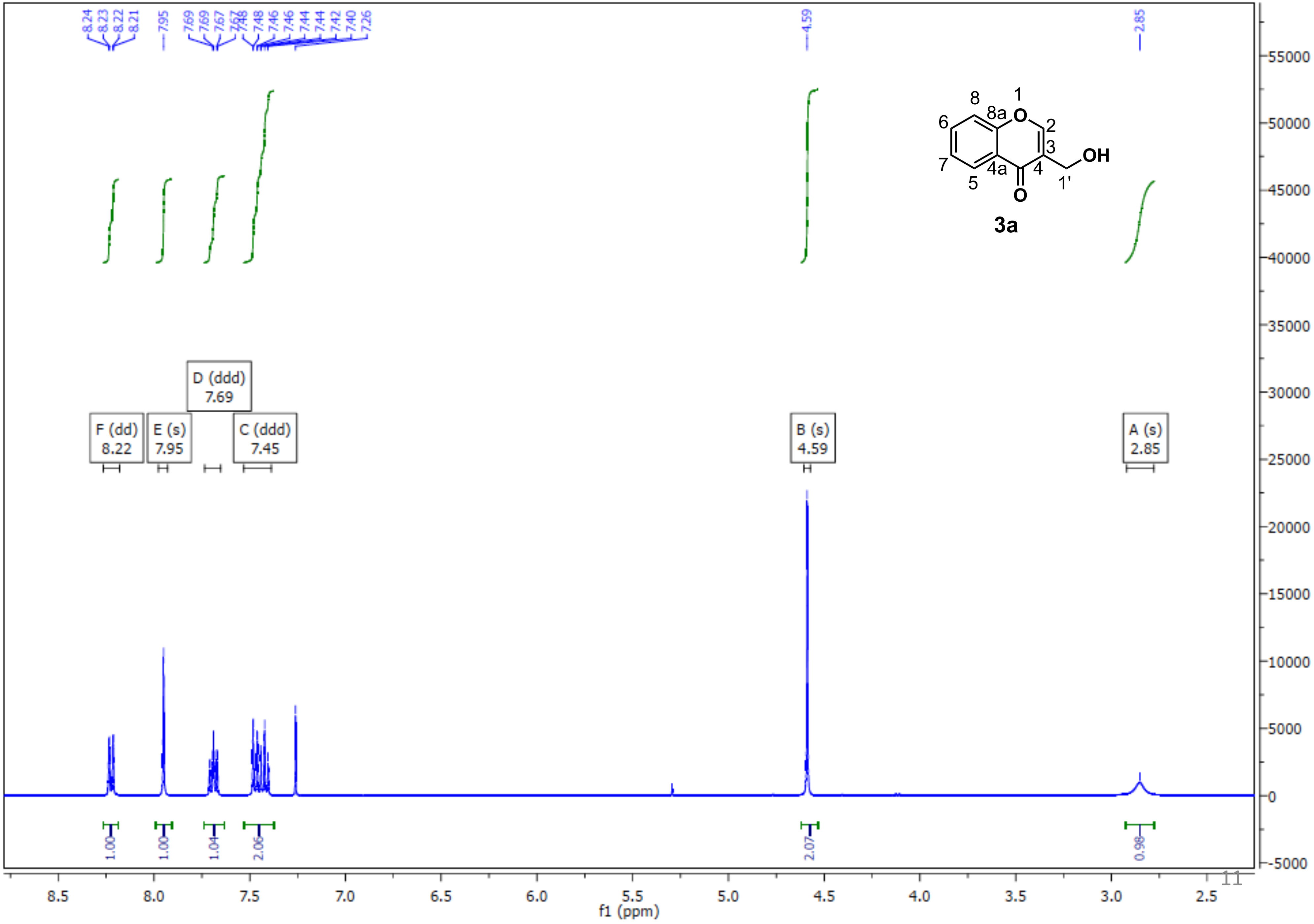

Figure S5.2. ^{13}C NMR spectra of compound 3a

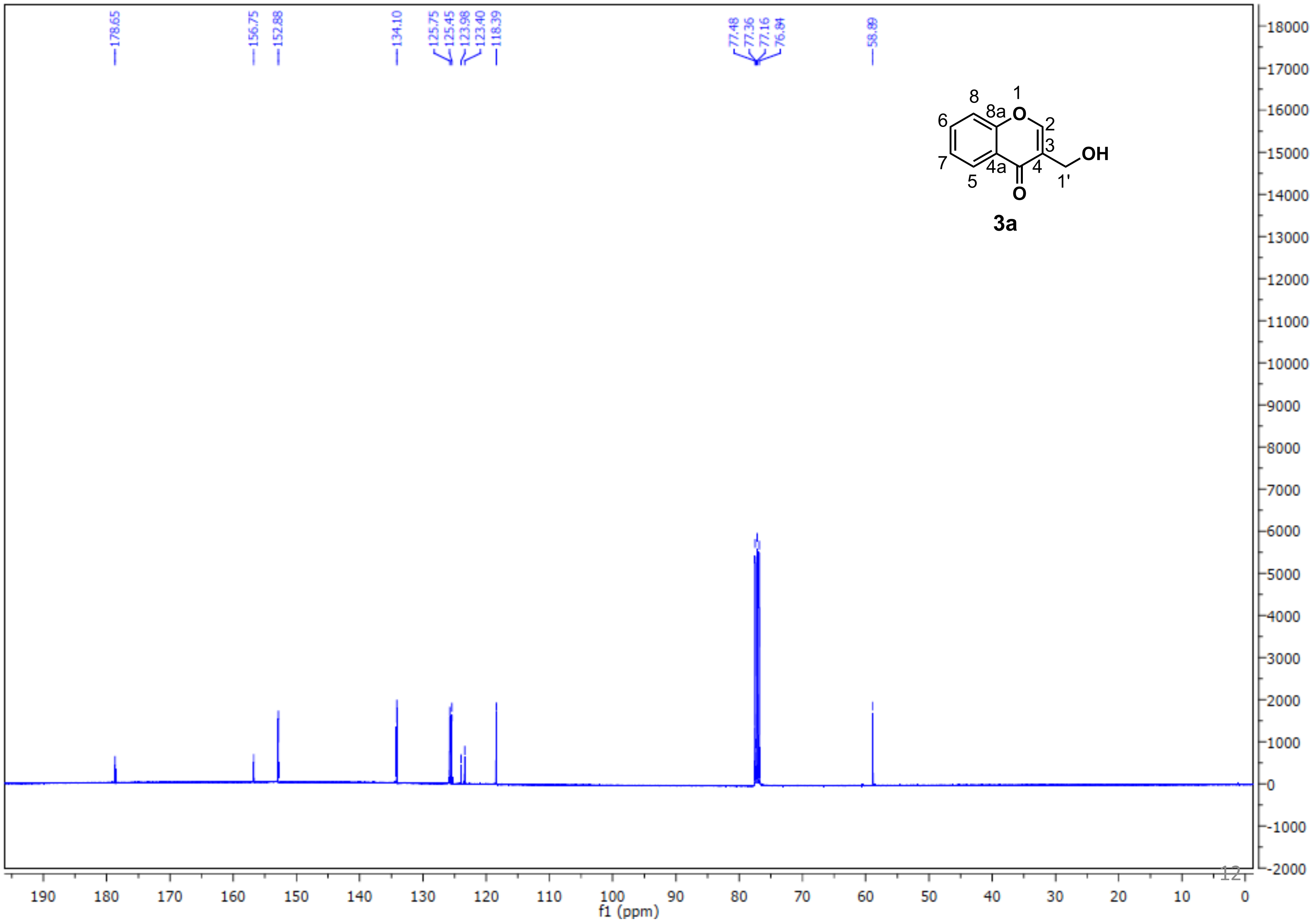

Figure S6.1. ^{1}H NMR spectra of compound 3b

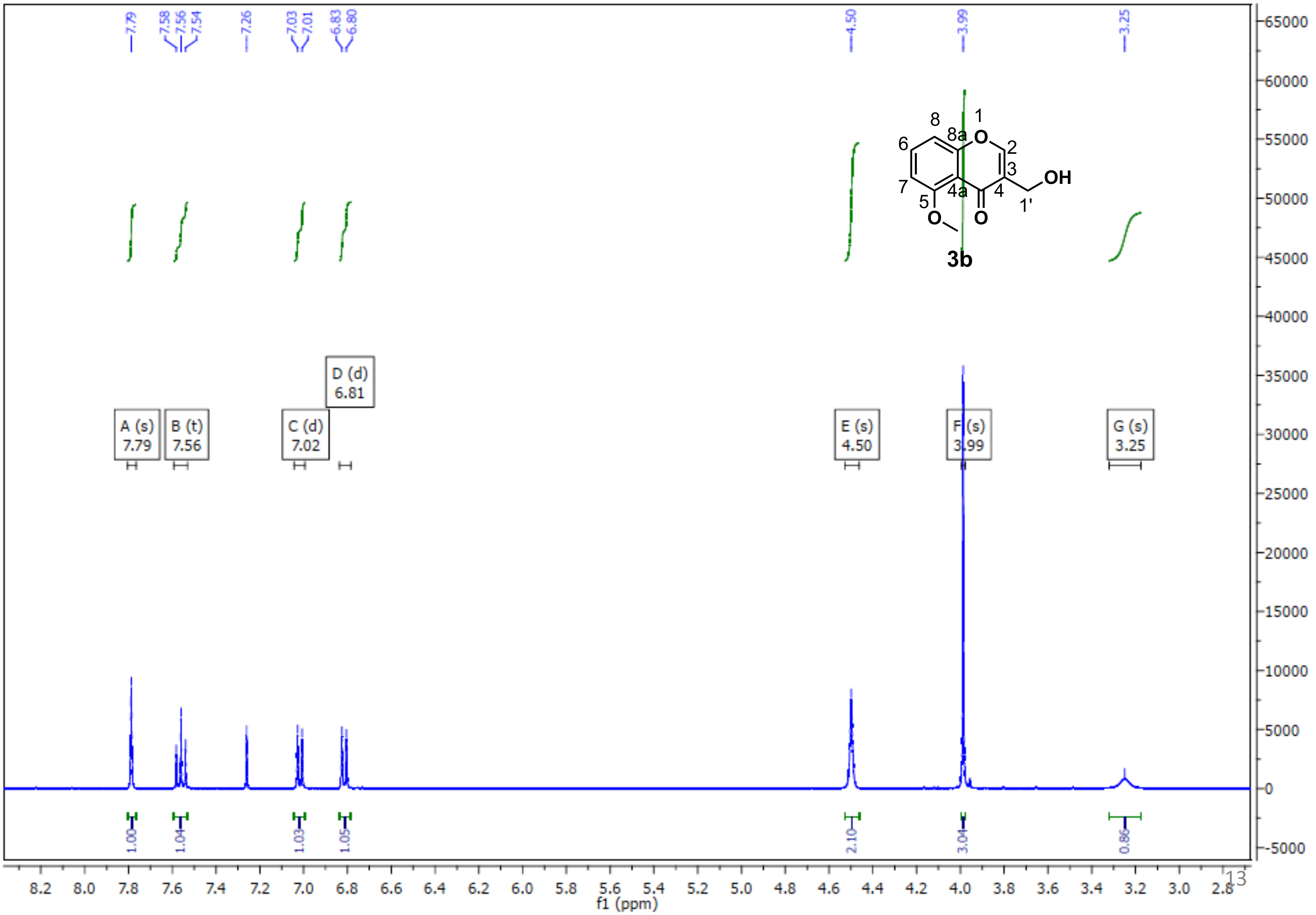

Figure S6.2. ^{13}C NMR spectra of compound 3b

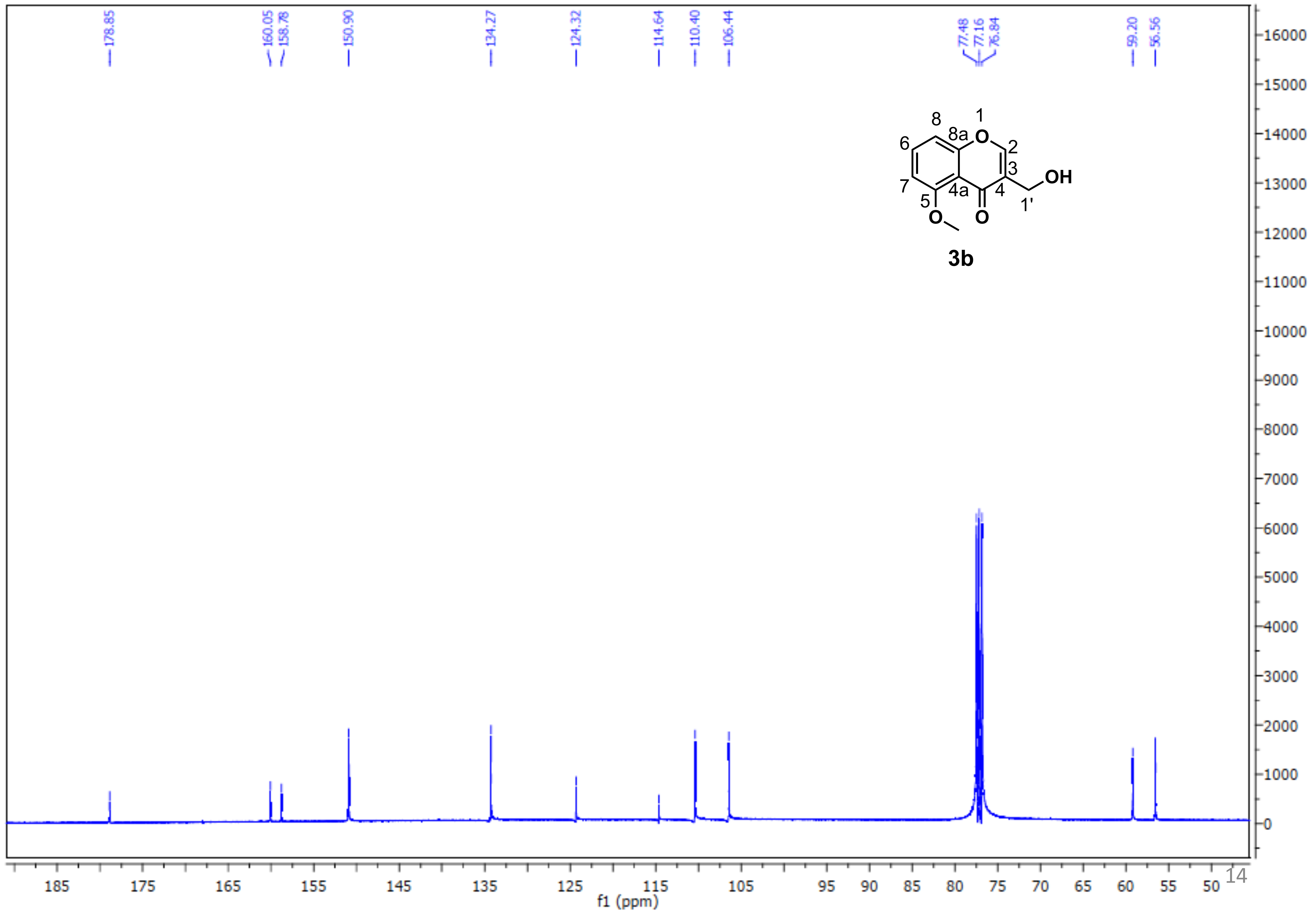

Figure S7.1. ^{1}H NMR spectra of compound 3c

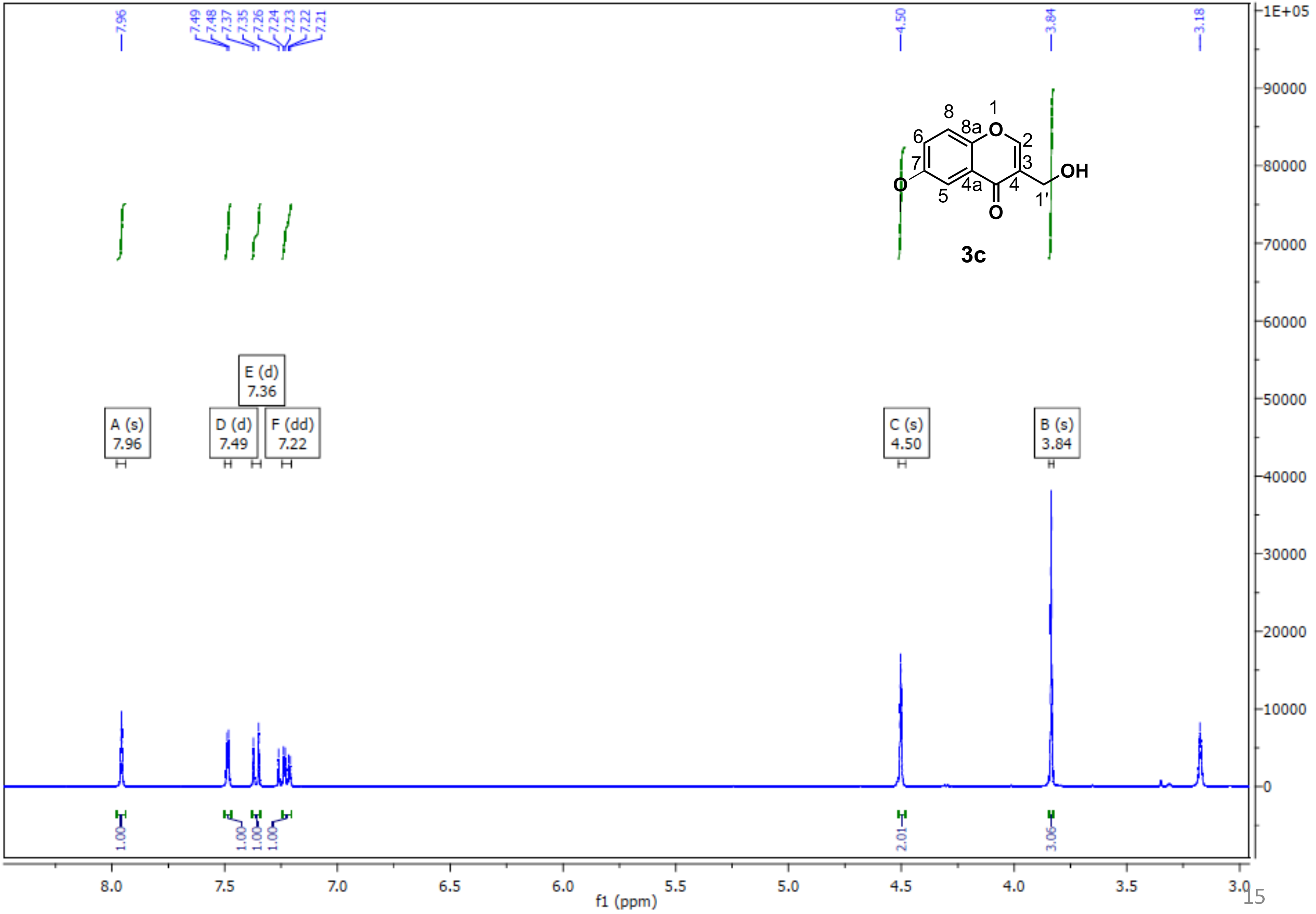

Figure S7.2. ^{1}H NMR spectra of compound 3c

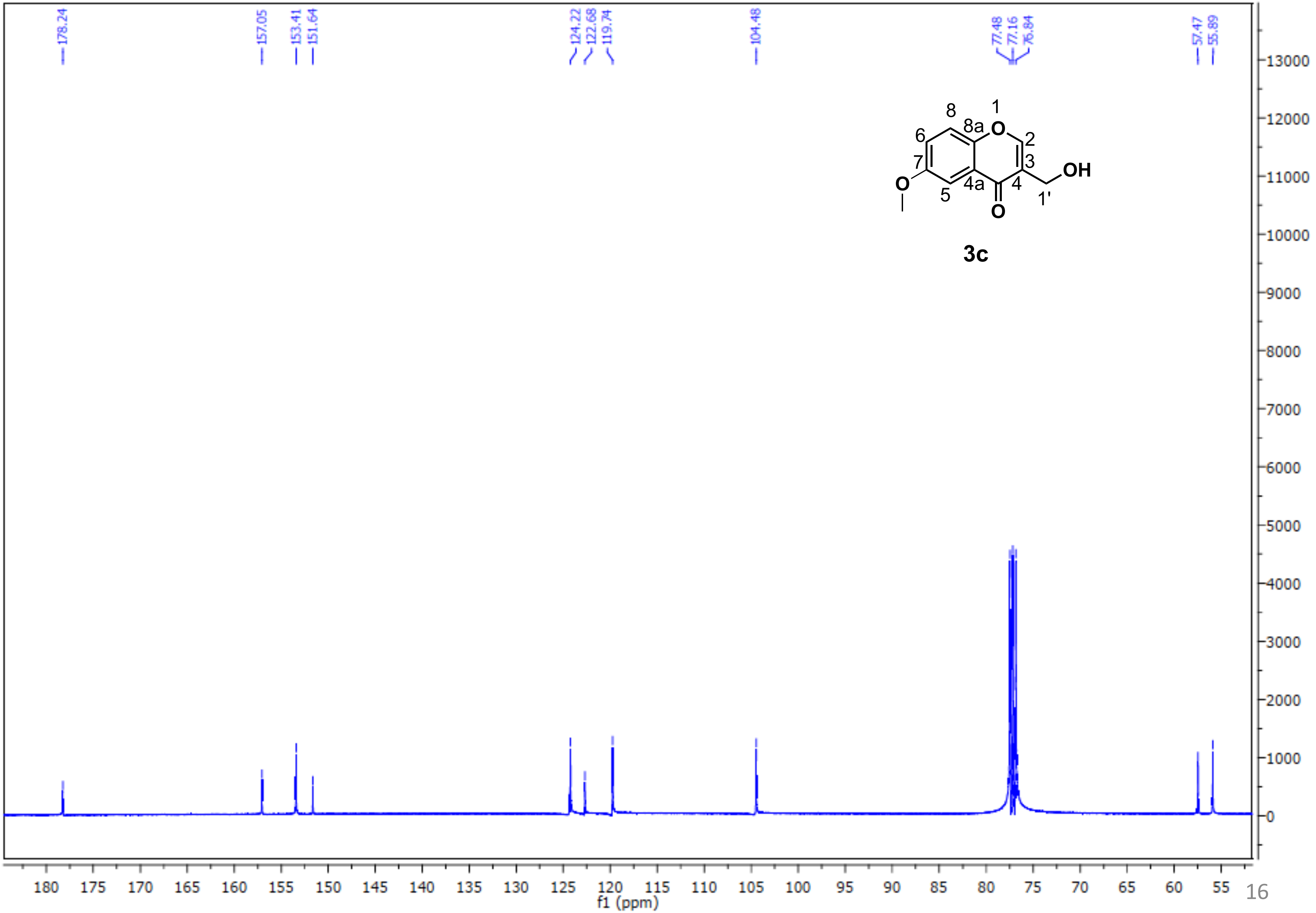

Figure S8.1. ^{1}H NMR spectra of compound 3d

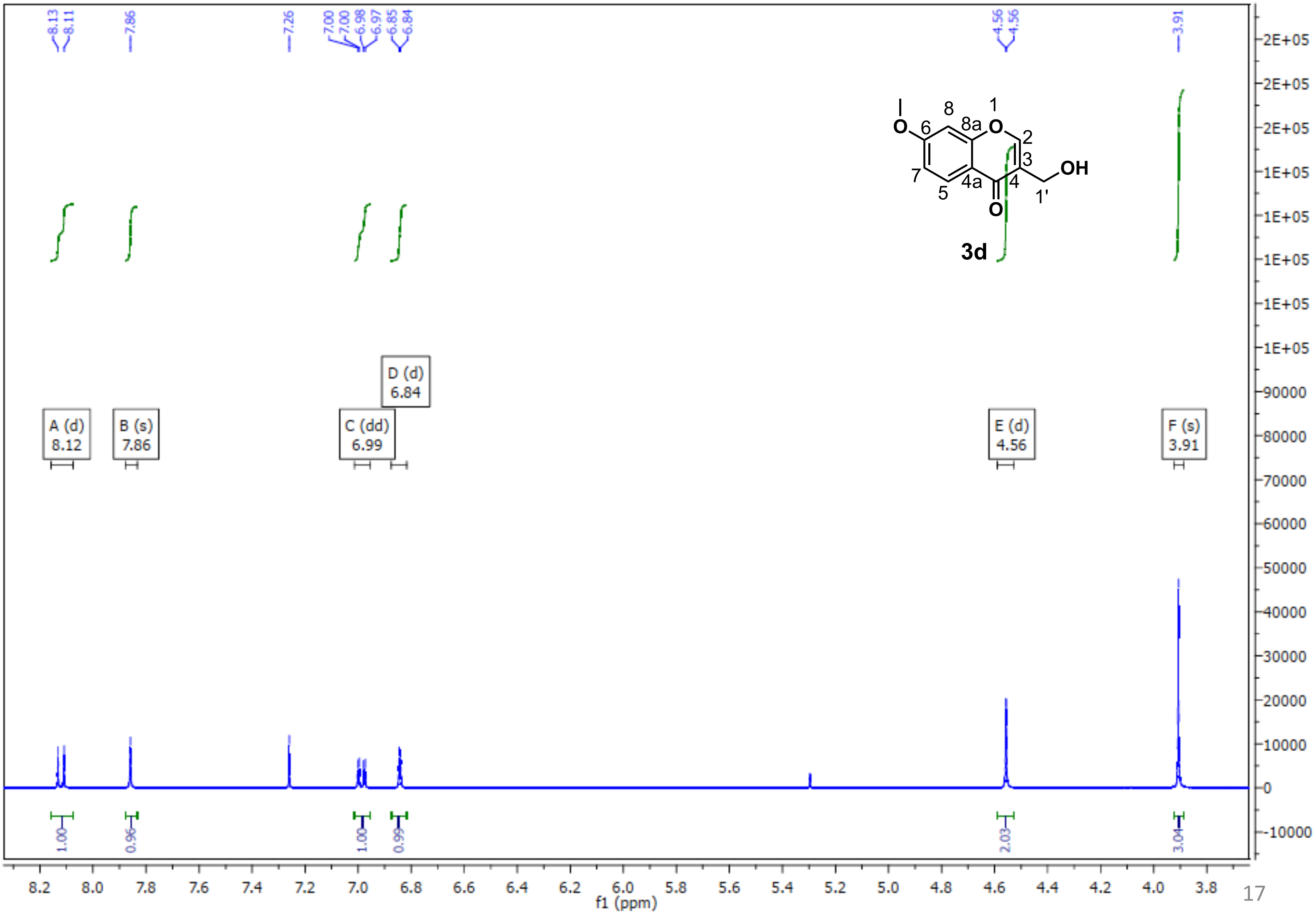

Figure S8.2. ^{13}C NMR spectra of compound 3d

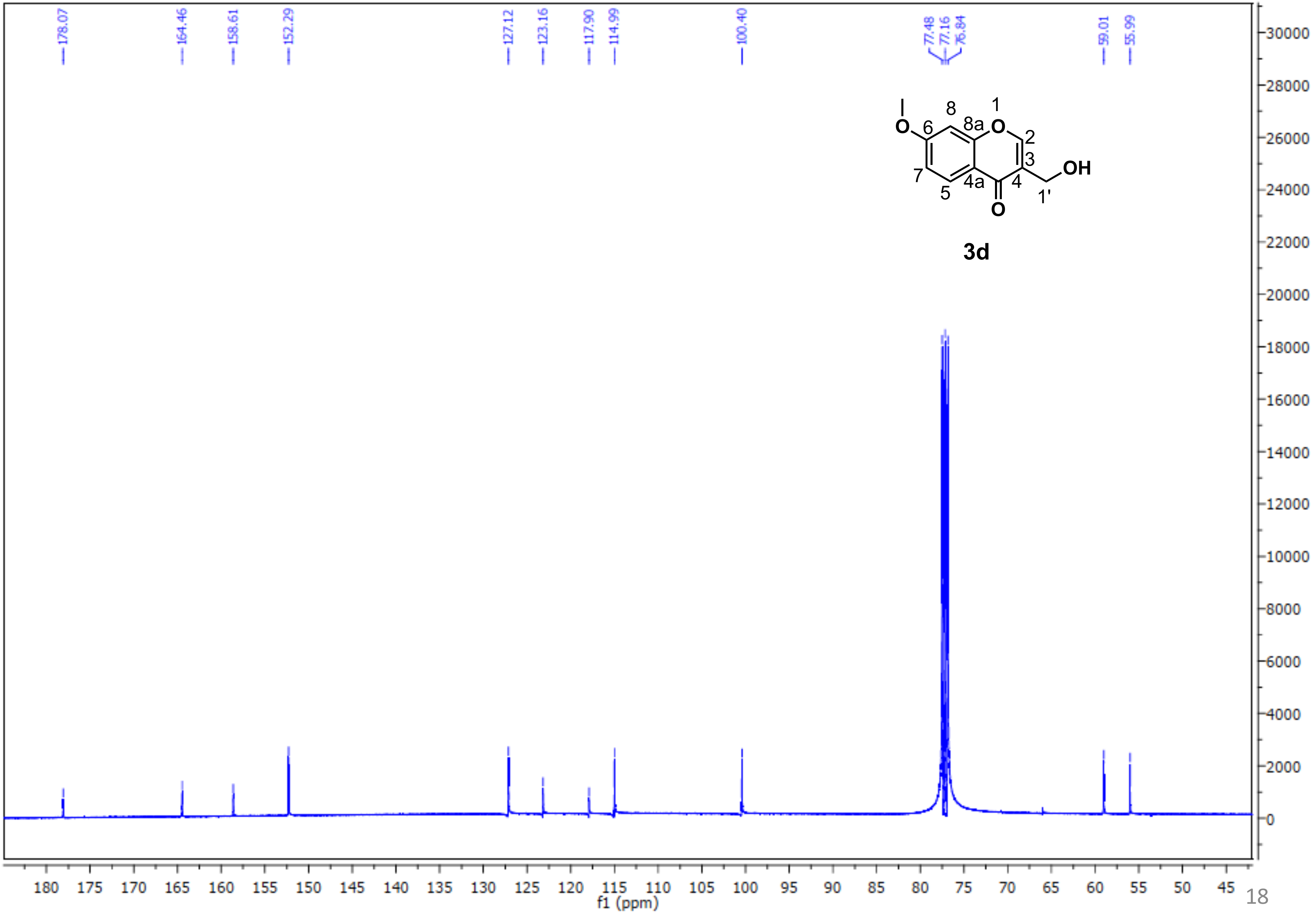

Figure S9.1. ^{1}H NMR spectra of compound 4a

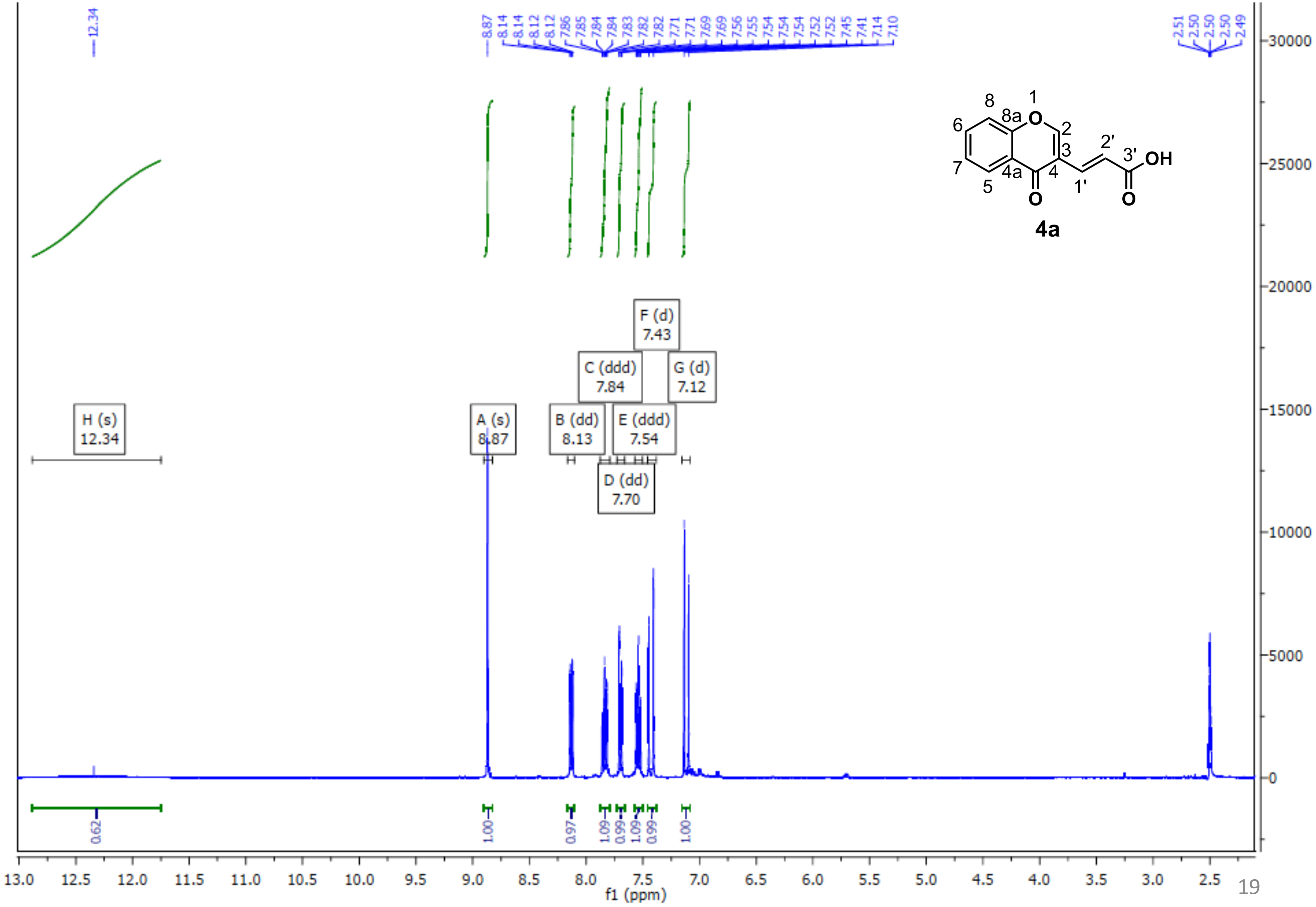

Figure S9.2. ^{13}C NMR spectra of compound 4a

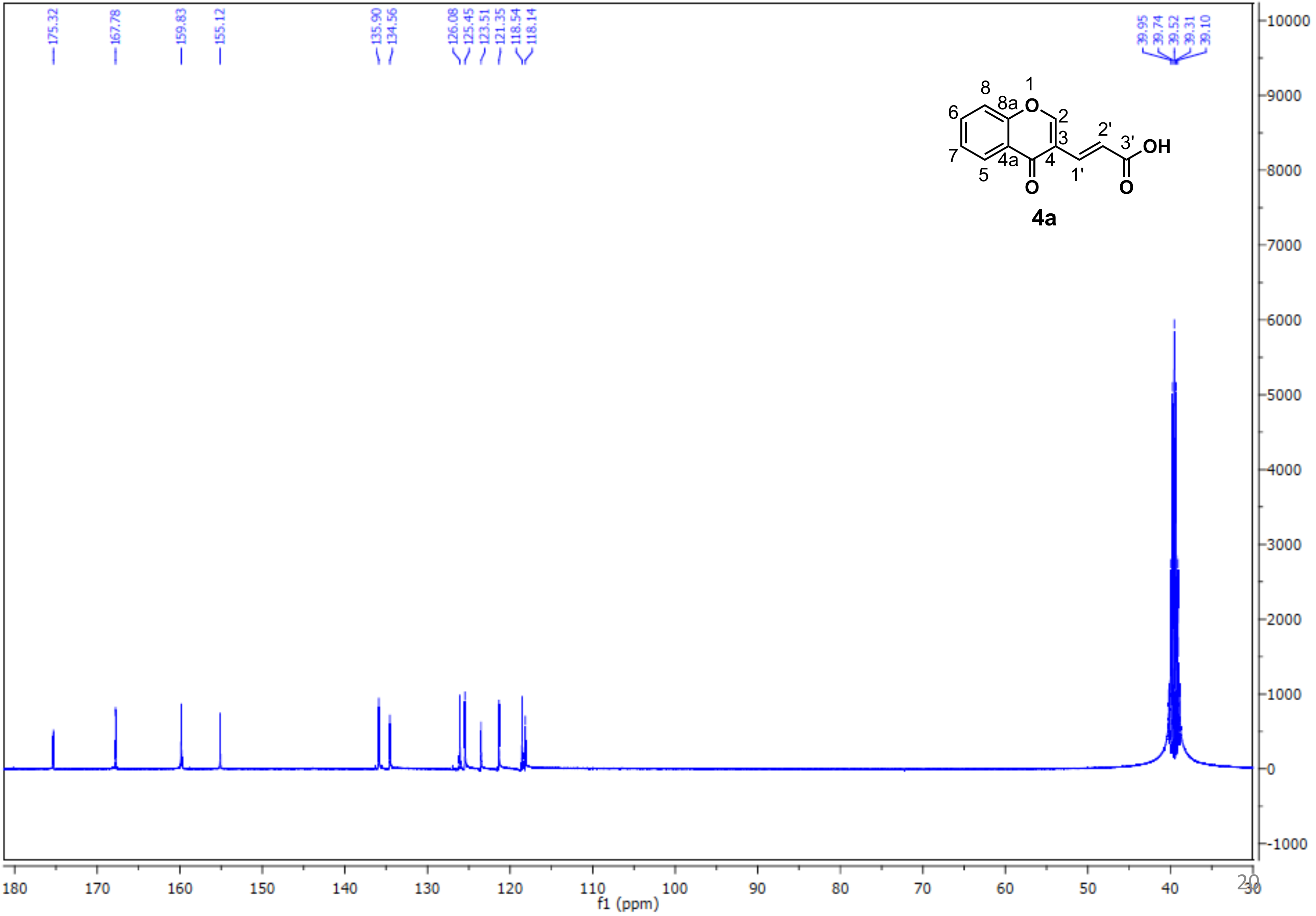

Figure S10.1. ^{1}H NMR spectra of compound 4b

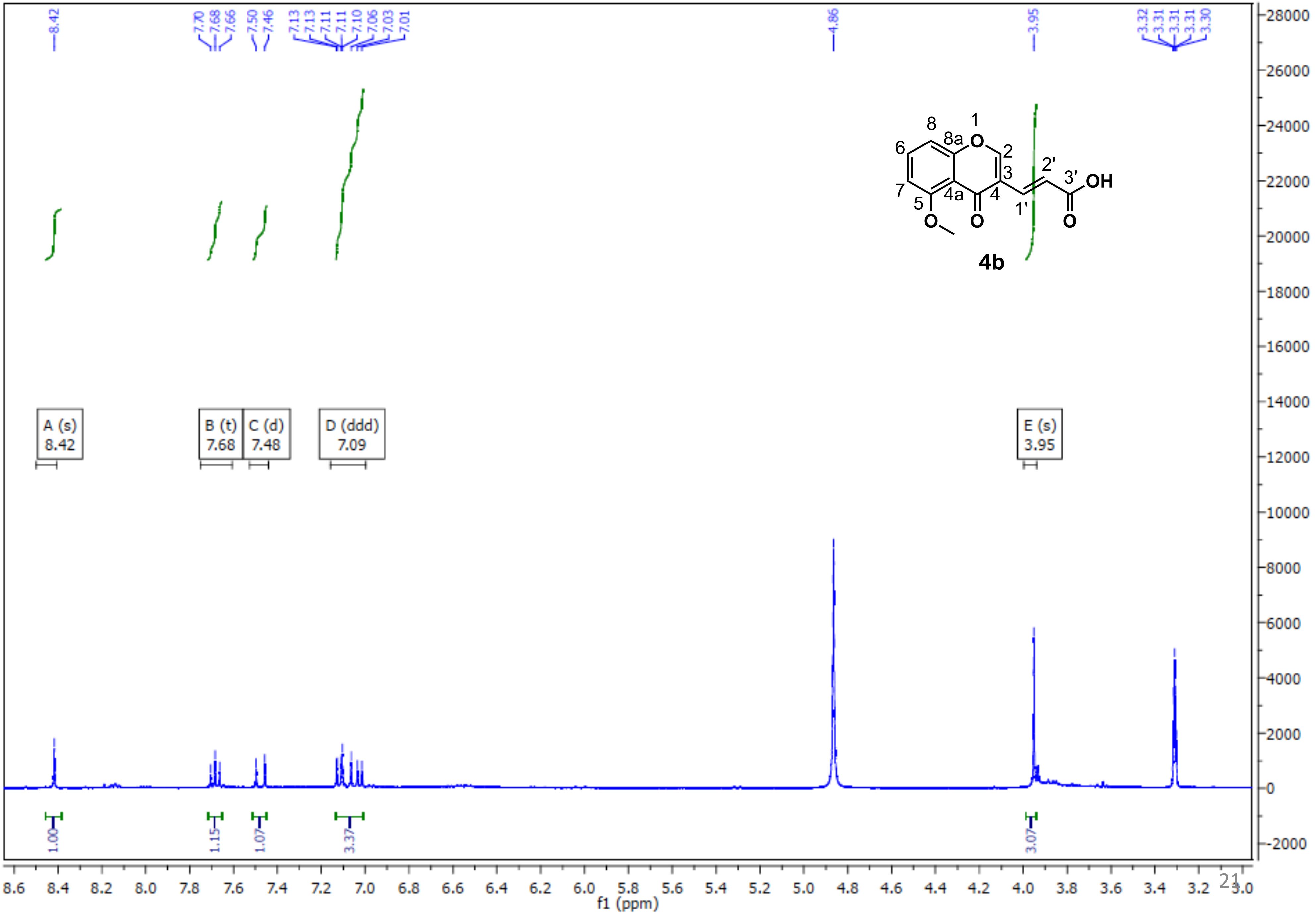

Figure S10.2. ^{13}C NMR spectra of compound 4b

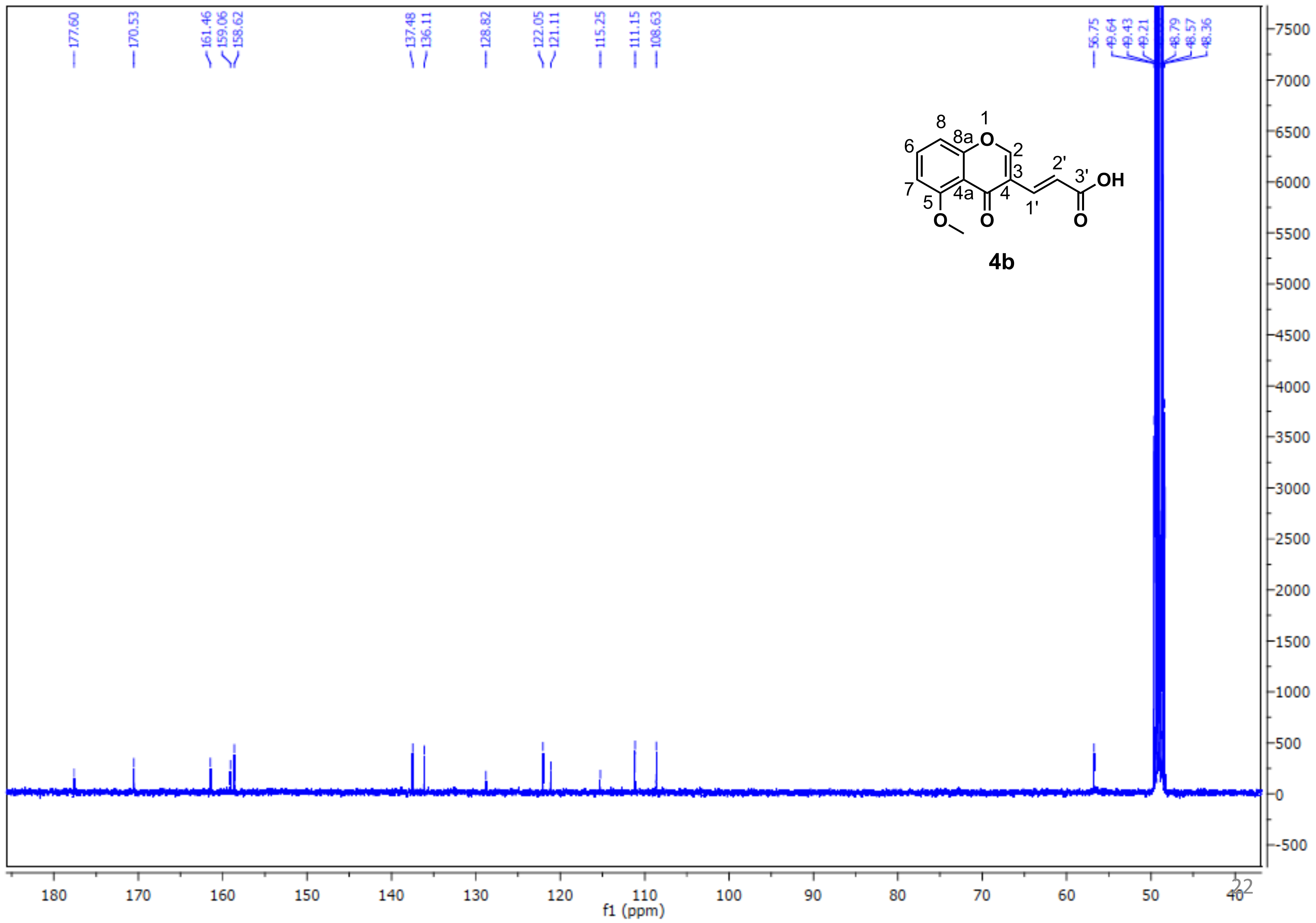

Figure S11.1. ^{1}H NMR spectra of compound 4c

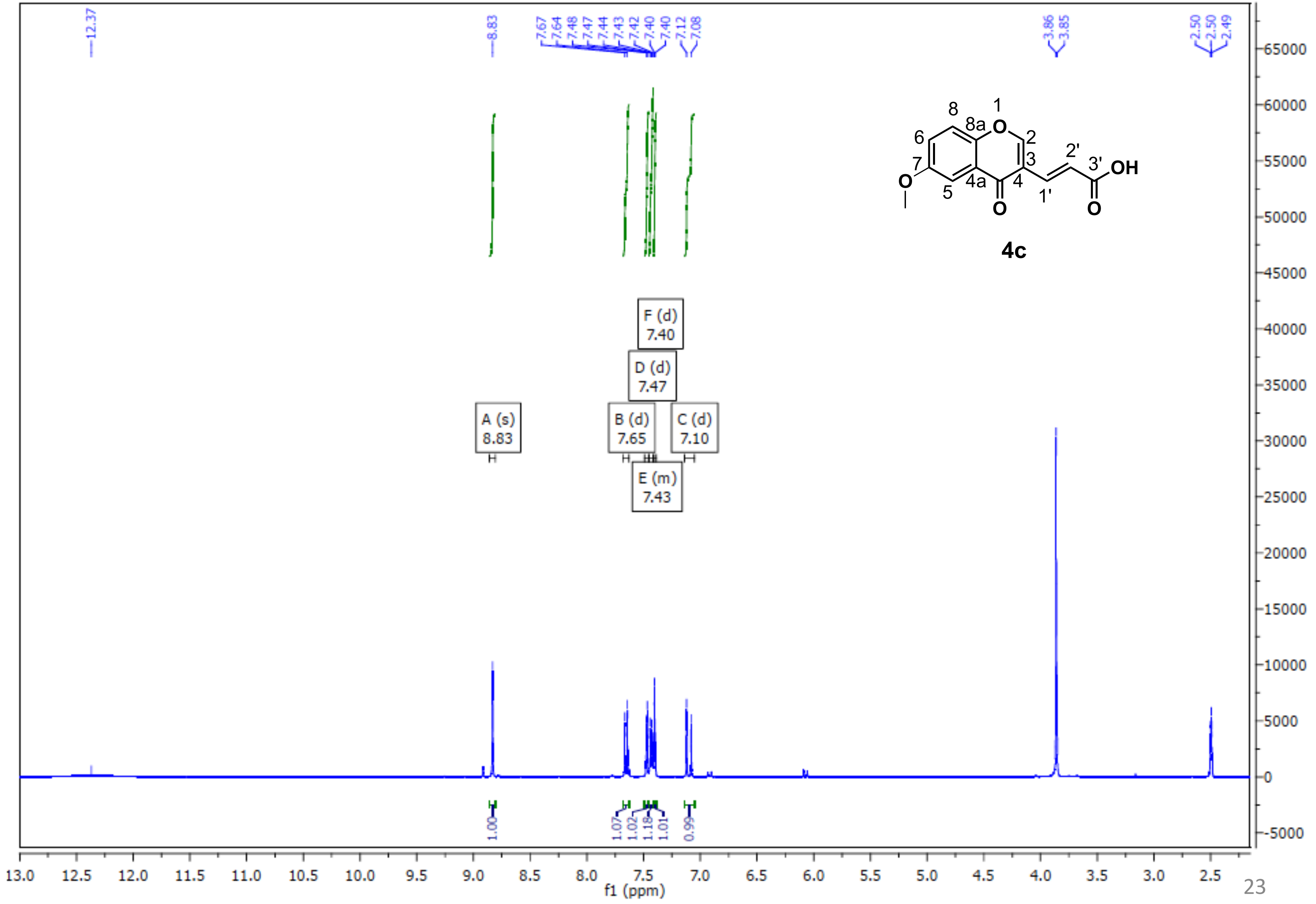

Figure S11.2. ^{13}C NMR spectra of compound 4c

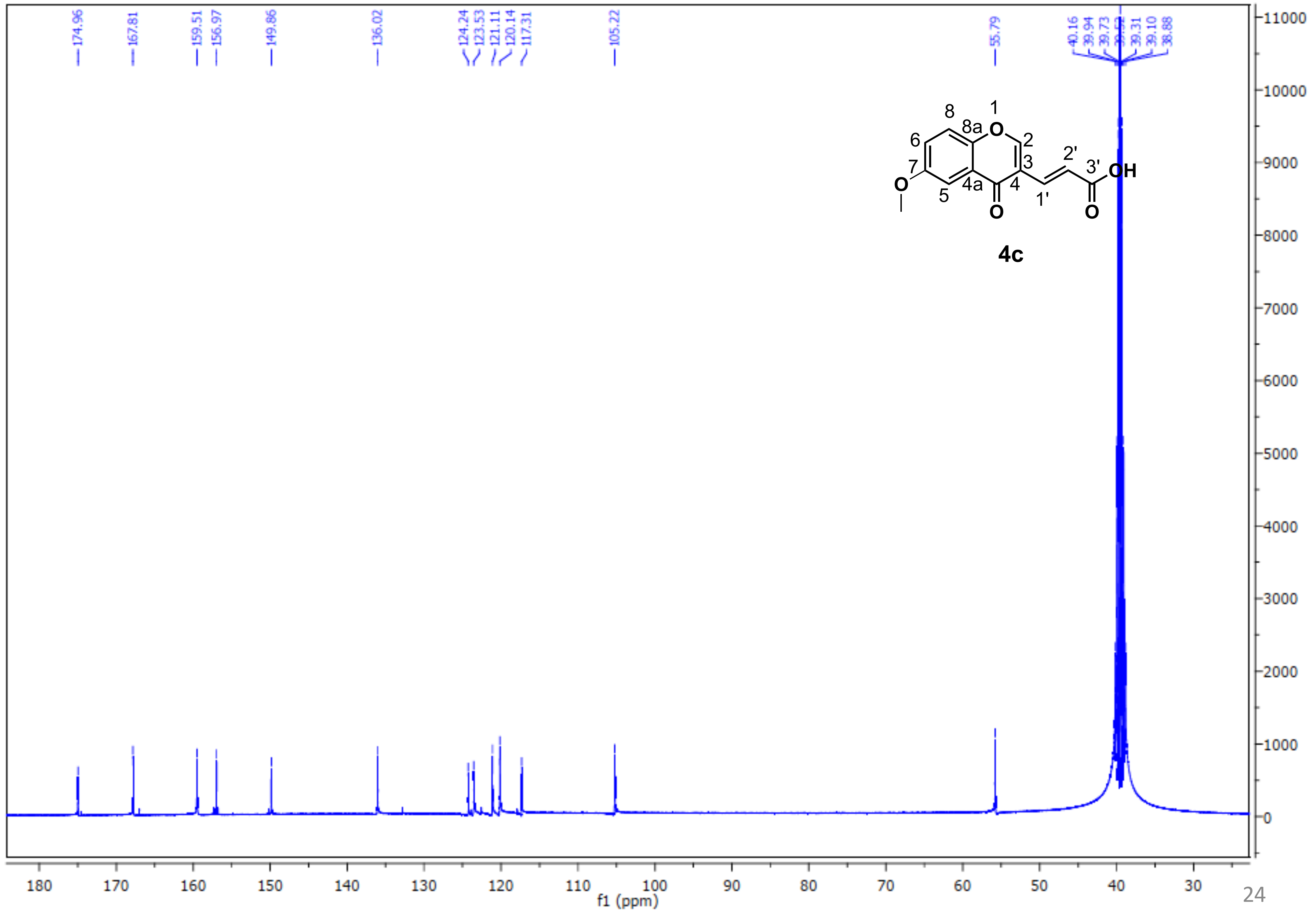

Figure S12.1. ^{1}H NMR spectra of compound 4d

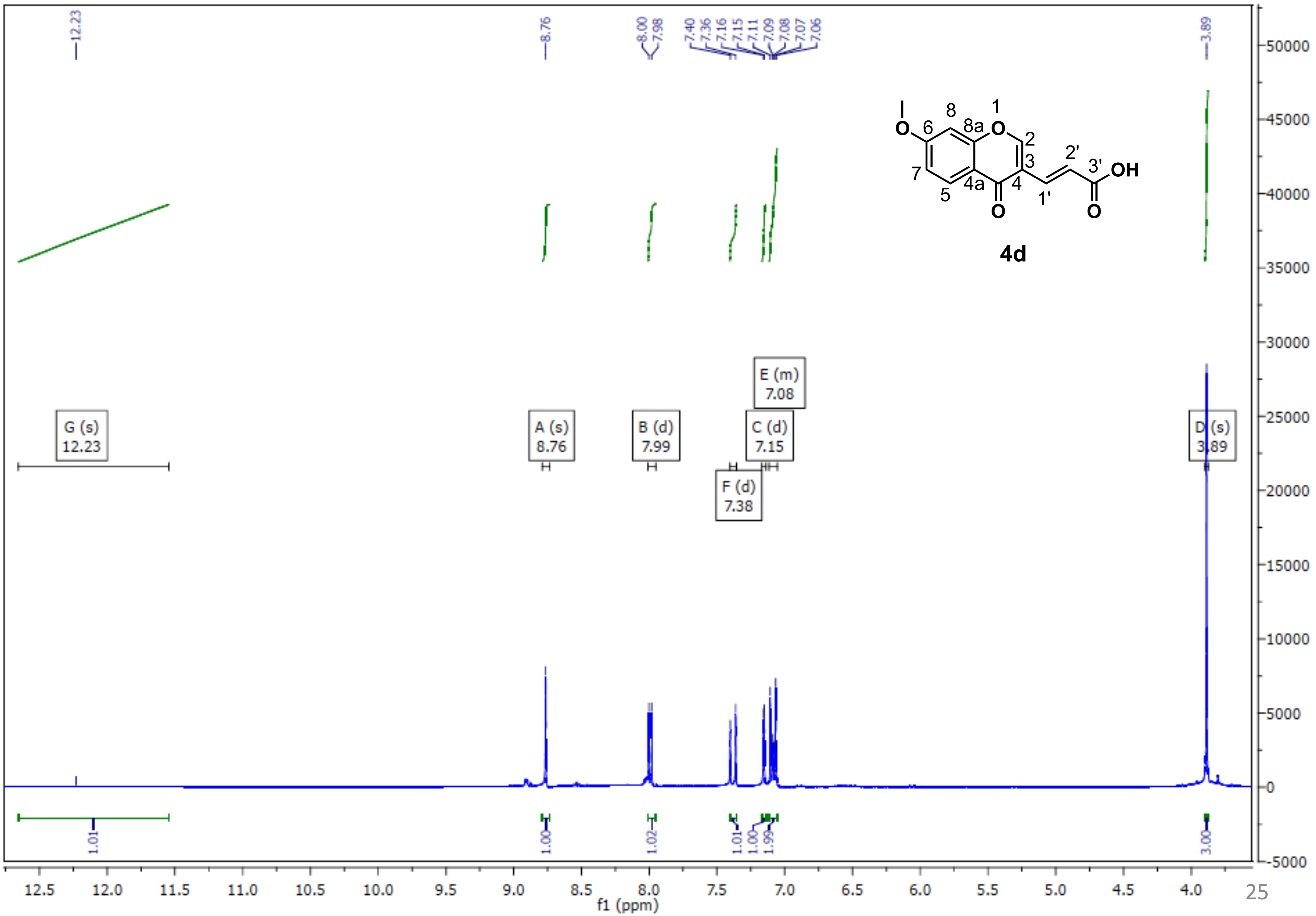

Figure S12.2. ^{13}C NMR spectra of compound 4d

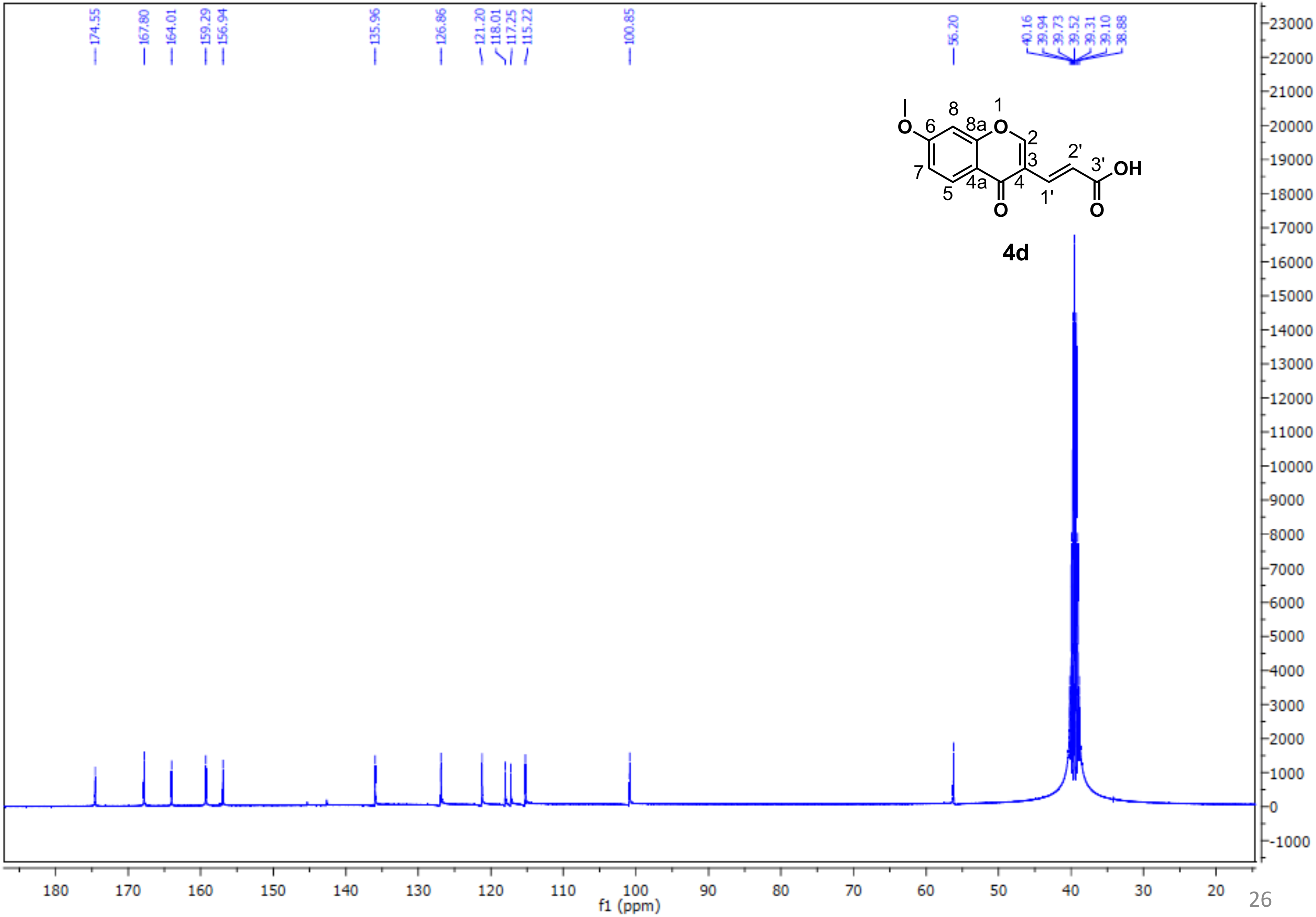

Figure S12.3. ^{13}C-DEPT NMR spectra of compound 4d

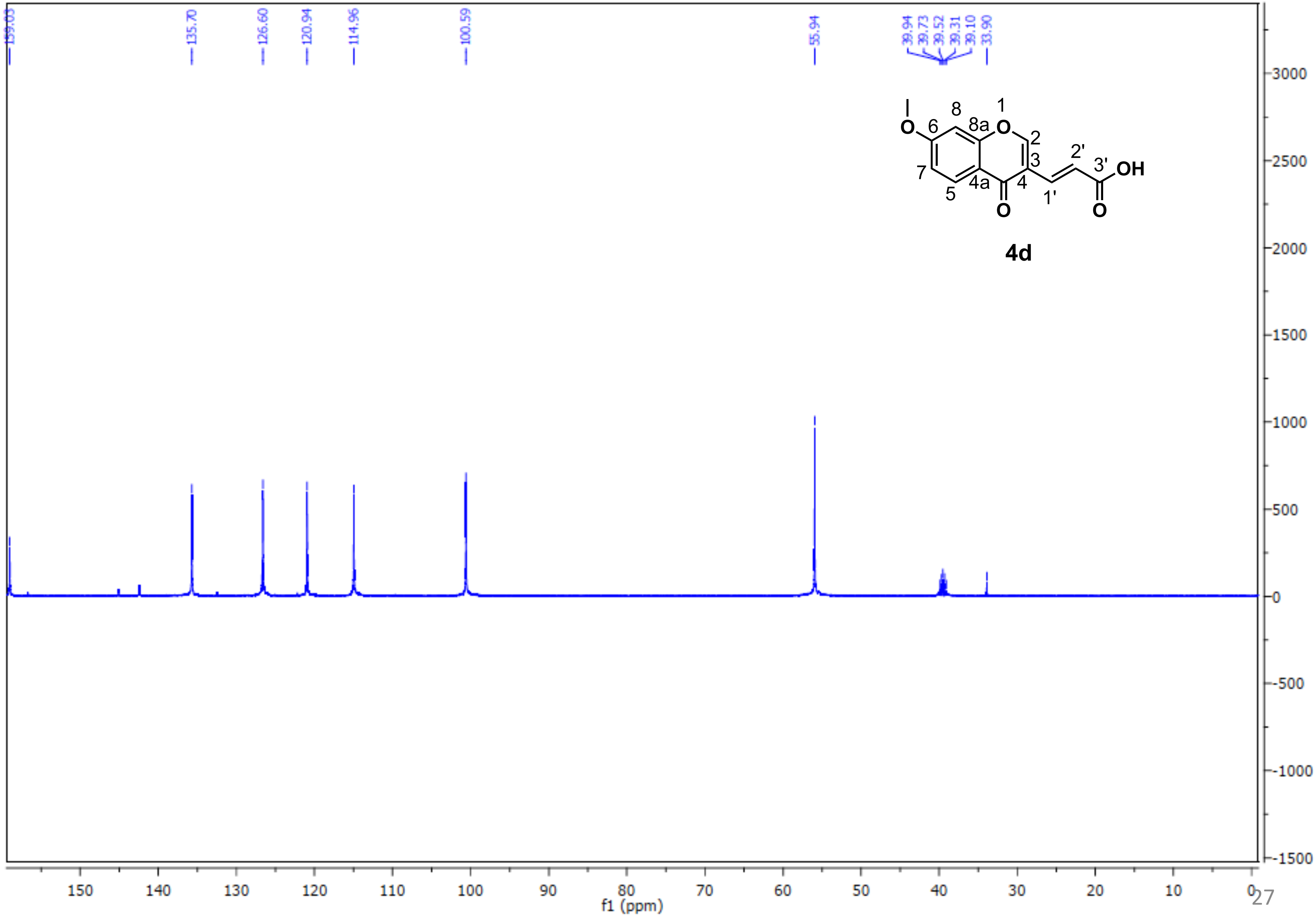

Figure S13.1. ^{1}H NMR spectra of compound 5a

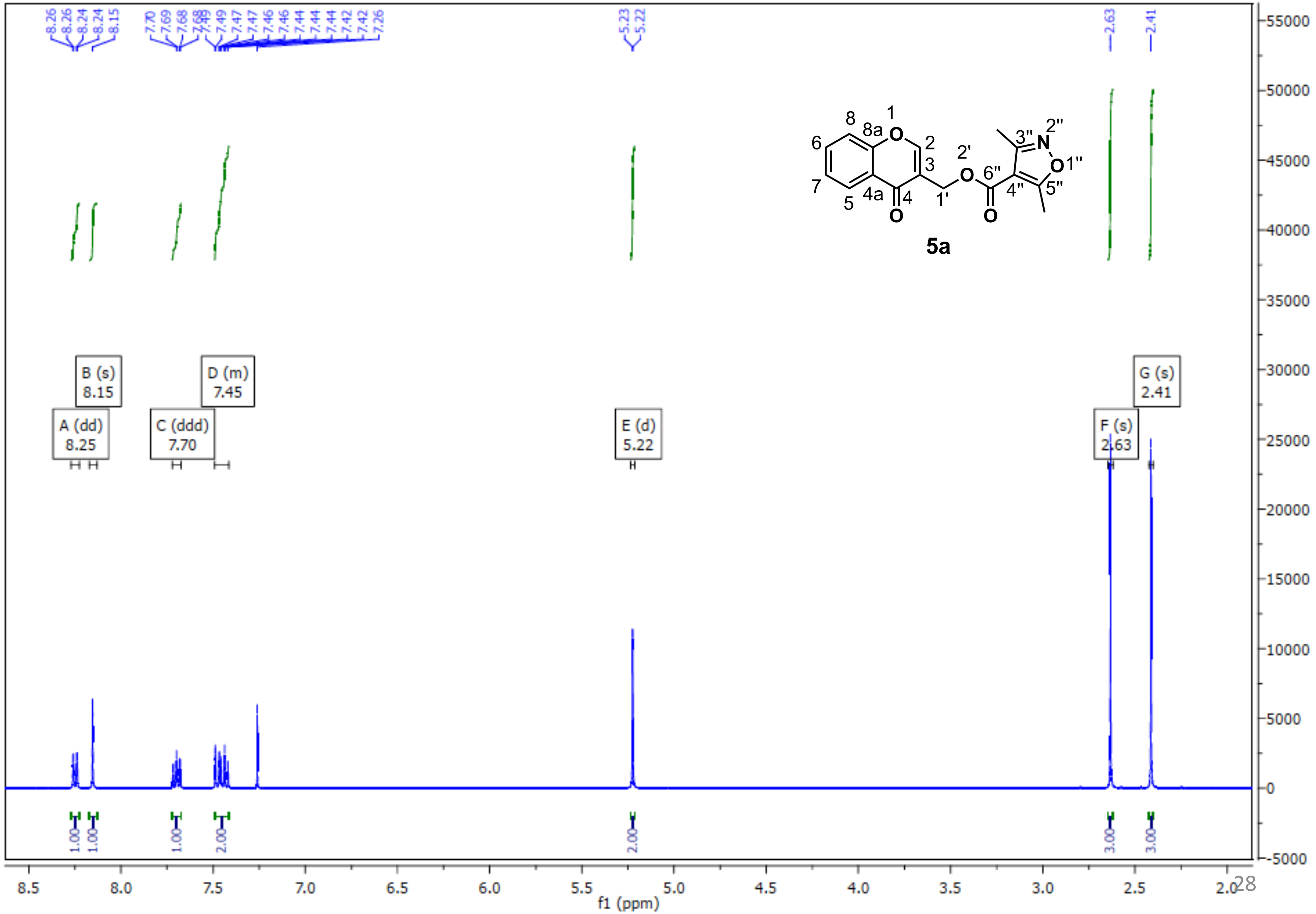

Figure S13.2. ^{13}C NMR spectra of compound 5a

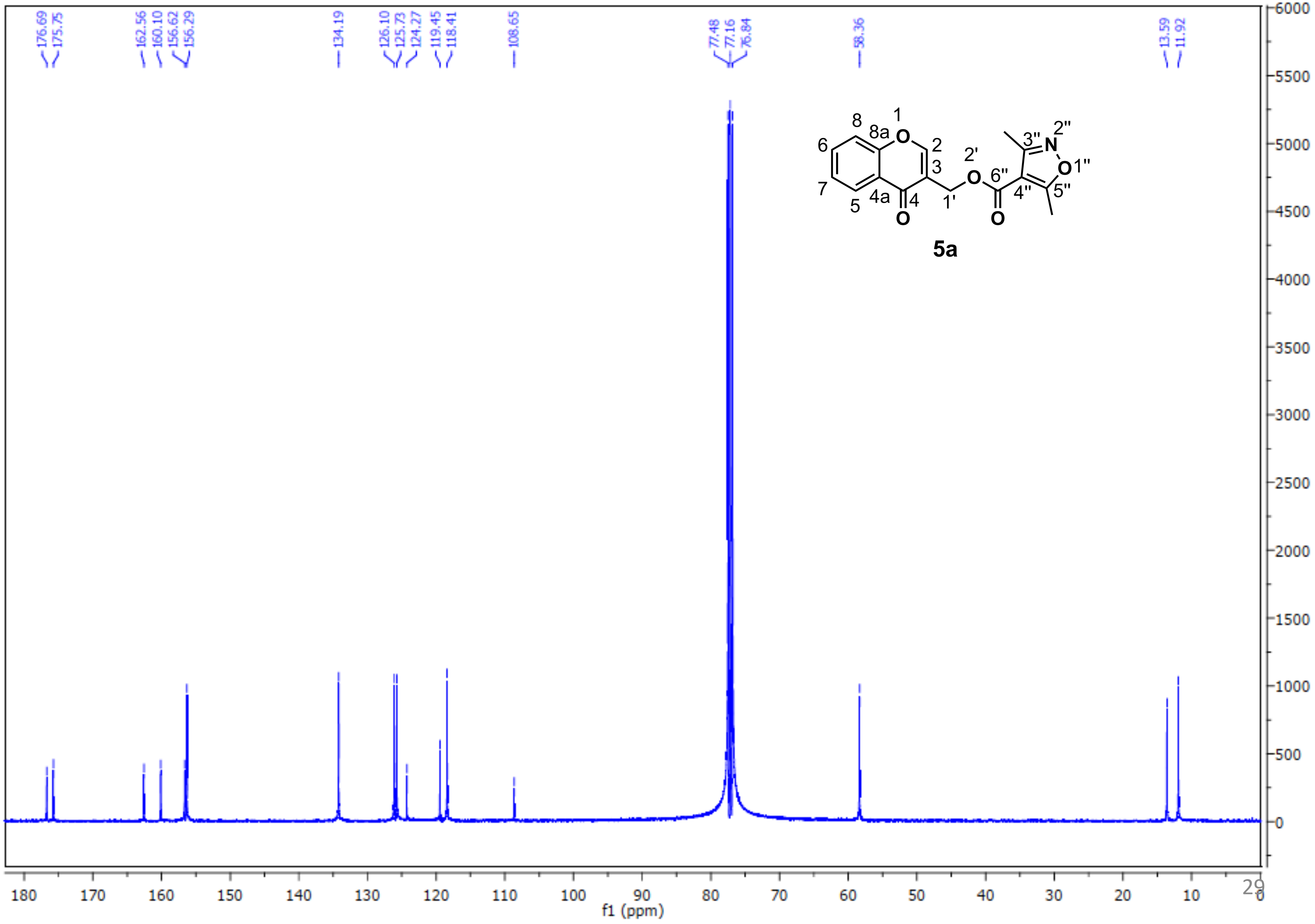

Figure S14.1. ^{1}H NMR spectra of compound 5b

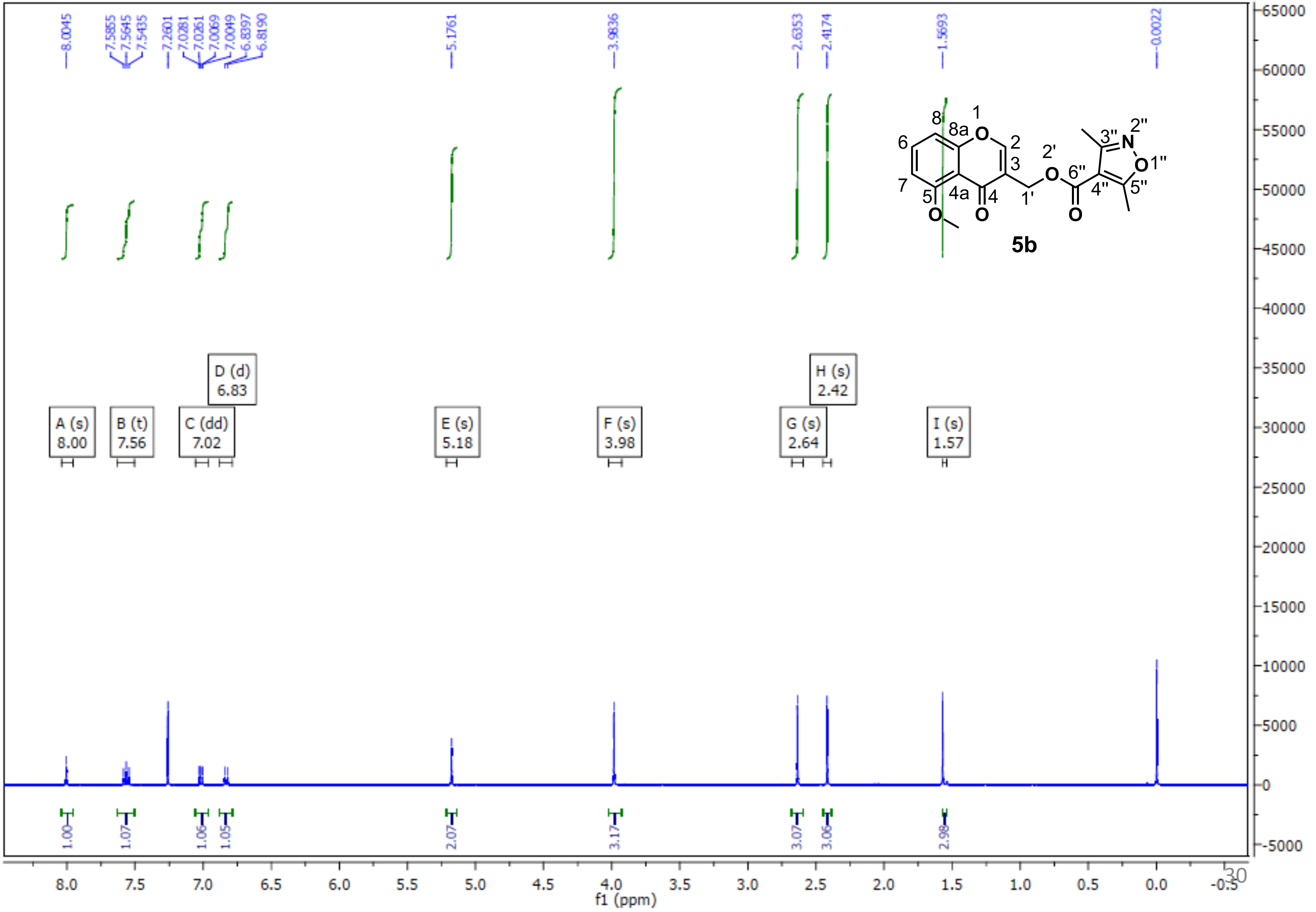

Figure S14.2. ^{13}C NMR spectra of compound 5b

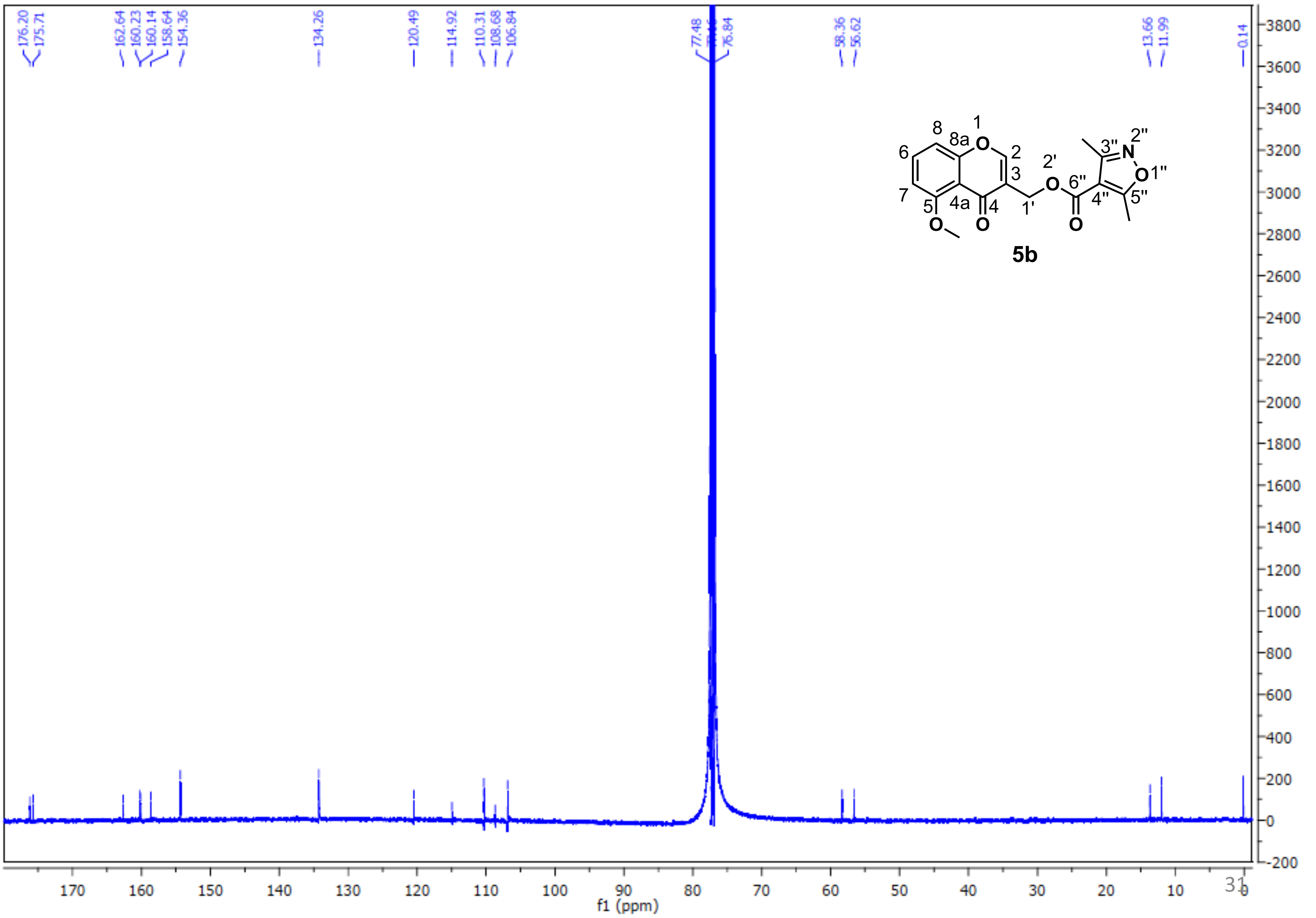

Figure S14.3. ^{13}C-DEPT NMR spectra of compound 5b

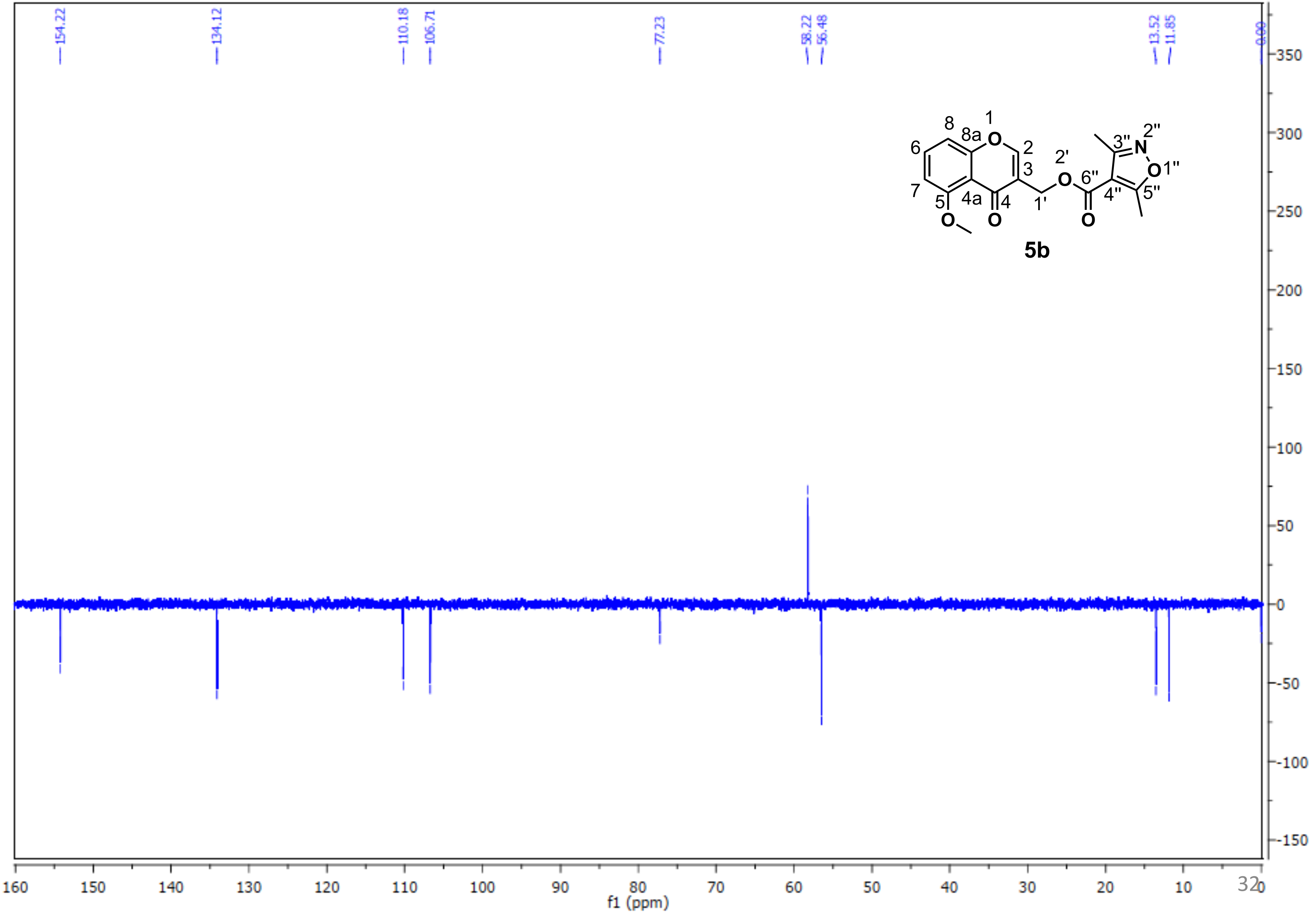

Figure S15.1. ^{1}H NMR spectra of compound 5c

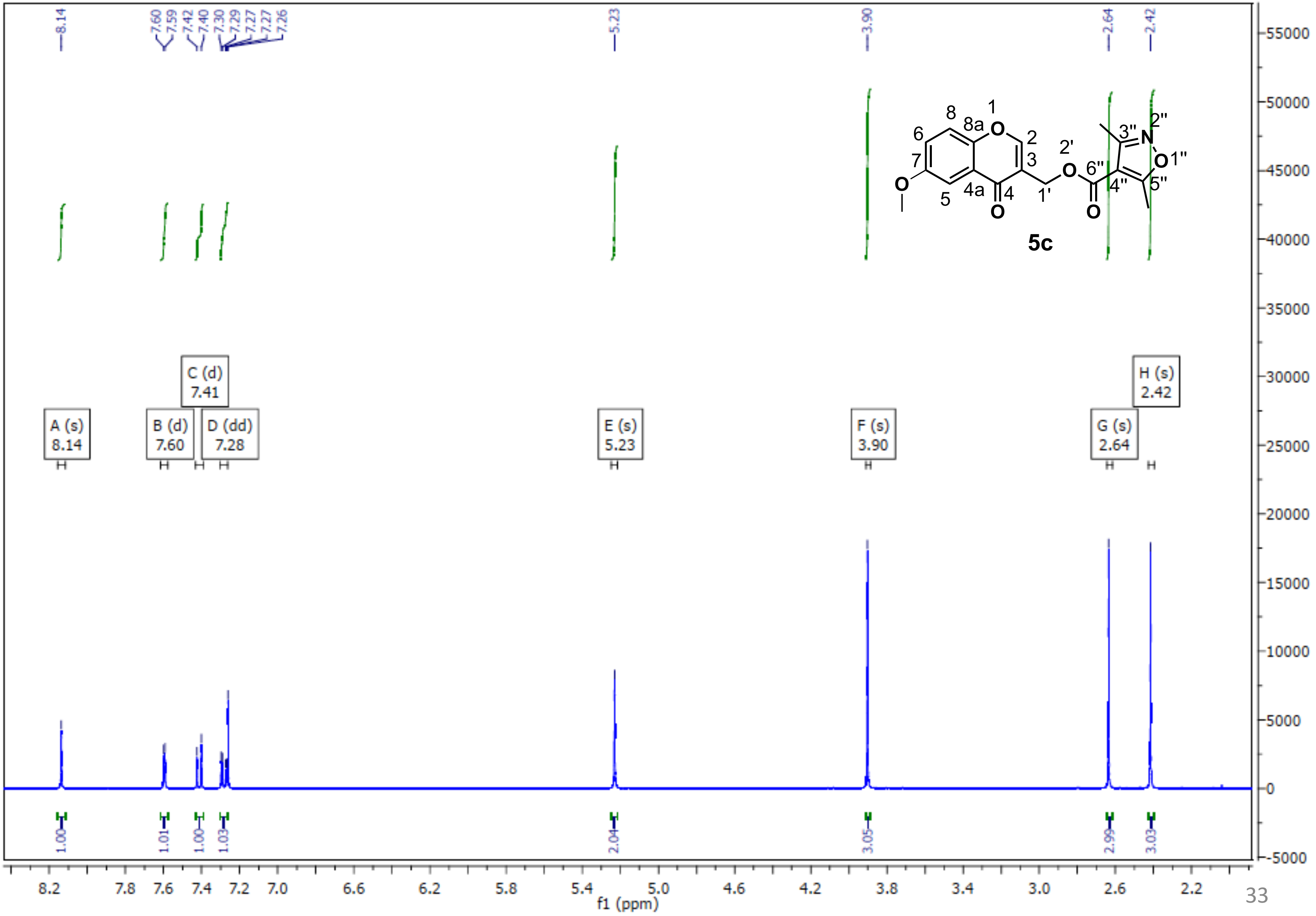

Figure S15.2. ^{13}C NMR spectra of compound 5c

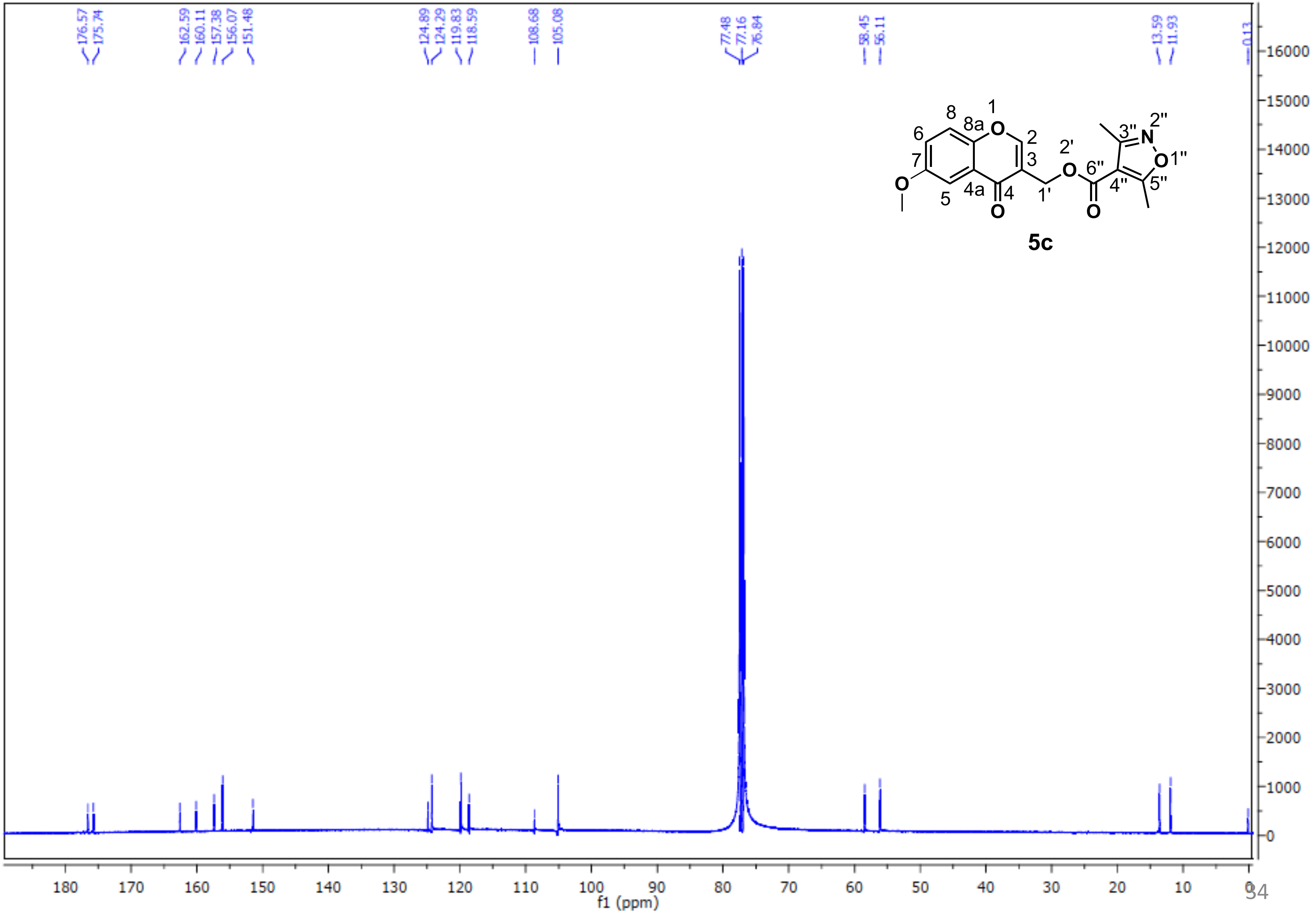

Figure S15.3. ^{13}C-DEPT NMR spectra of compound 5c

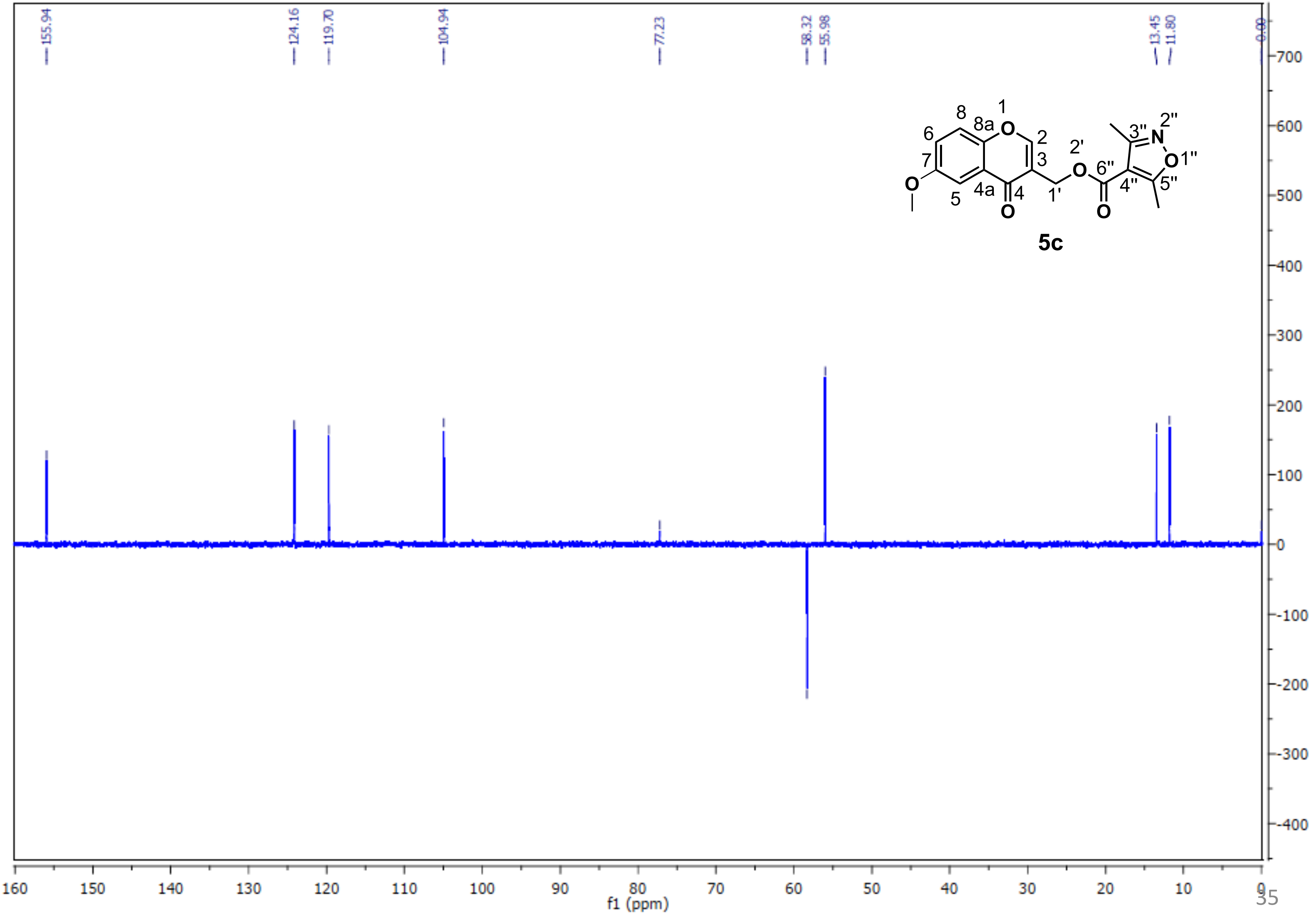

Figure S16.1. ^{1}H NMR spectra of compound 5d

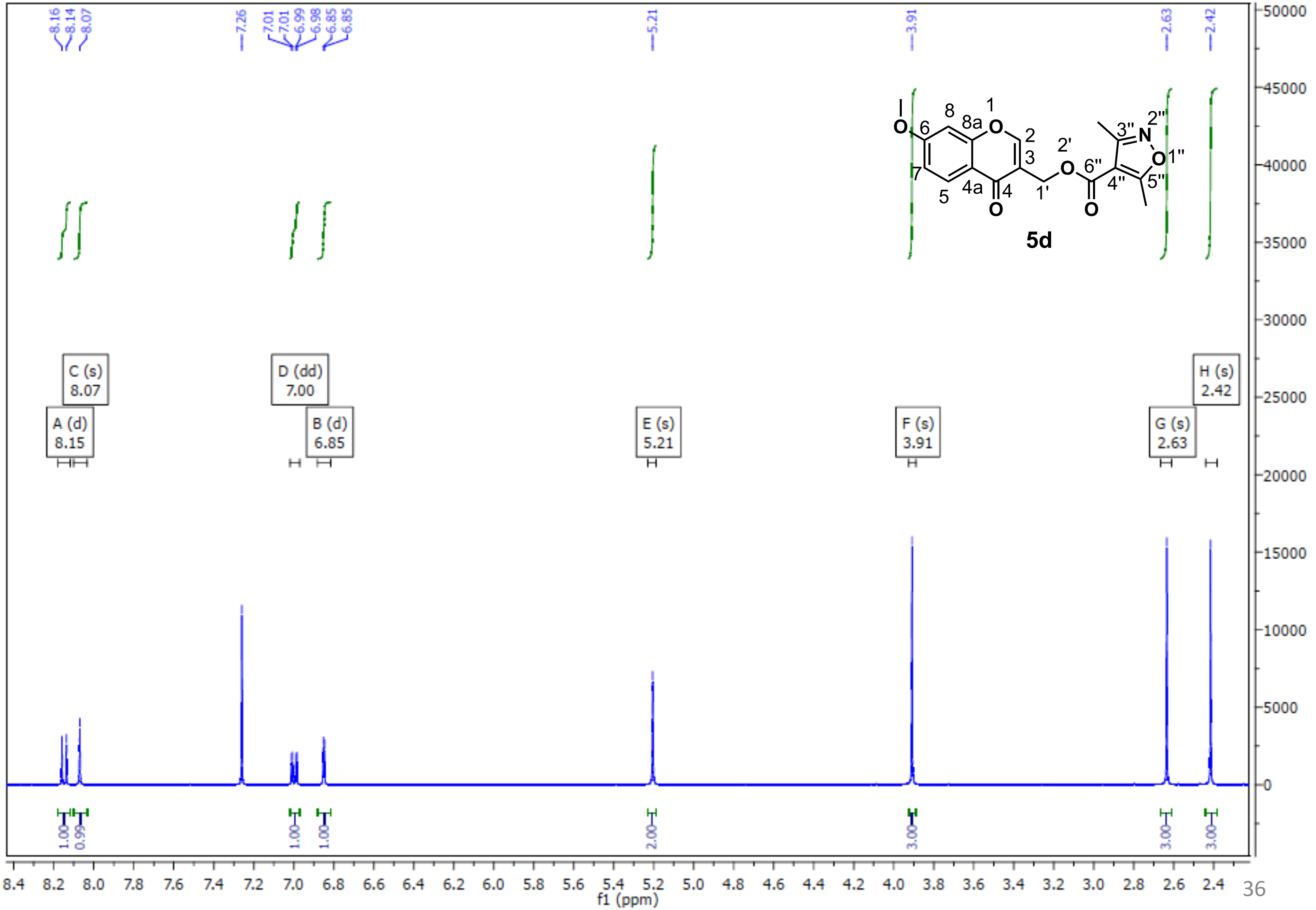

Figure S16.2. ^{13}C NMR spectra of compound 5d

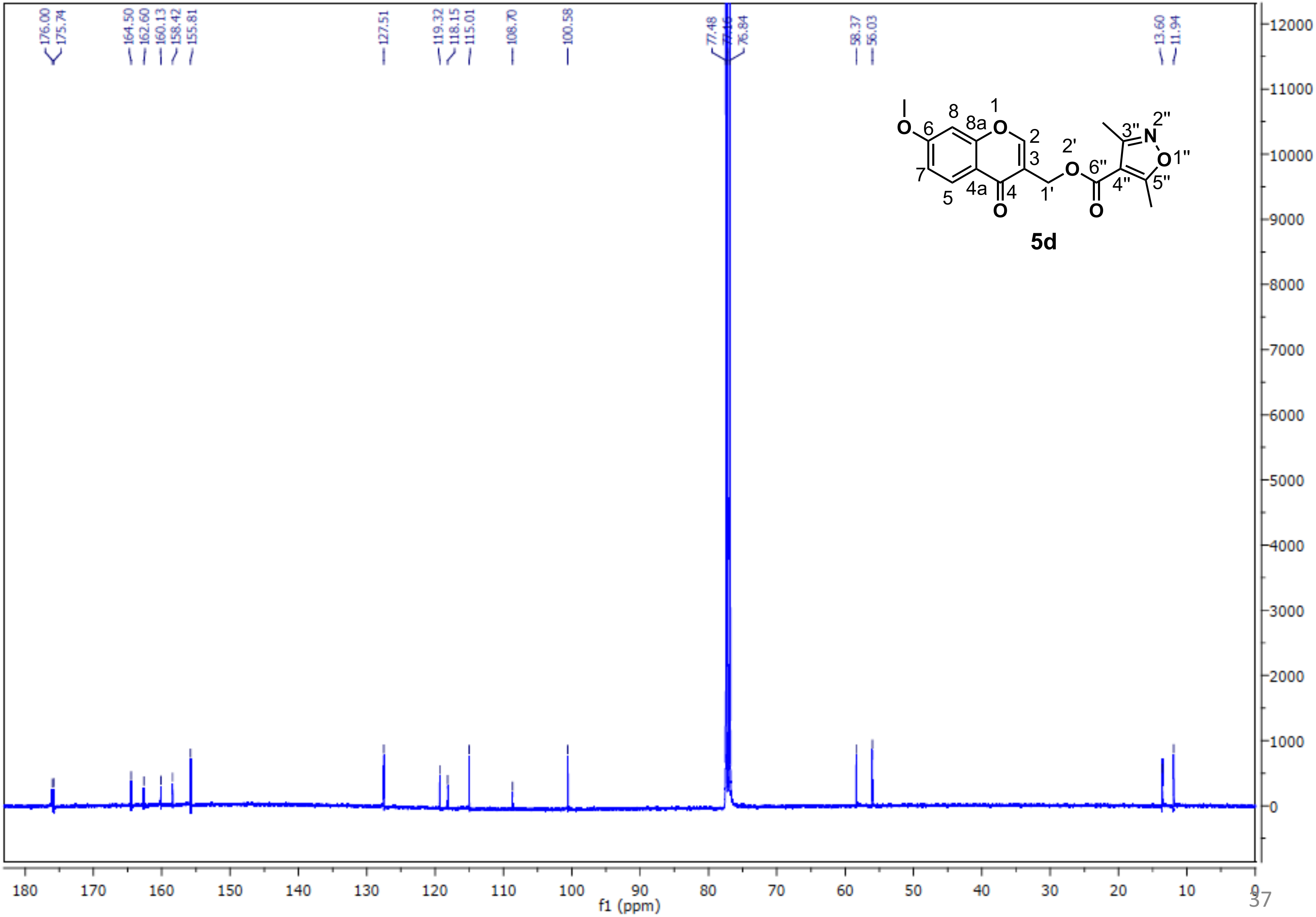

Figure S17.1. ^{1}H NMR spectra of compound 6a

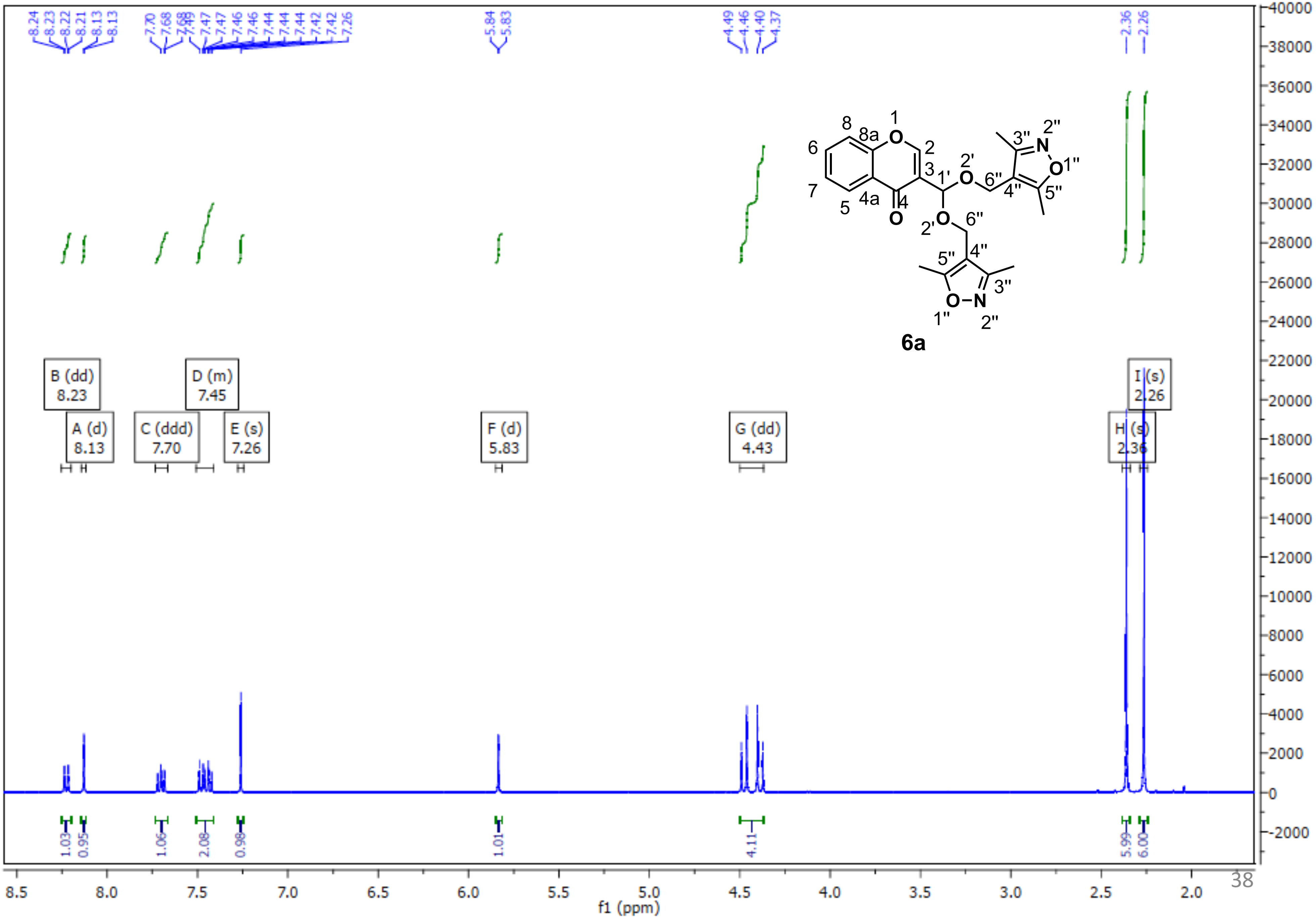

Figure S17.2. ^{13}C NMR spectra of compound 6a

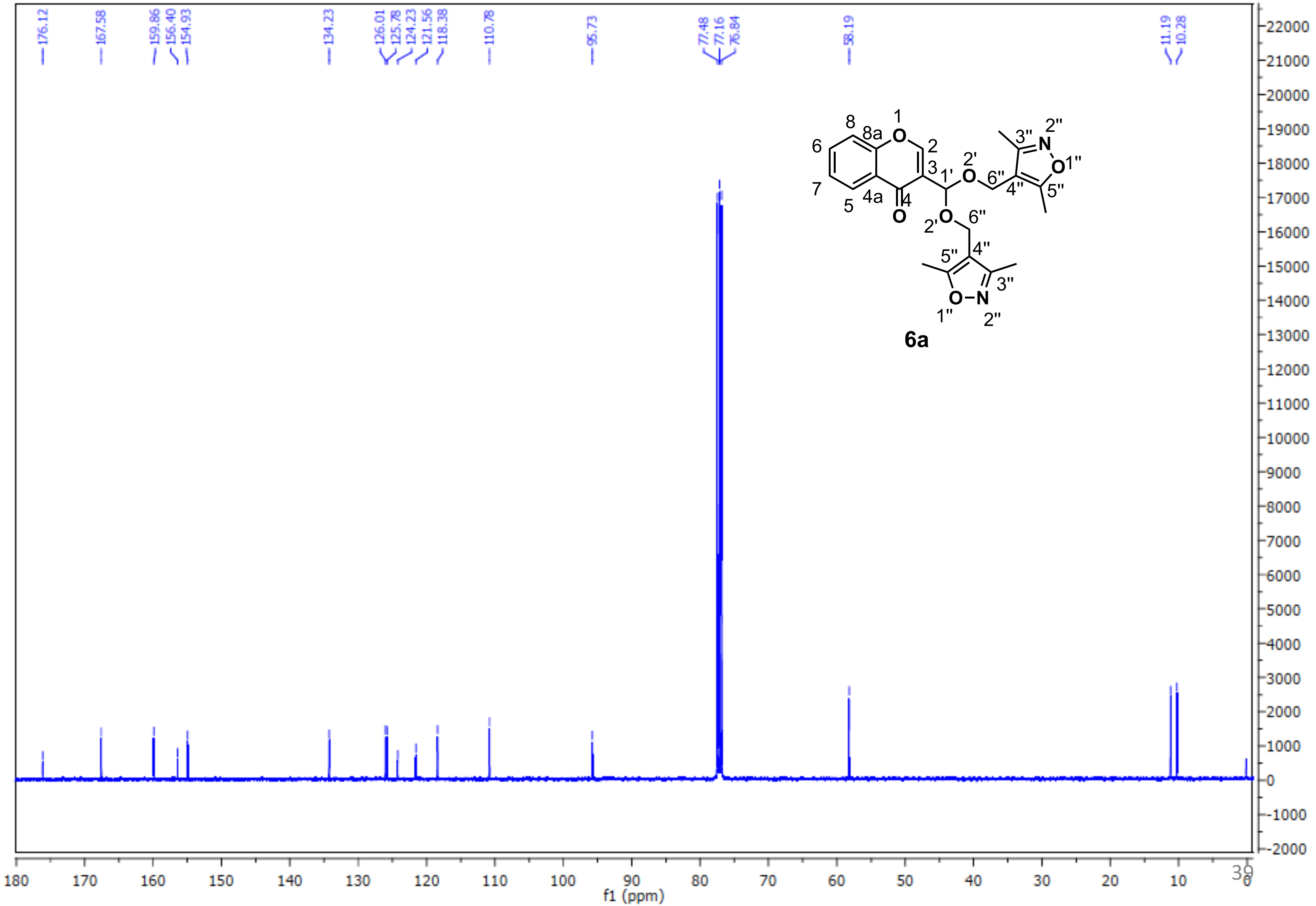

Figure S18.1. ^{1}H NMR spectra of compound 6b

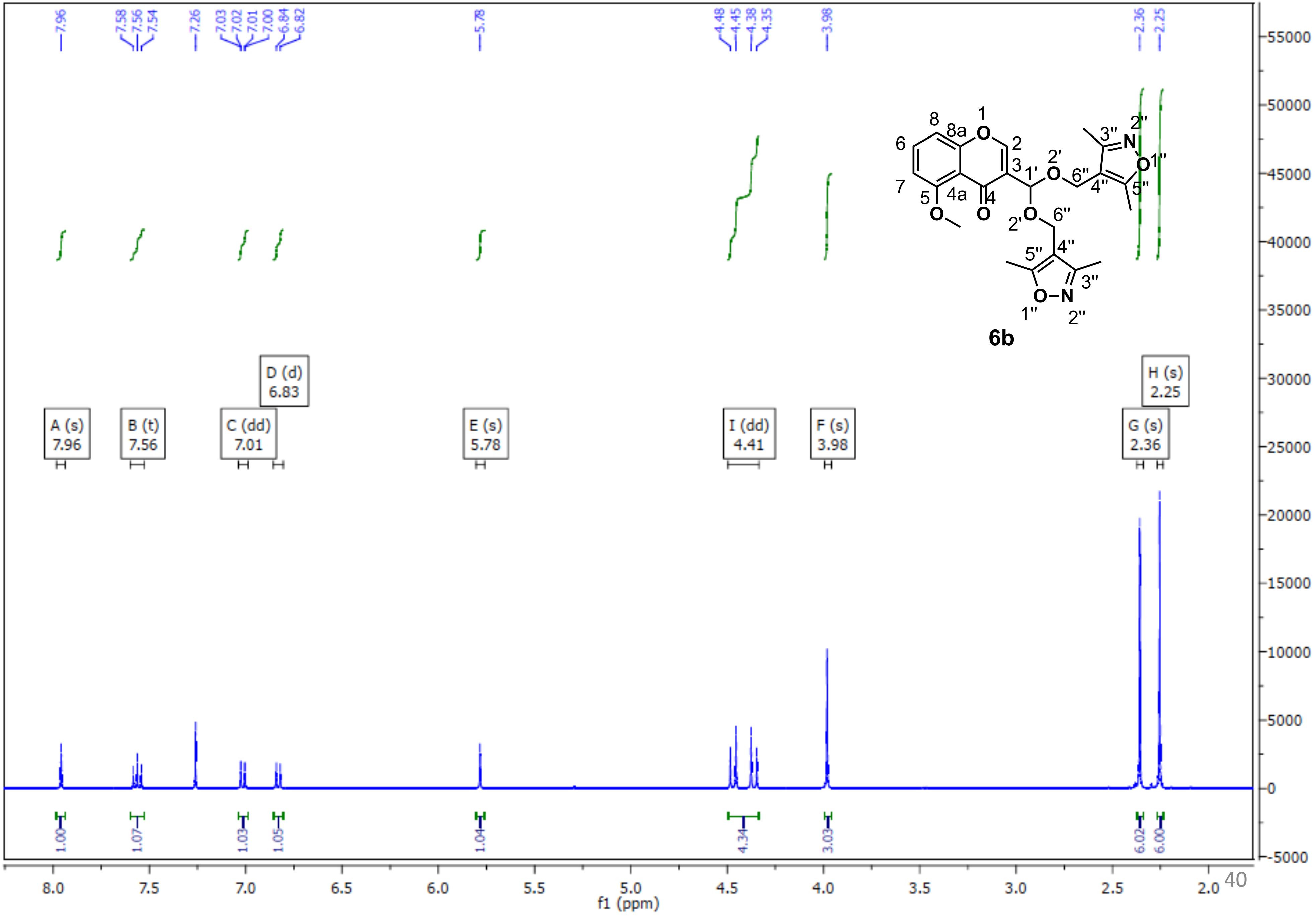

Figure S18.2. ^{13}C NMR spectra of compound 6b

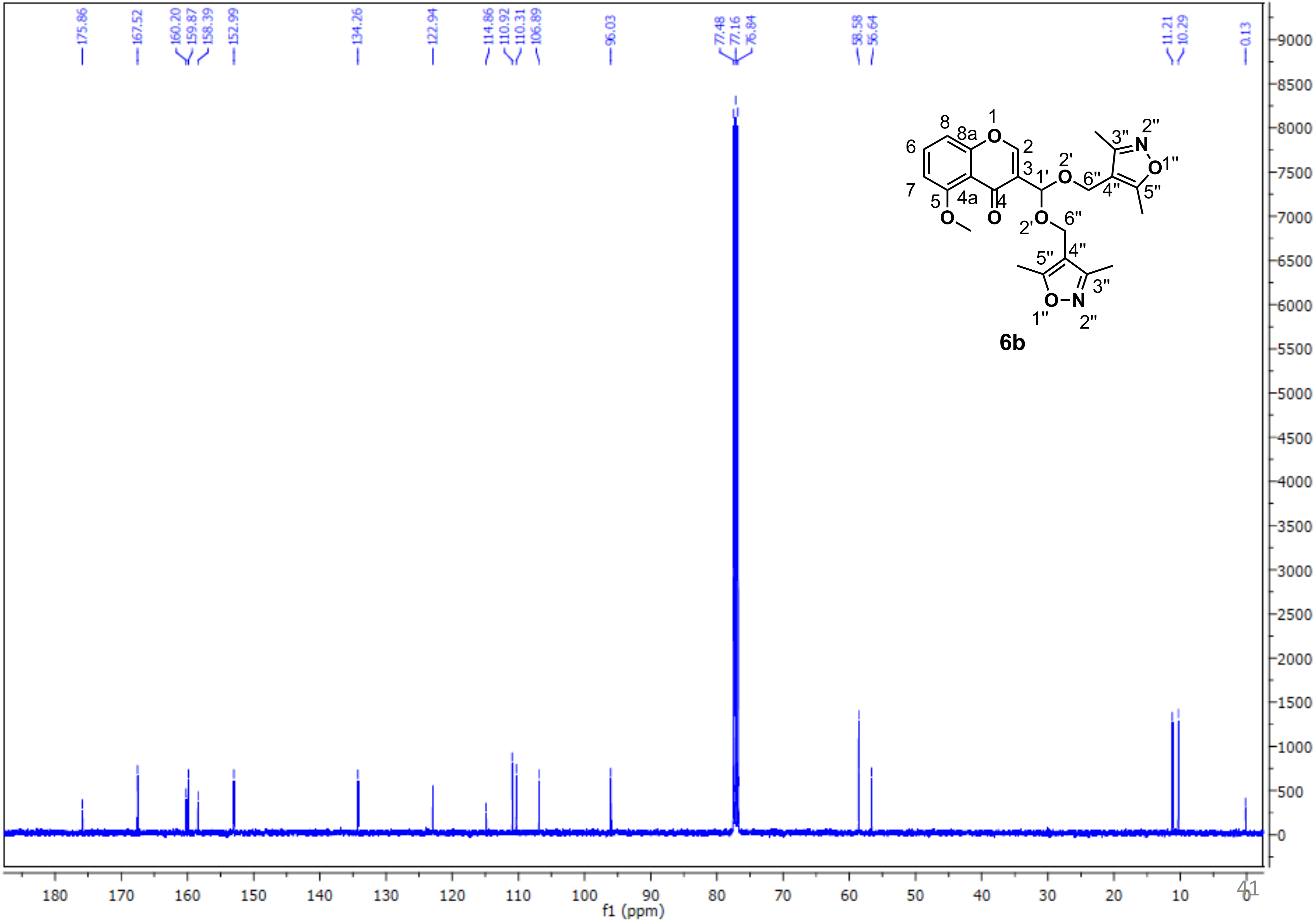

Figure S18.3. ^{13}C-DEPT NMR spectra of compound 6b

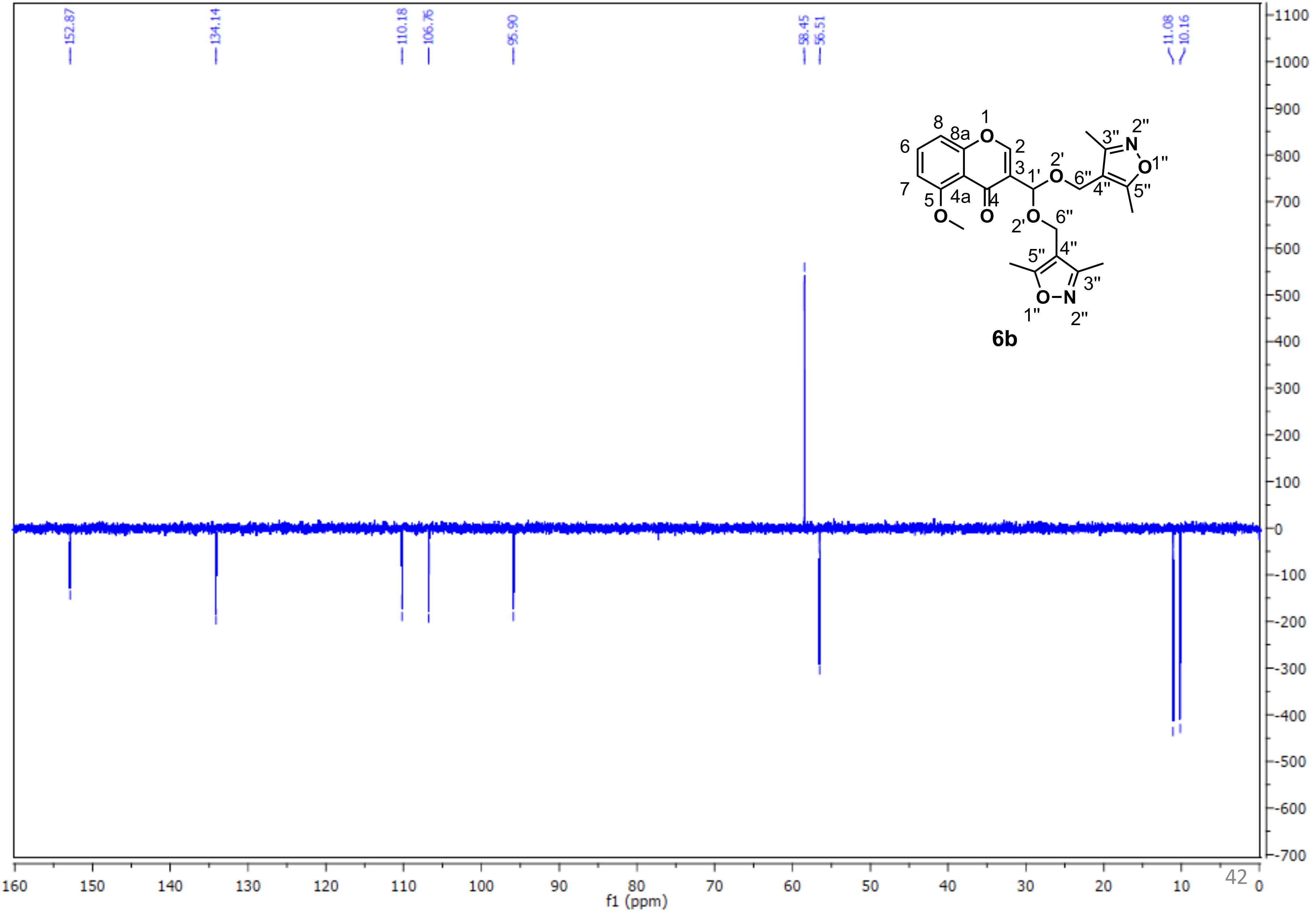

Figure S19.1. ^{1}H NMR spectra of compound 6d

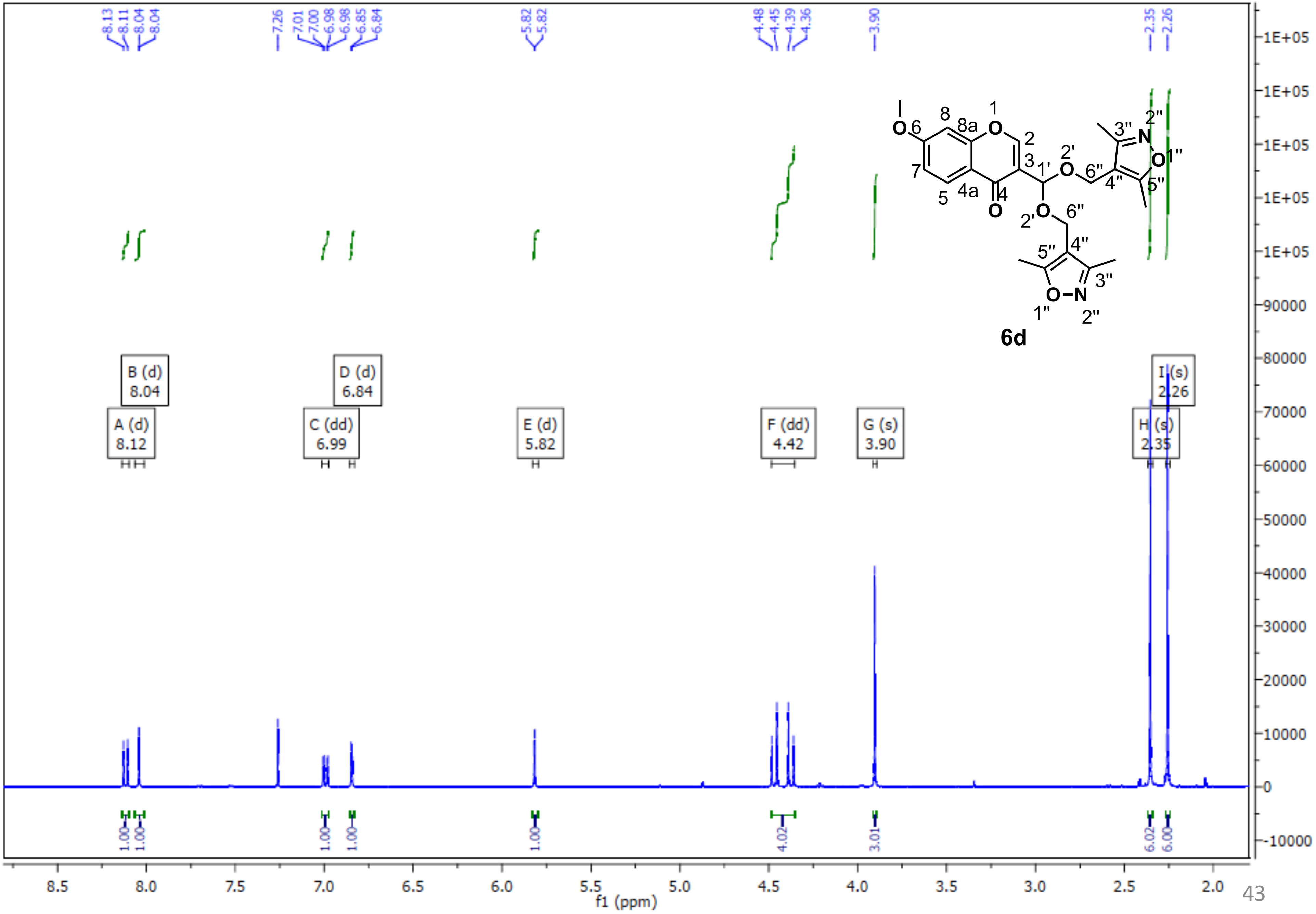

Figure S19.2. ^{13}C NMR spectra of compound 6d

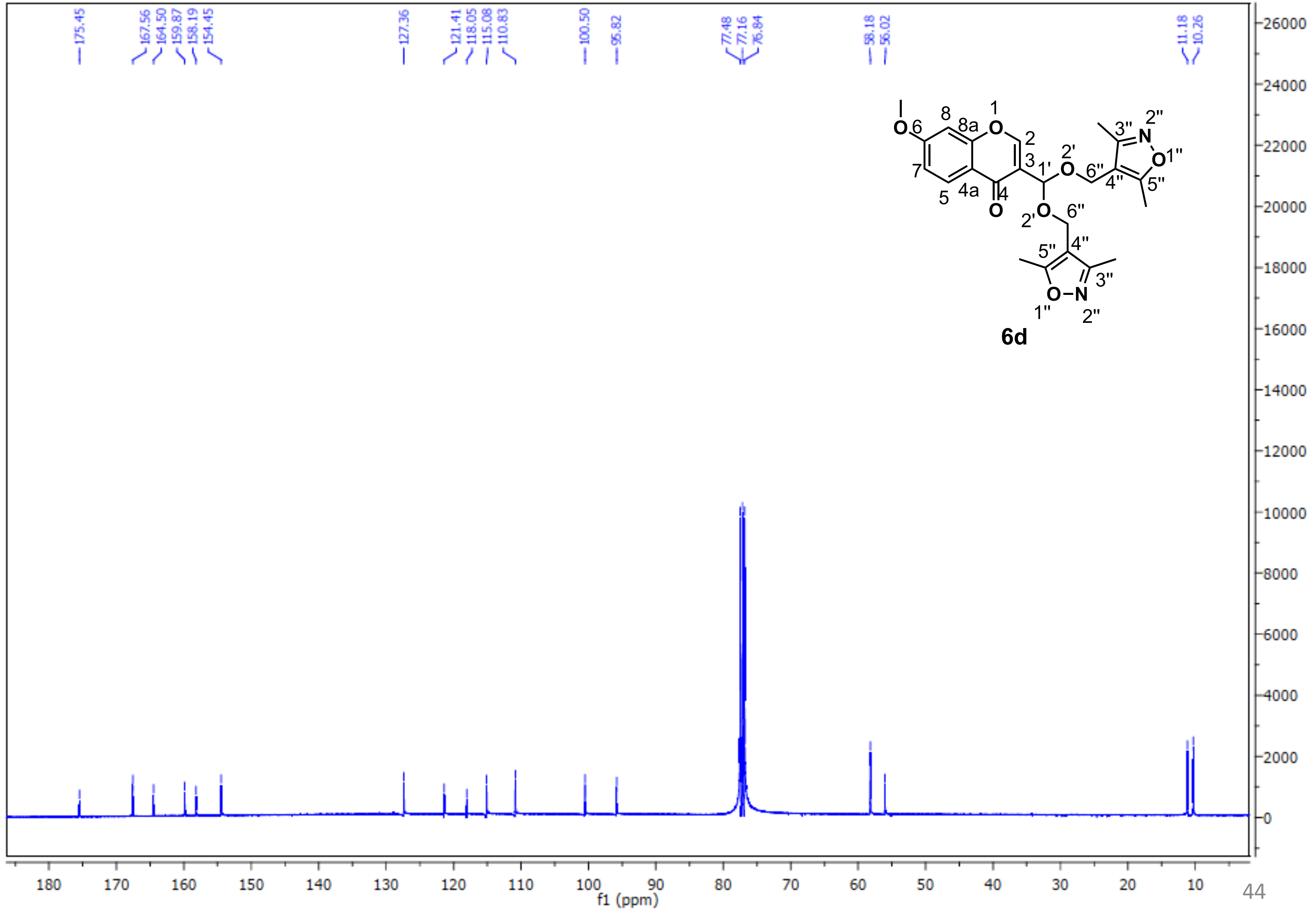

Figure S19.3. ^{13}C-DEPT NMR spectra of compound 6d

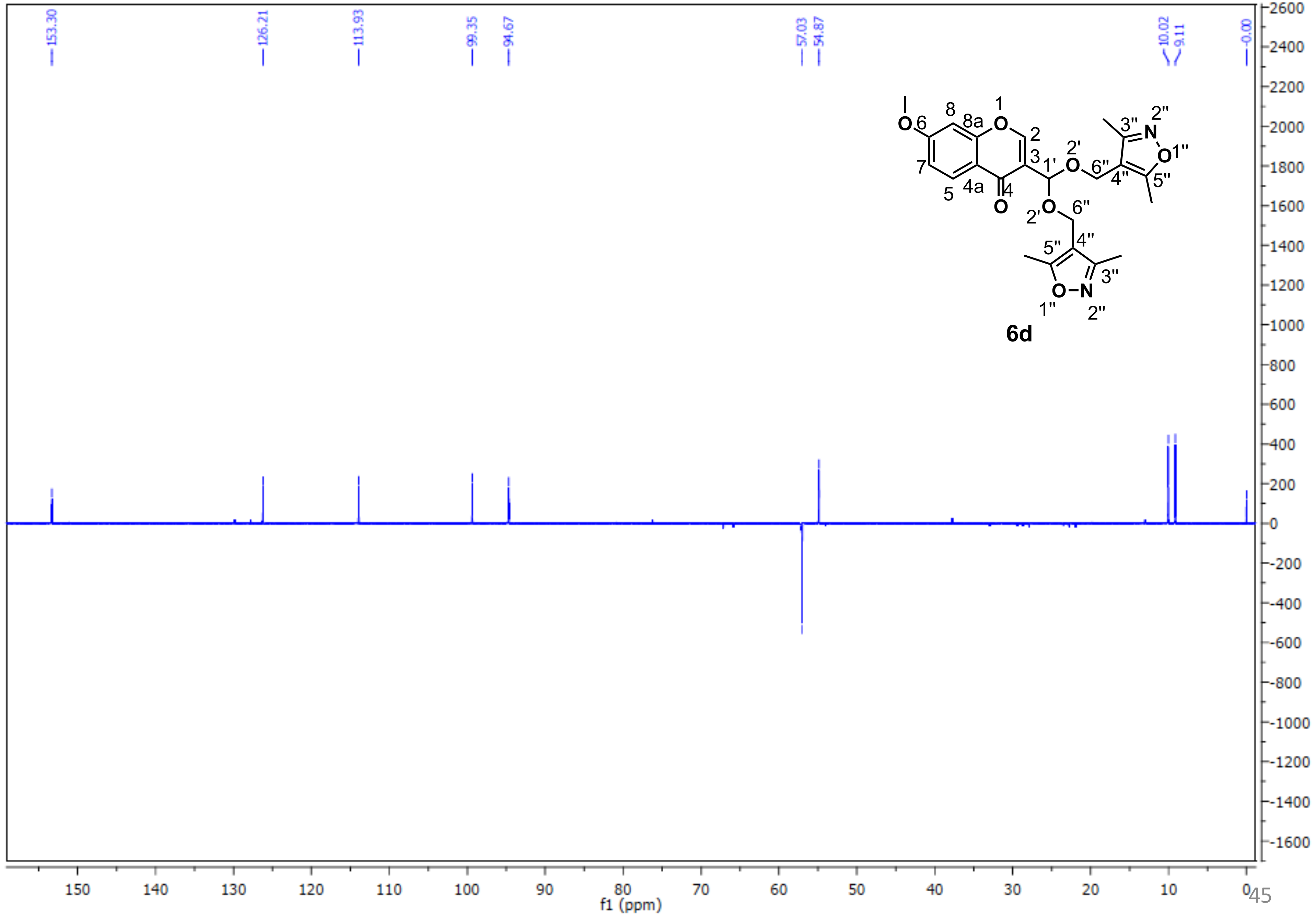

Figure S20.1. ^{1}H NMR spectra of compound 7a

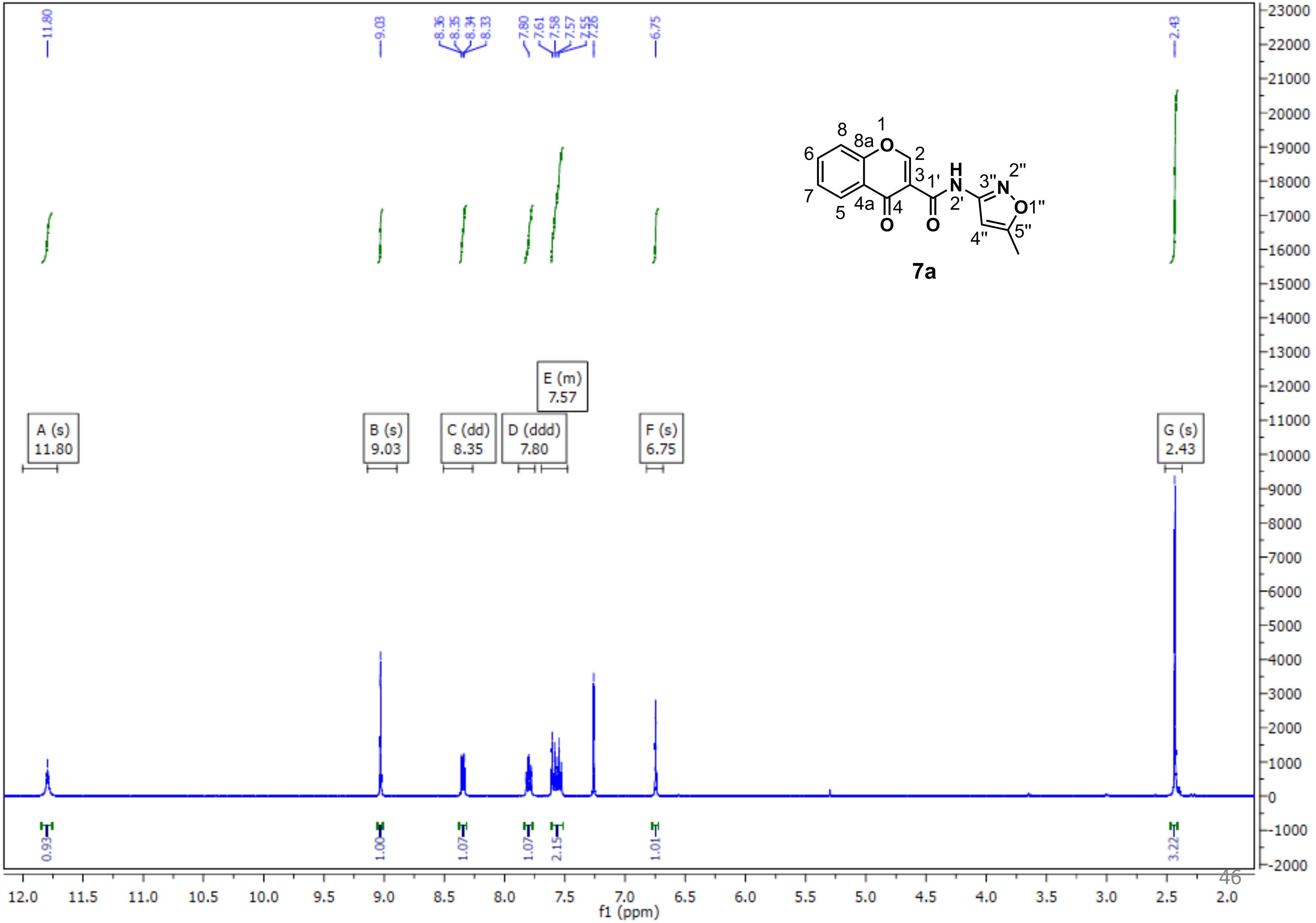

Figure S20.2. ^{13}C NMR spectra of compound 7a

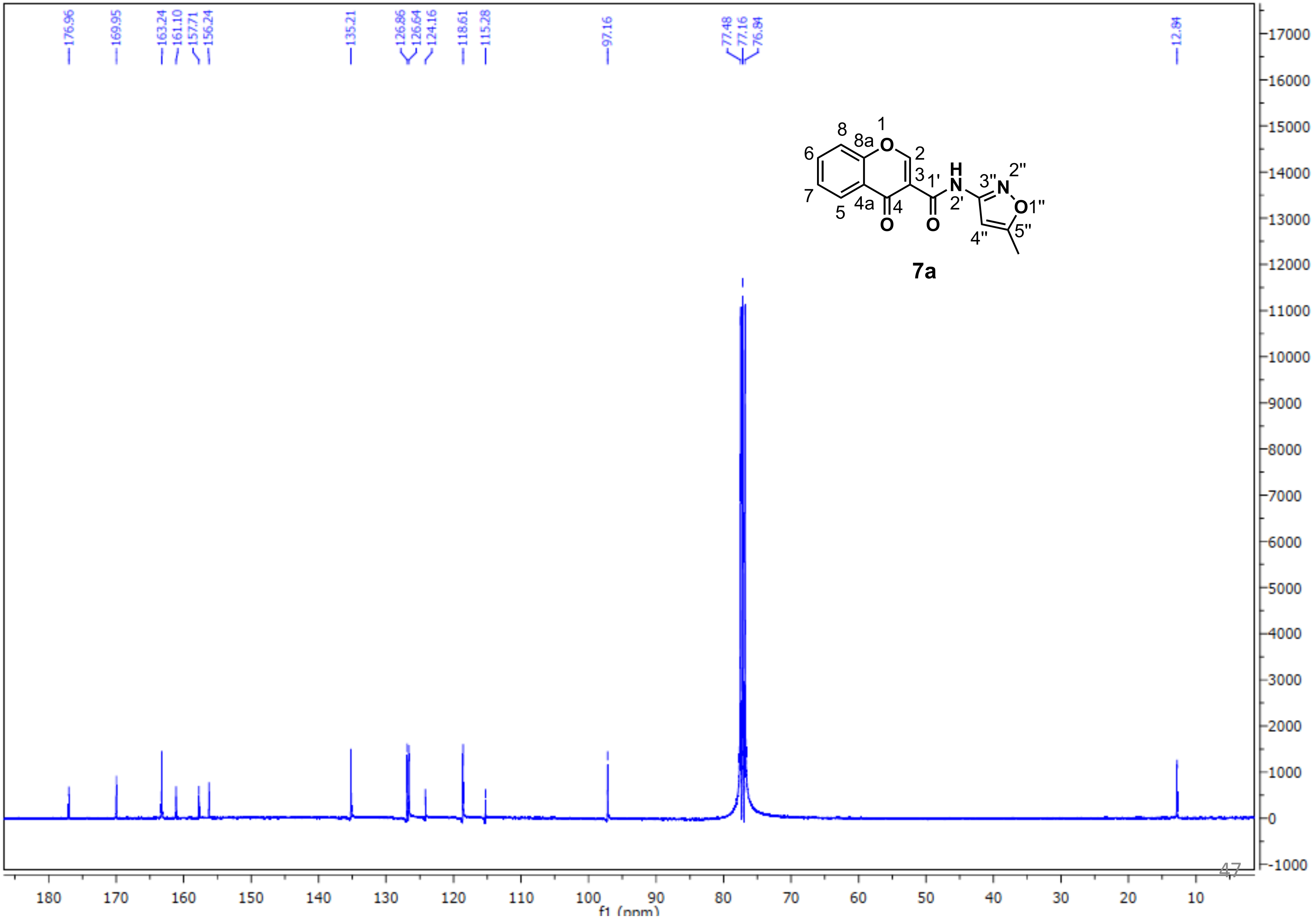

Figure S20.3. ^{13}C-DEPT NMR spectra of compound 7a

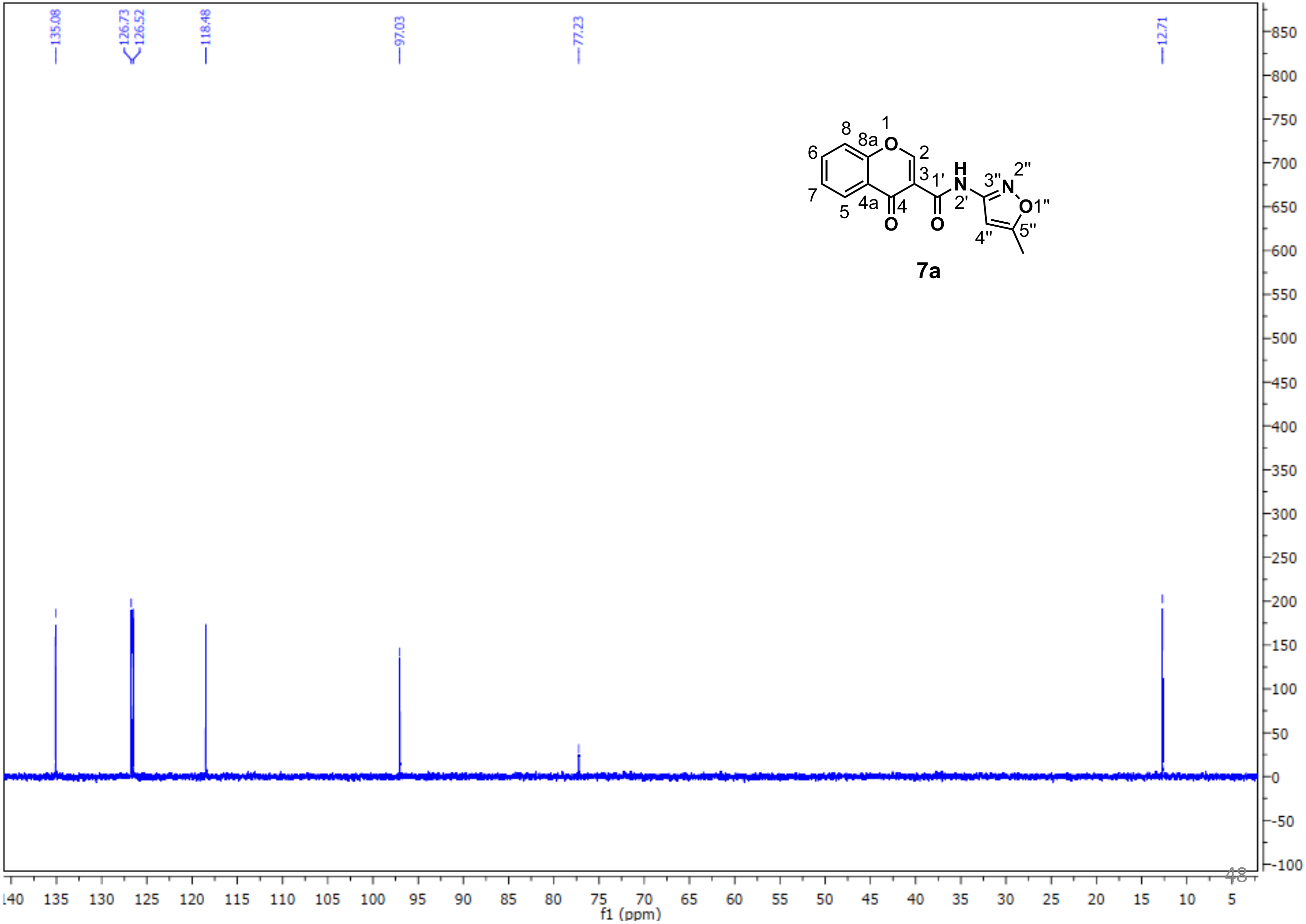

Figure S21.1. ^{1}H NMR spectra of compound 7c

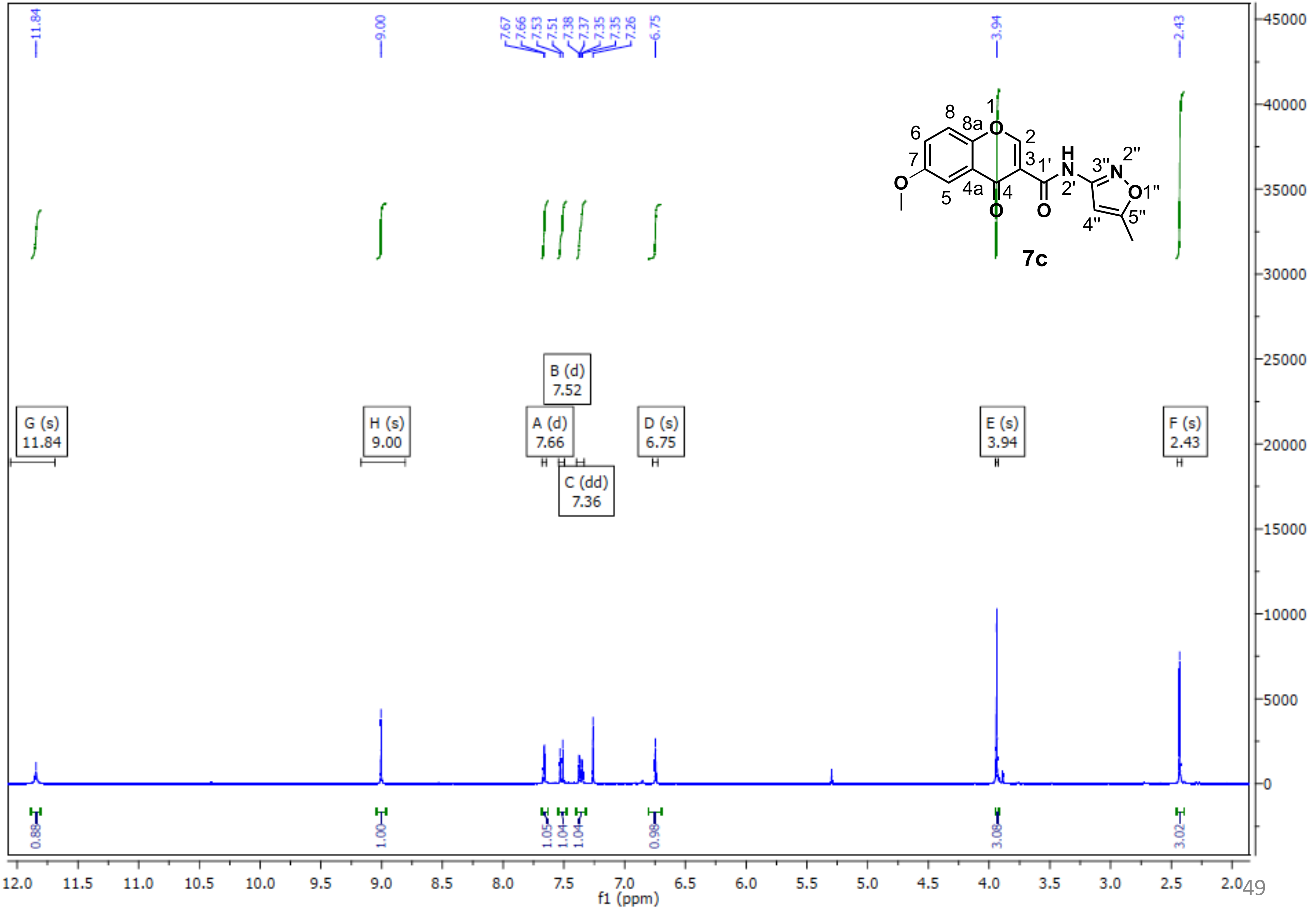

Figure S21.2. ^{13}C NMR spectra of compound 7c

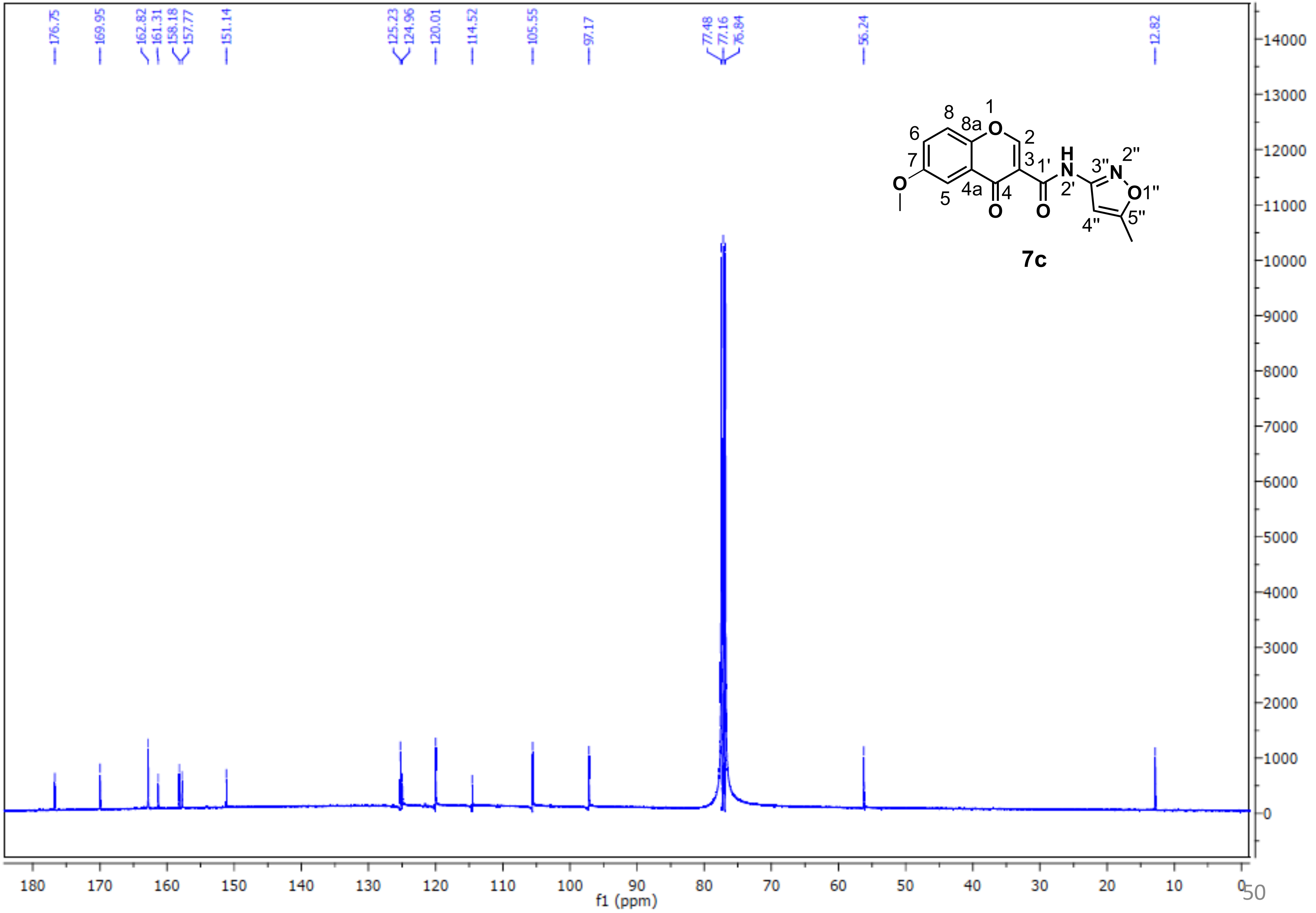

Figure S21.3. ^{13}C-DEPT NMR spectra of compound 7c

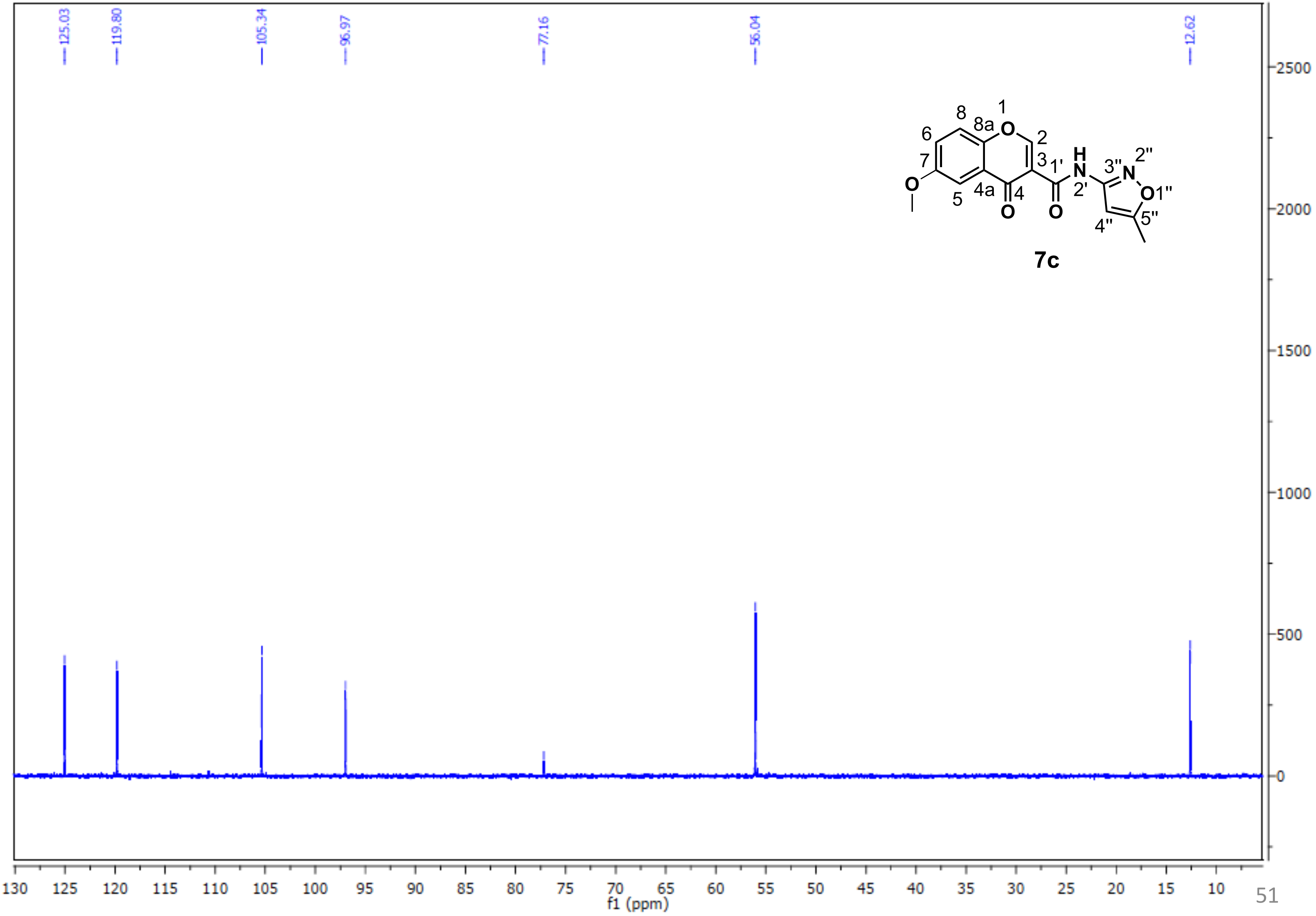

Figure S22.1. ^{1}H NMR spectra of compound 7d

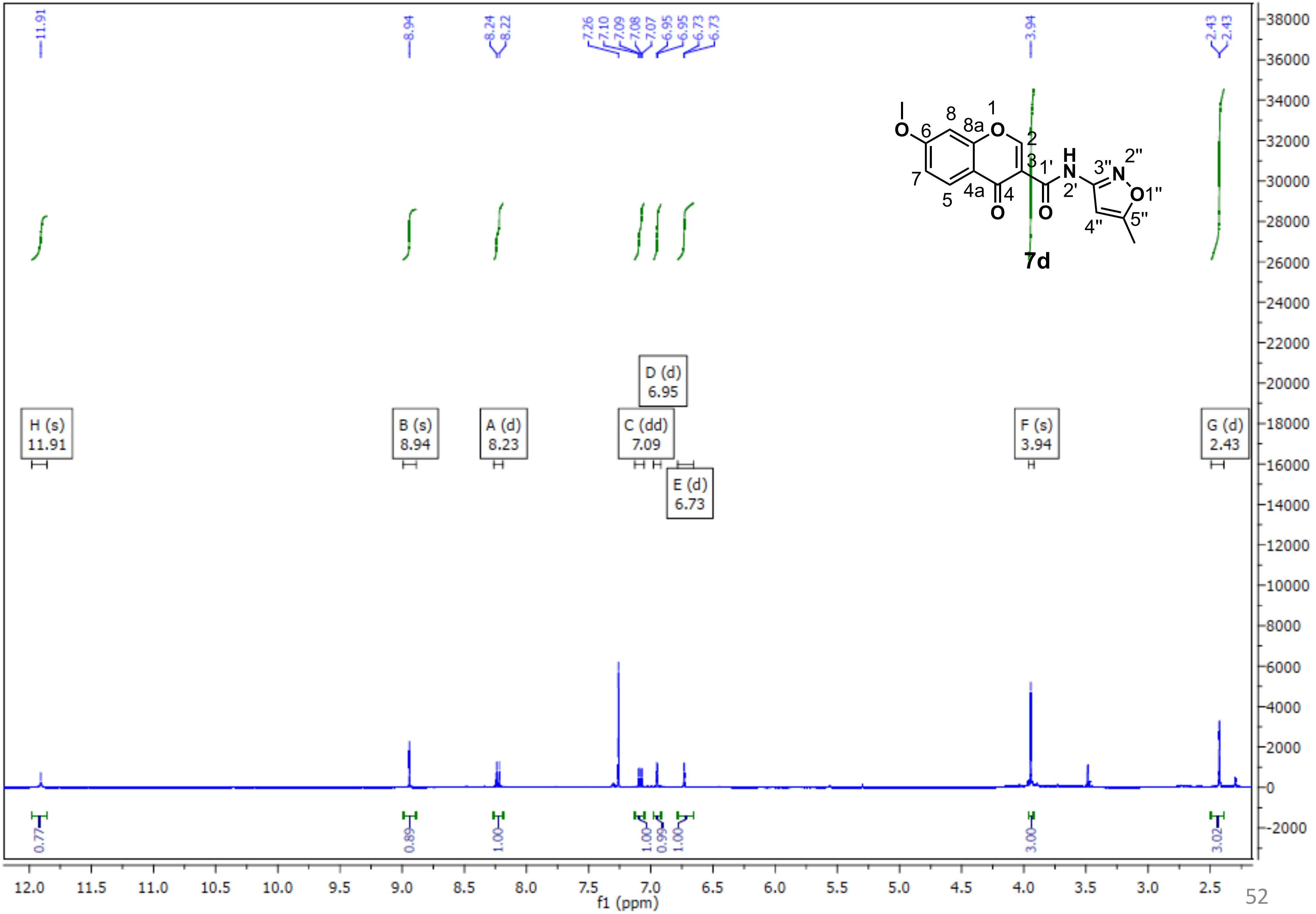

Figure S22.2. ^{13}C NMR spectra of compound 7d

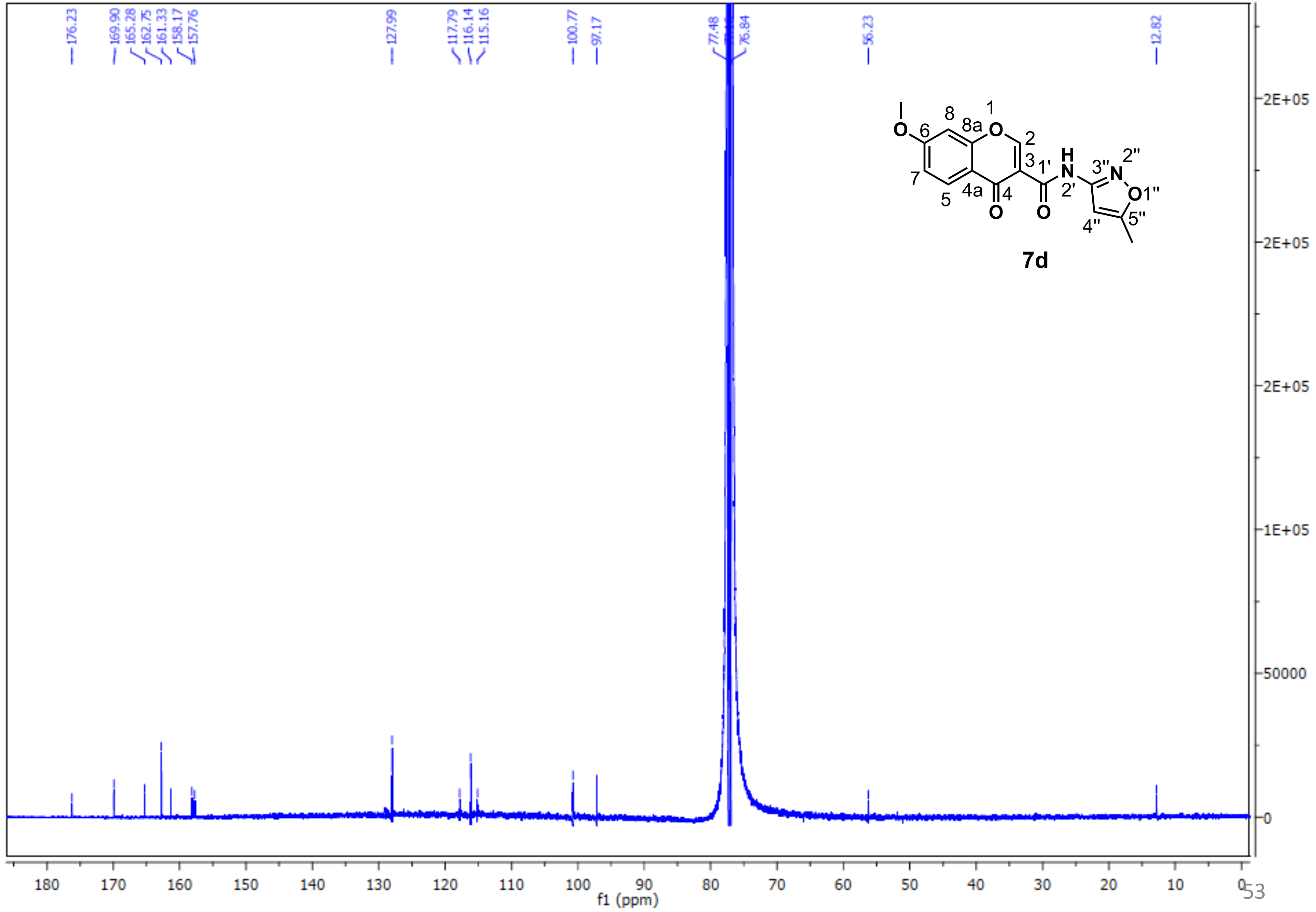

Figure S22.3. ^{13}C-DEPT NMR spectra of compound 7d

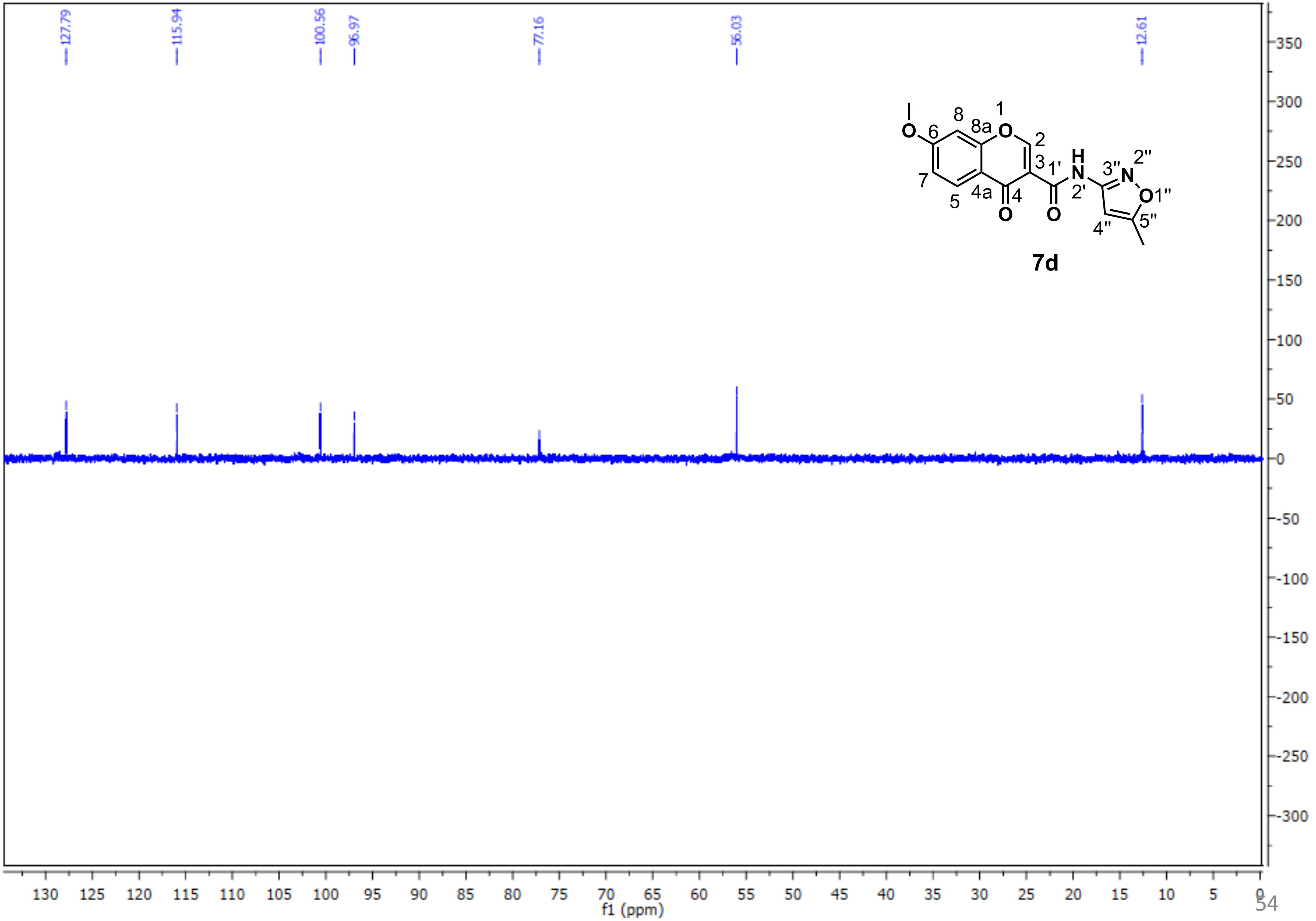

Figure S23.1. ^{1}H NMR spectra of compound 8a

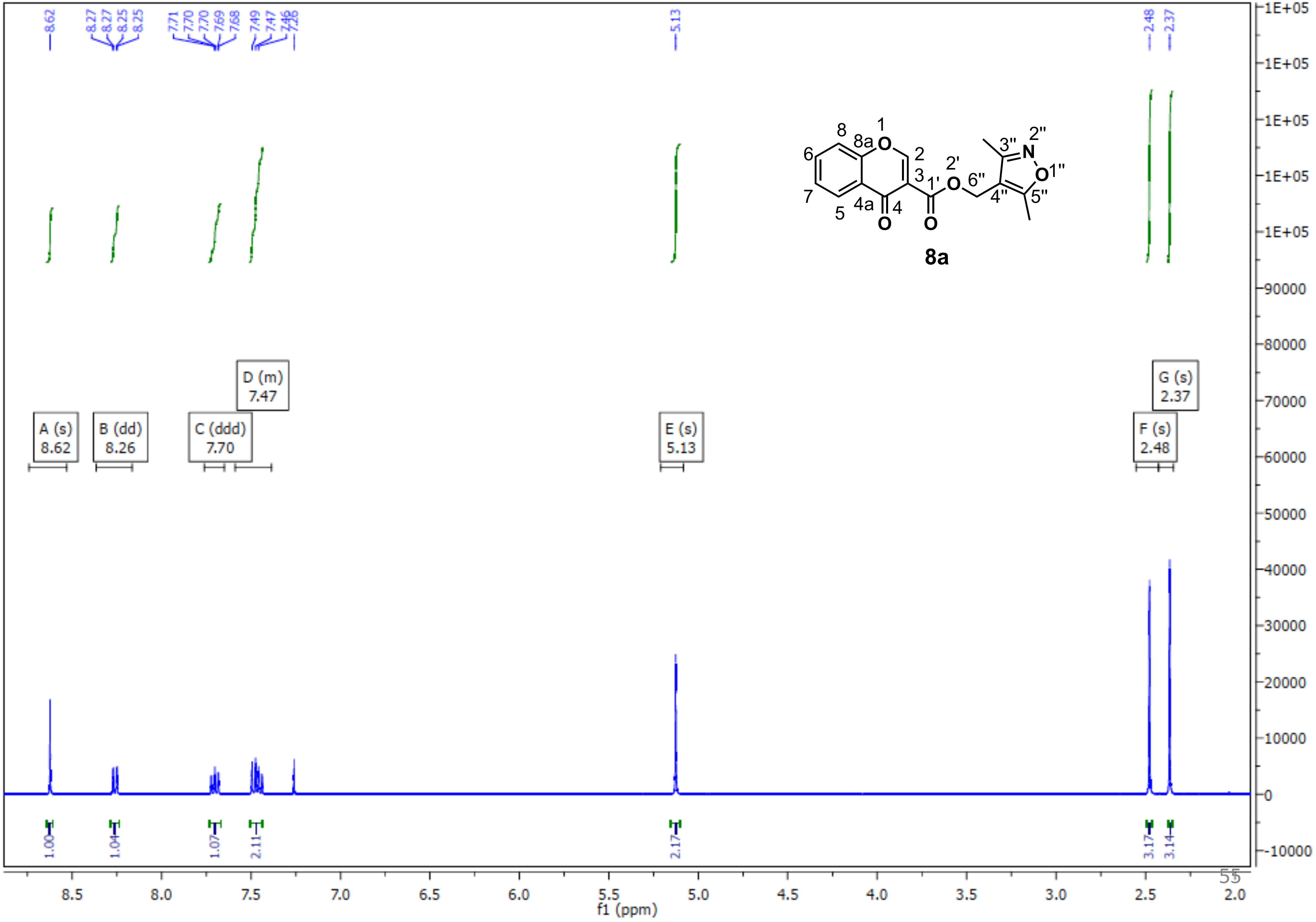

Figure S23.2. ^{13}C NMR spectra of compound 8a

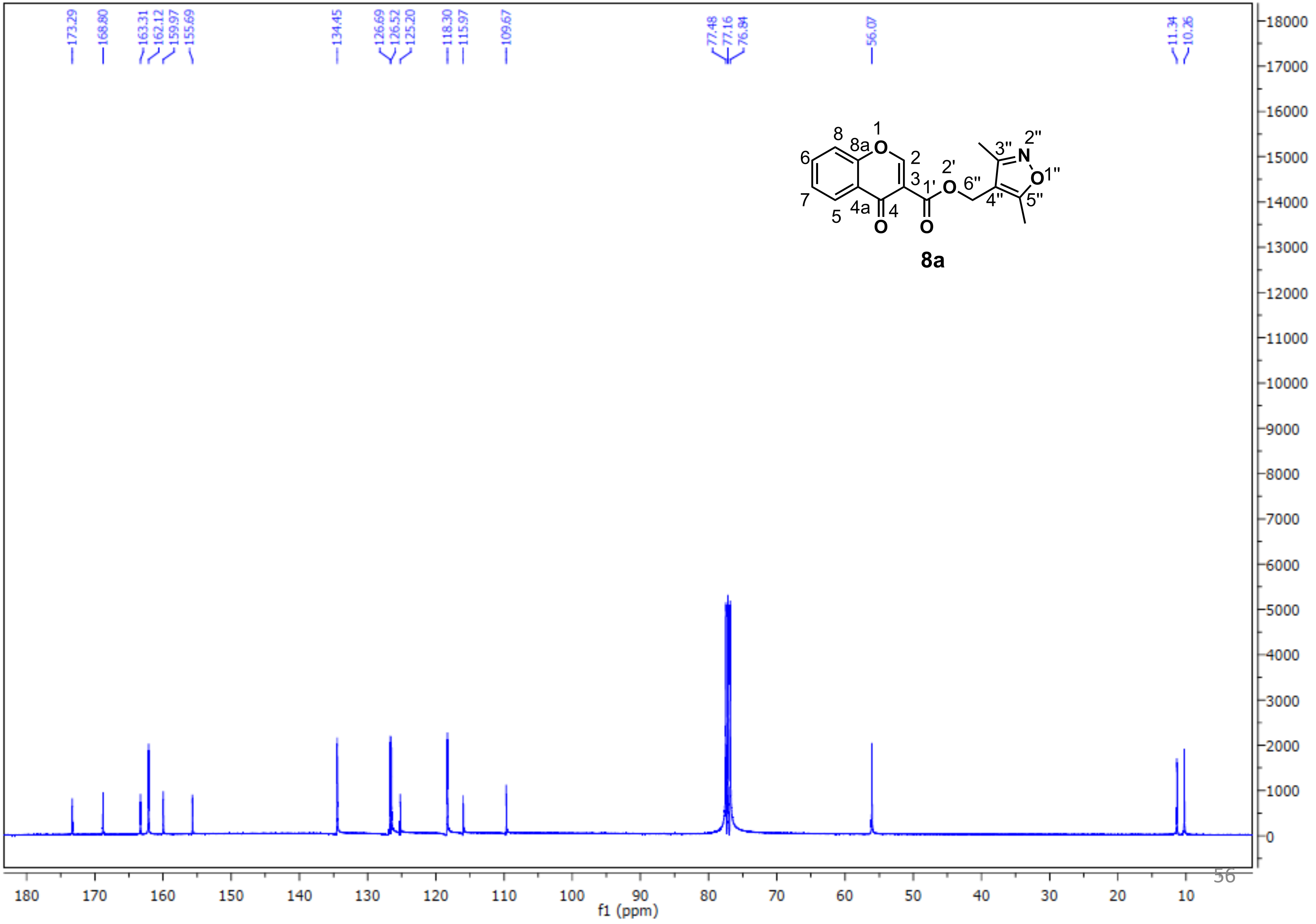

Figure S23.3. ^{13}C-DEPT NMR spectra of compound 8a

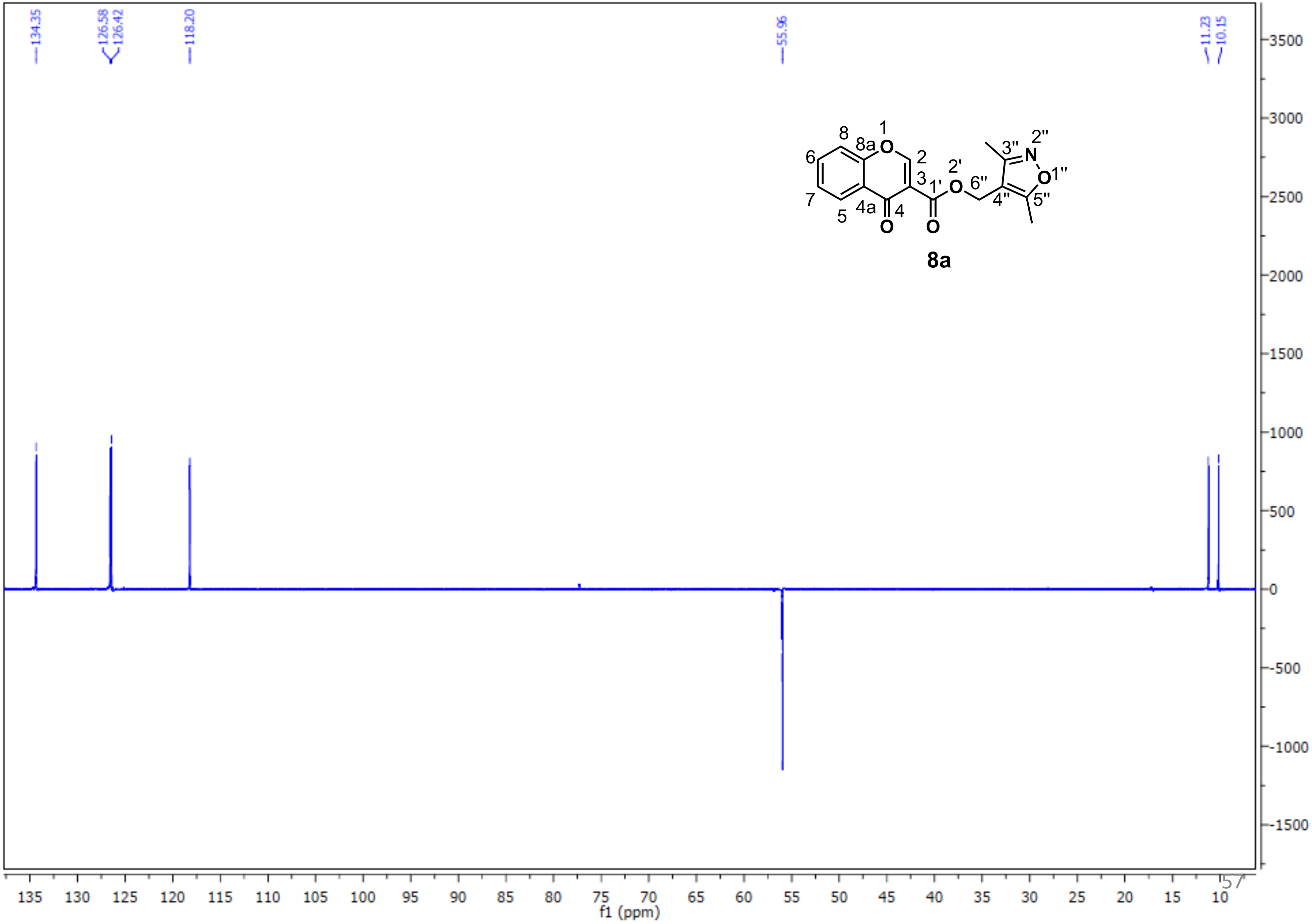

Figure S24.1. ^{1}H NMR spectra of compound 9a

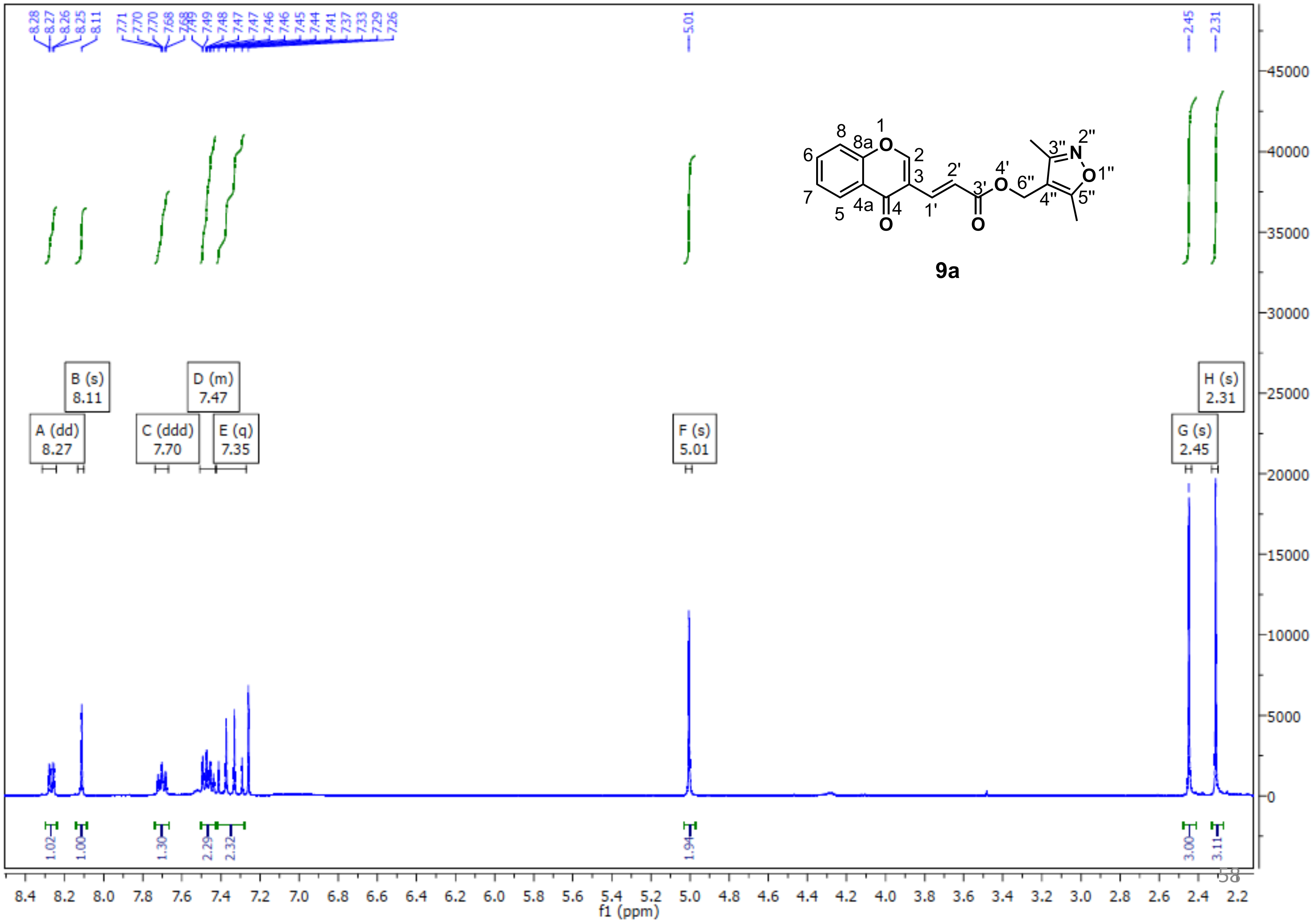

Figure S24.2. ^{13}C NMR spectra of compound 9a

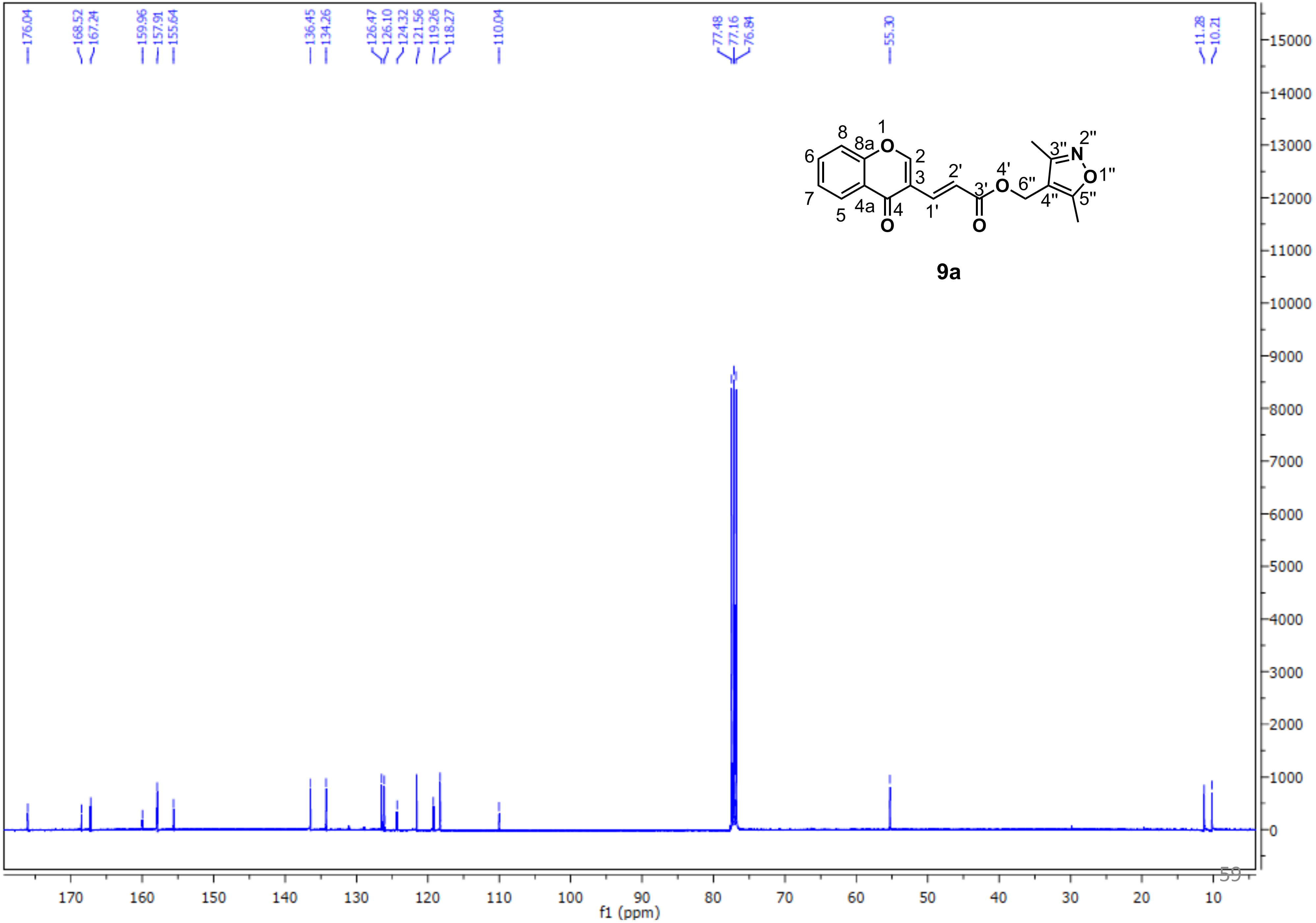

Figure S25.1. ^{1}H NMR spectra of compound 9c

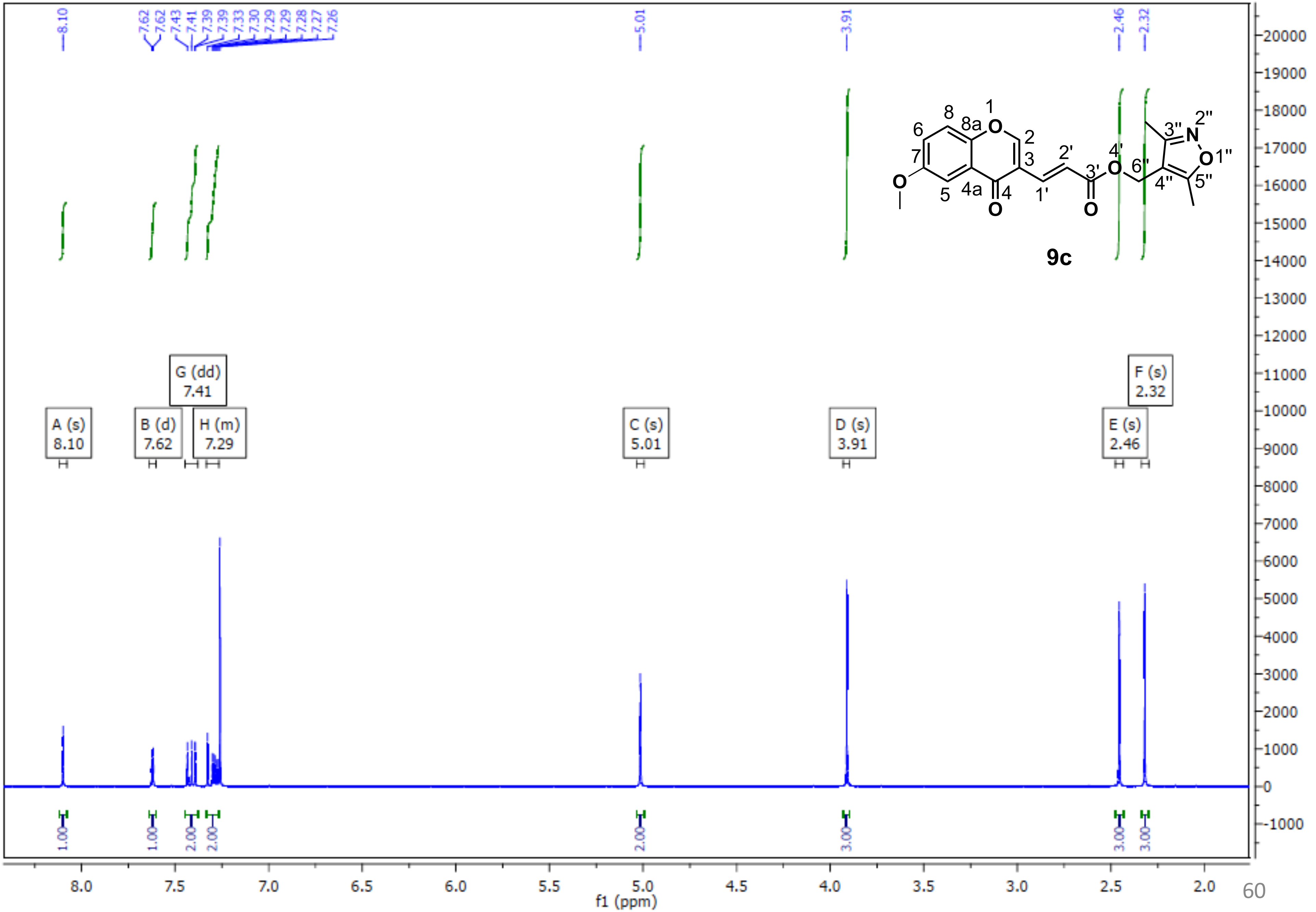

Figure S25.2. ^{13}C NMR spectra of compound 9c

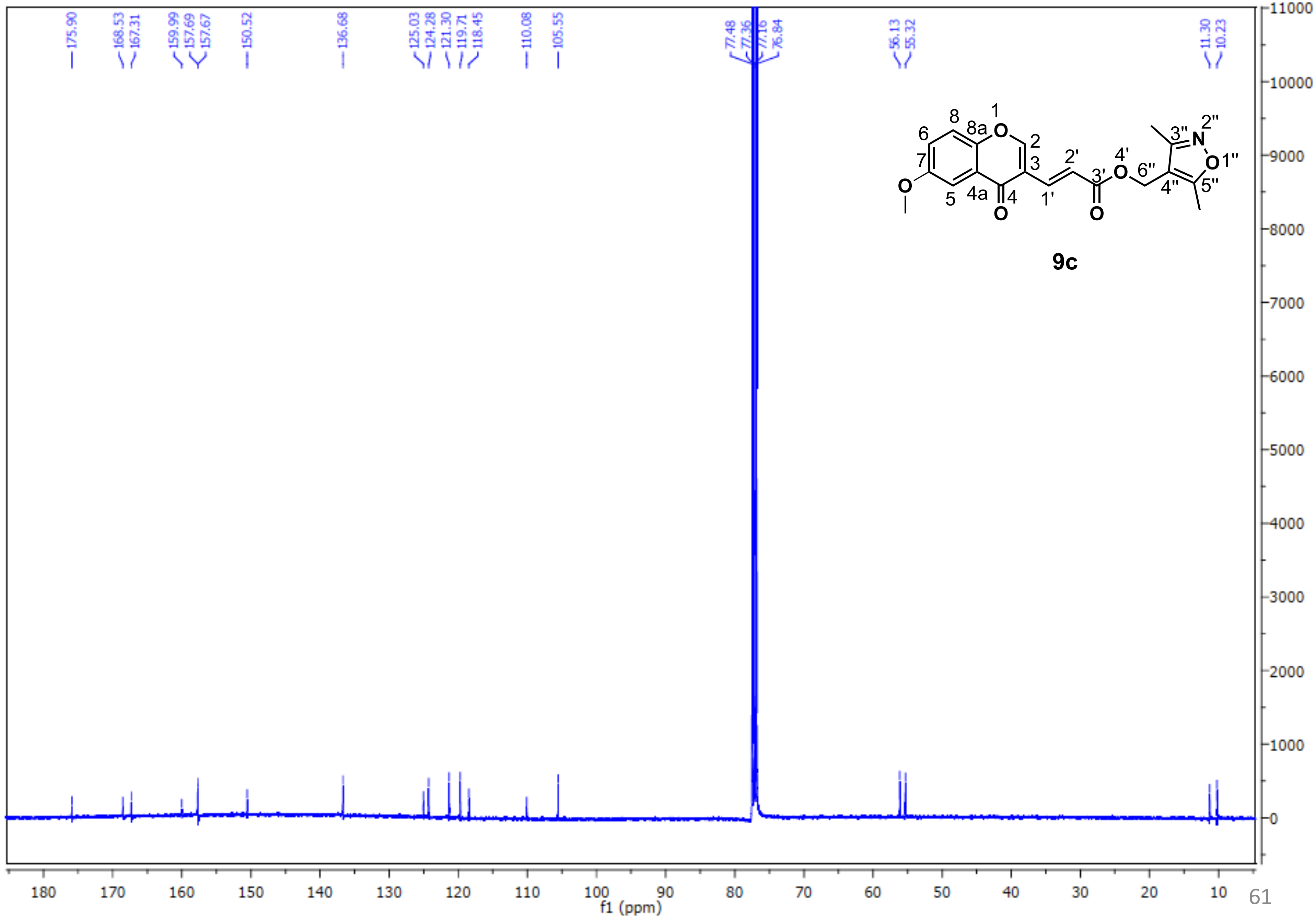

Figure S25.3. ^{13}C-DEPT NMR spectra of compound 9c

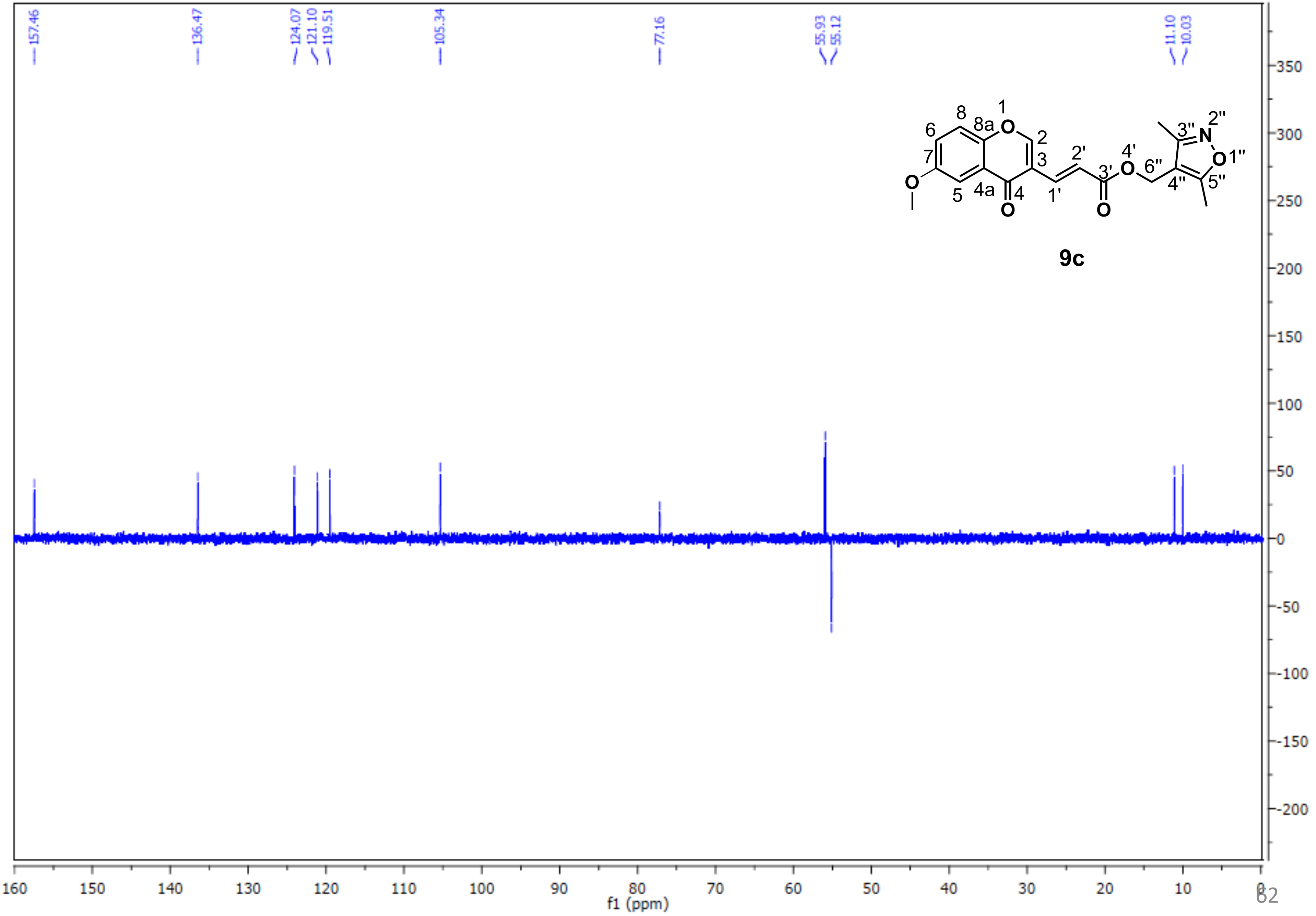

Figure S26.1. ^{1}H **NMR spectra of compound 9d**

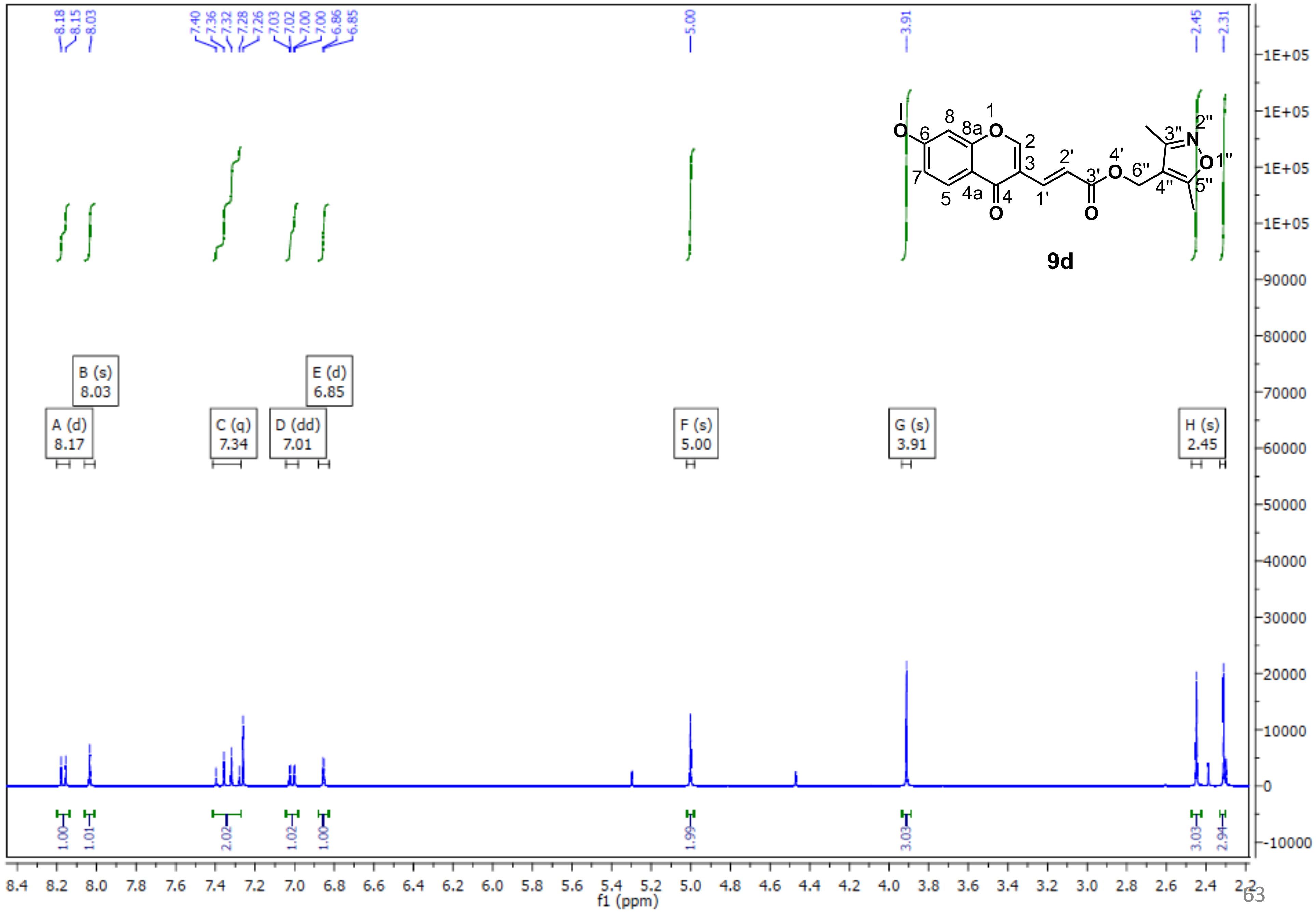

Figure S26.2. ^{13}C NMR spectra of compound 9d

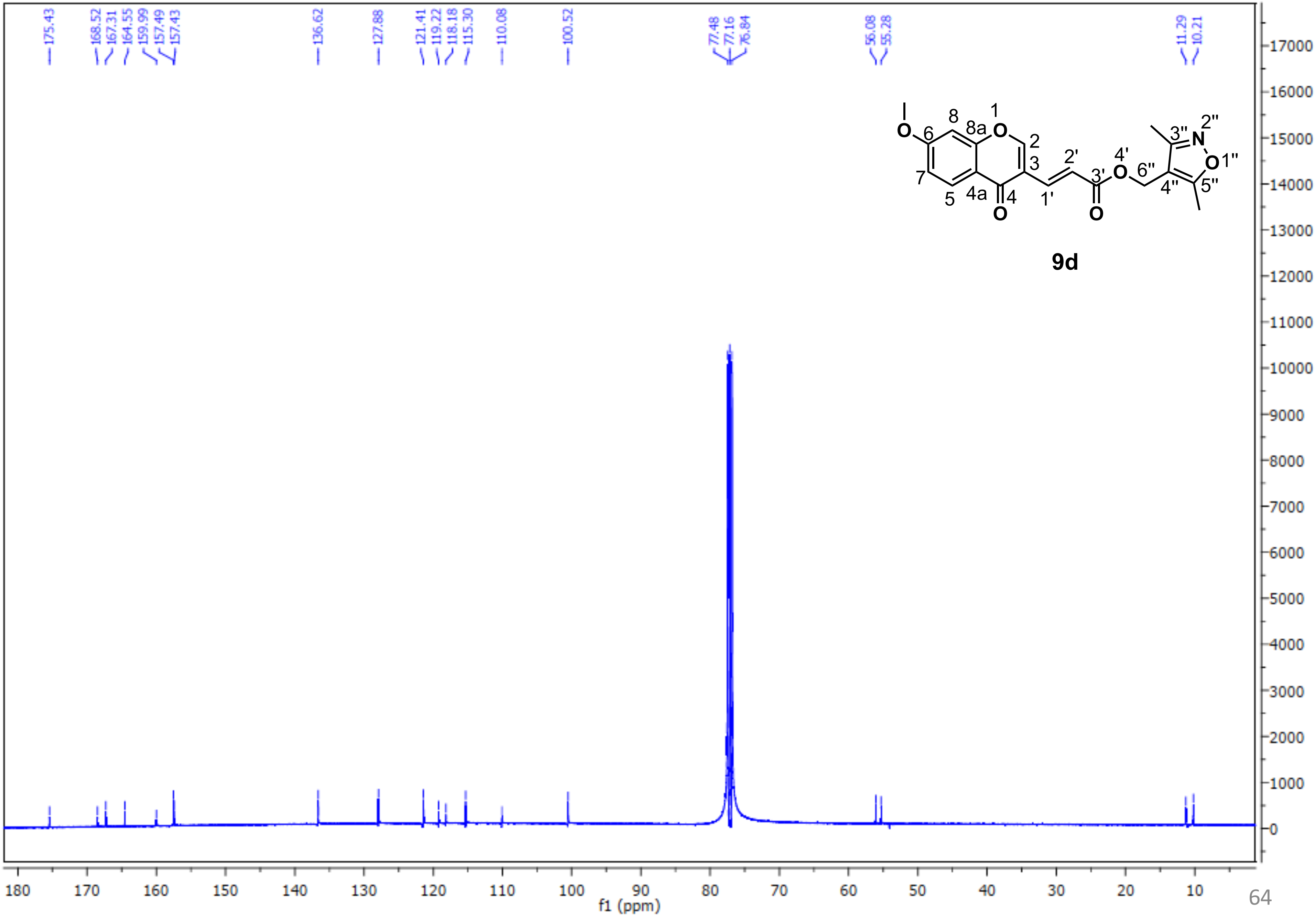

Figure S26.3. ^{13}C-DEPT NMR spectra of compound 9d

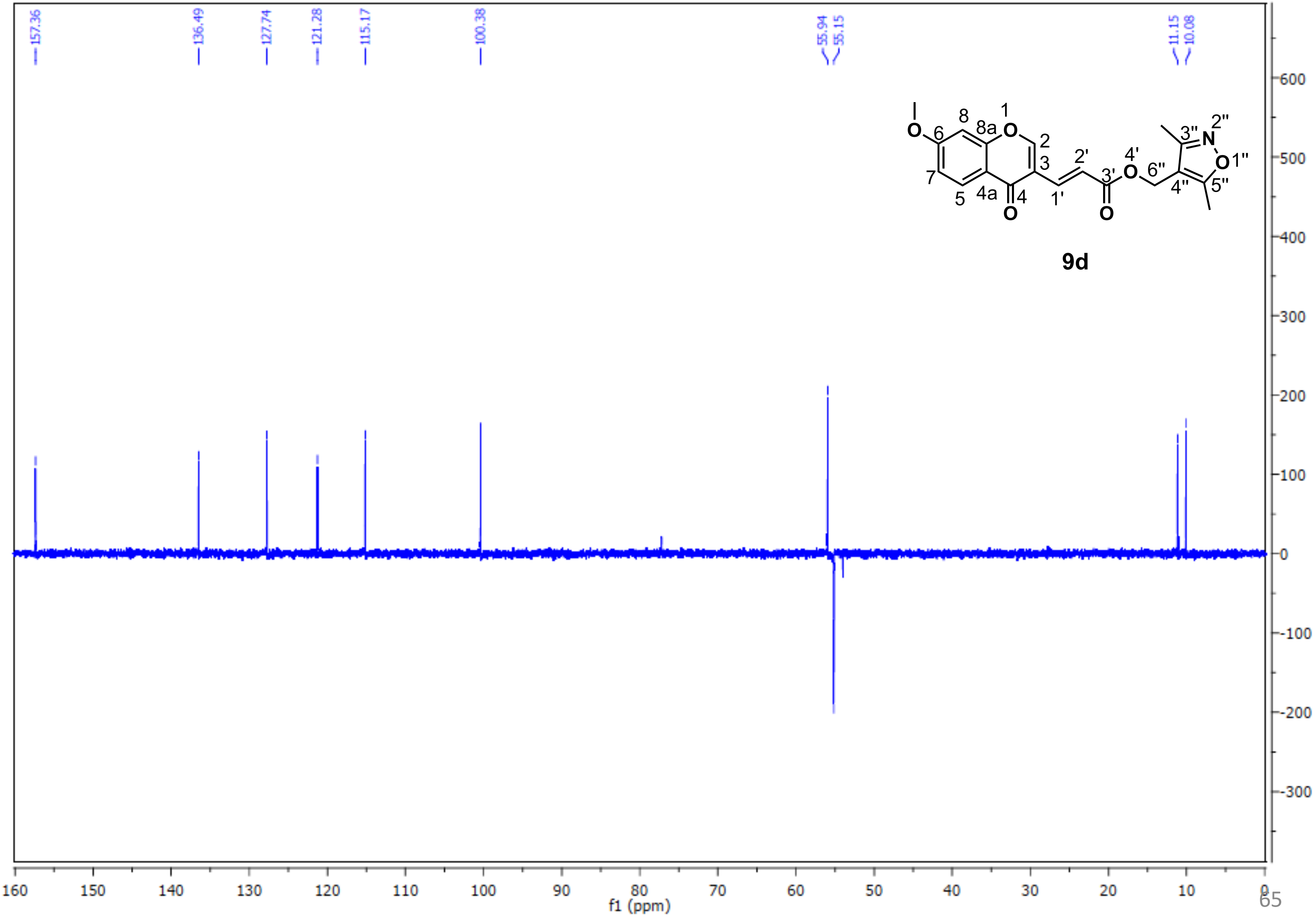

Figure S27.1. Analytical HPLC method 1

Column: C18

Solvent Flow Rate: 1 mL/min

Solvent A: Water + 0.1% TFA

Solvent B: Acetonitrile + 0.1% TFA

Detection wavelength: 254nm

Temperature of the column oven: 30°C

Gradient Method A1:

S. No.	Time	Solvent B (%)
1	0.01	10
2	10.00	10
3	40.00	60
4	42.00	100
5	50.00	100
6	55.00	10
7	60.00	10
8	65.00	STOP

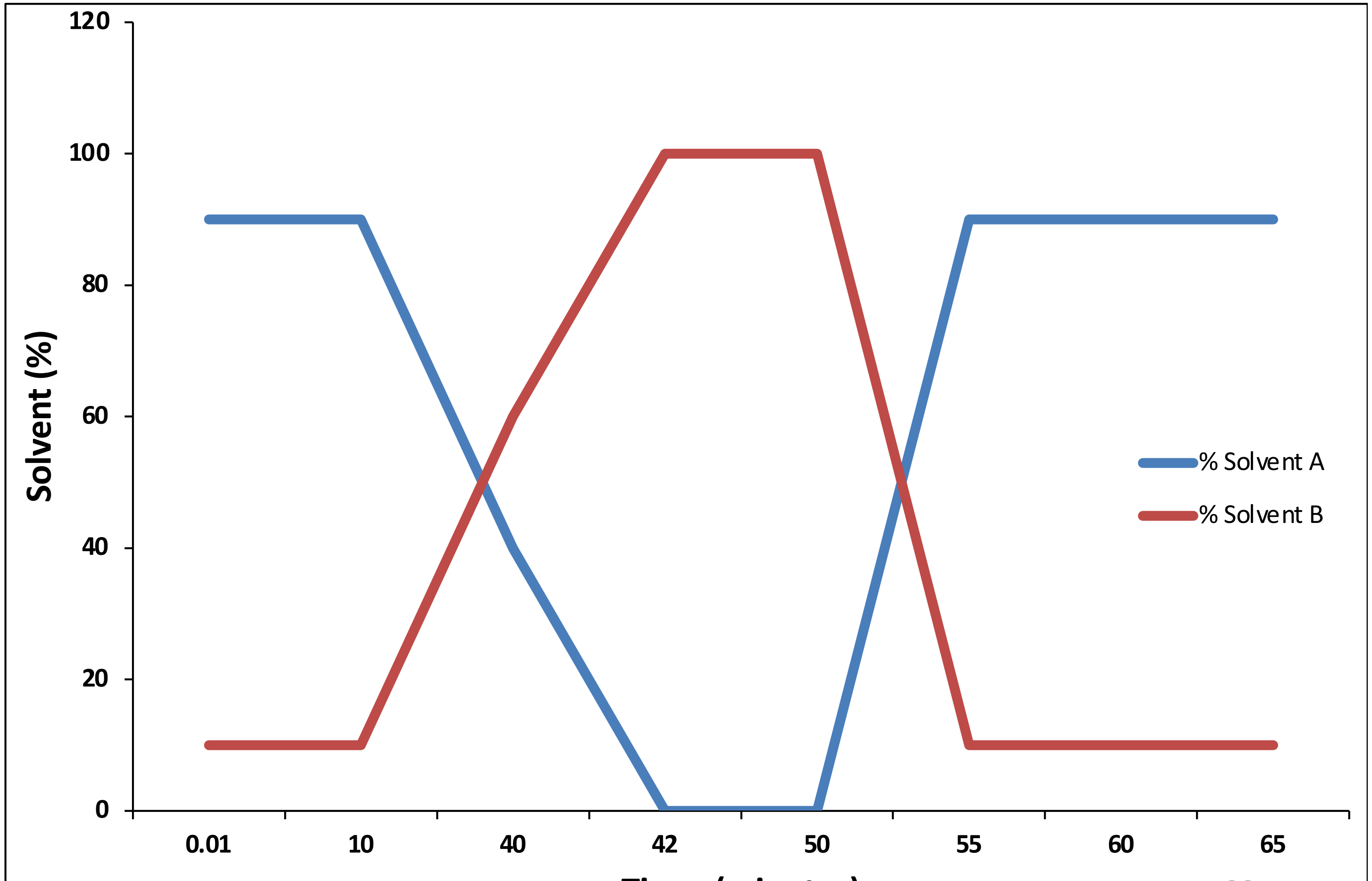

Figure S27.2. Analytical HPLC method 2

Column: C18
Solvent Flow Rate: 1 mL/min
Solvent A: Water + 0.1% TFA
Solvent B: Acetonitrile + 0.1% TFA
Detection wavelength: 254nm
Temperature of the column oven: 30°C
Gradient Method A1:

S. No.	Time	Solvent B (%)
1	0.01	10
2	10.00	10
3	40.00	60
4	42.00	100
5	50.00	100
6	55.00	10
7	60.00	10
8	65.00	STOP

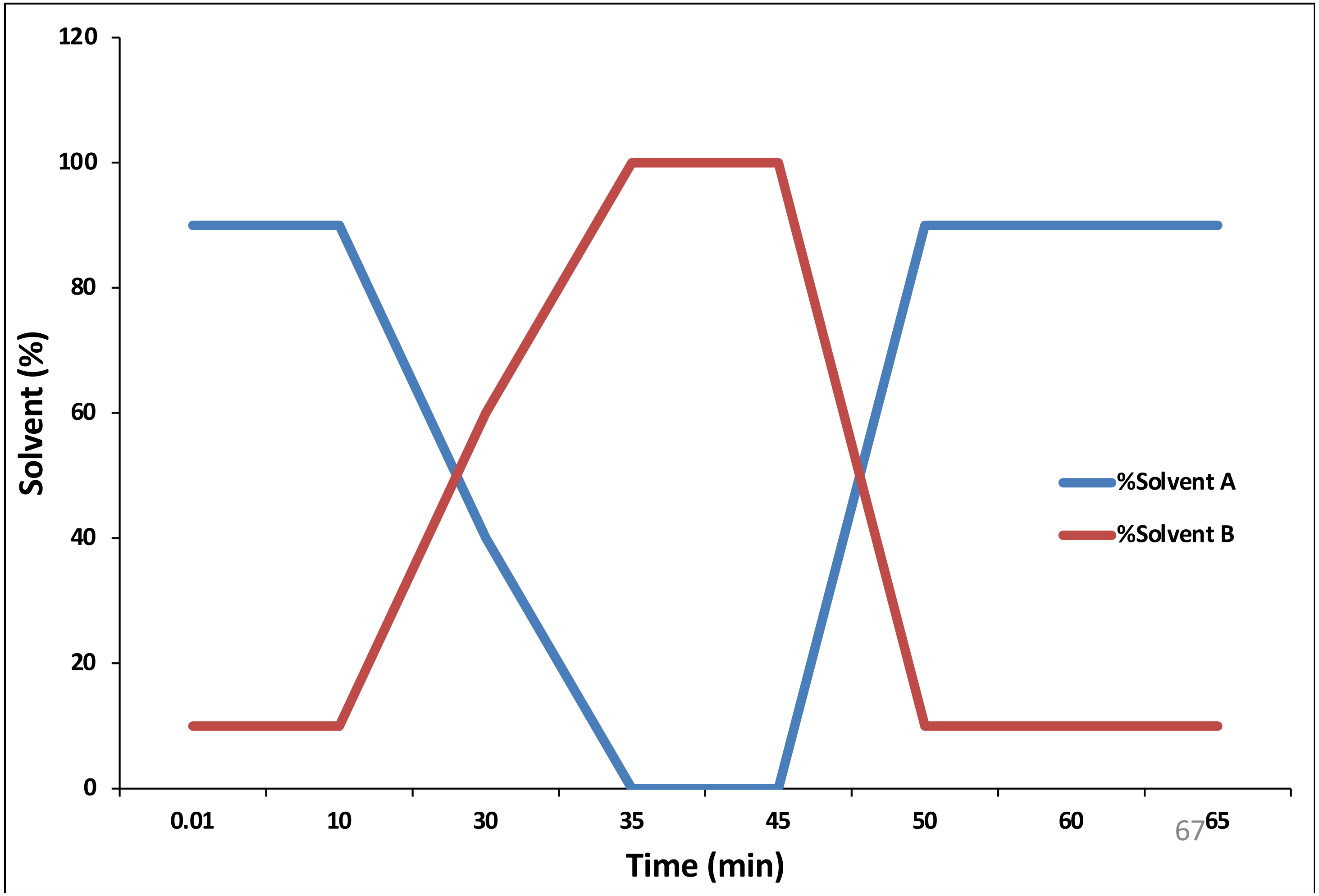

Figure S27.3. Analytical HPLC of representative compounds 5a and 5b

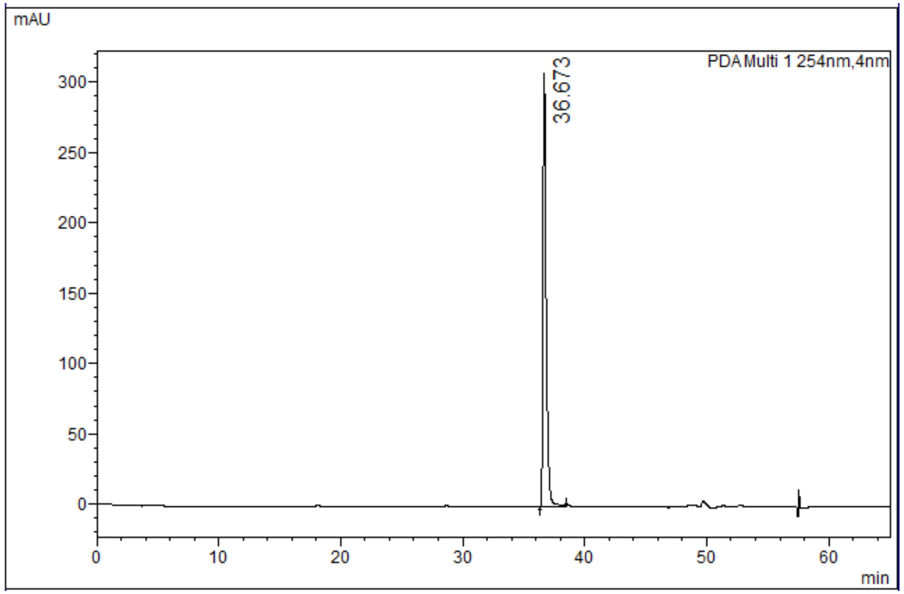

Analytical HPLC of compound 5a by Method 1

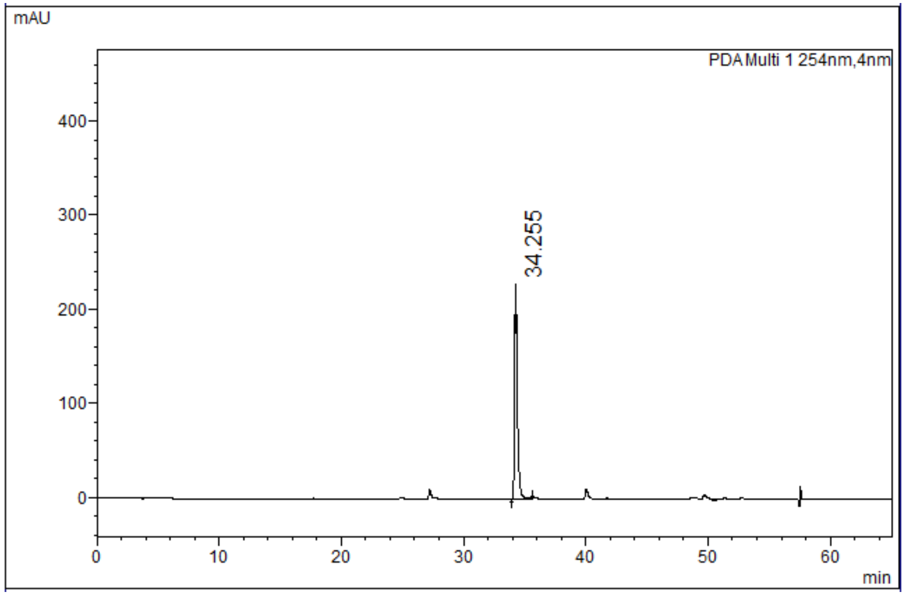

Analytical HPLC of compound 5b by Method 1

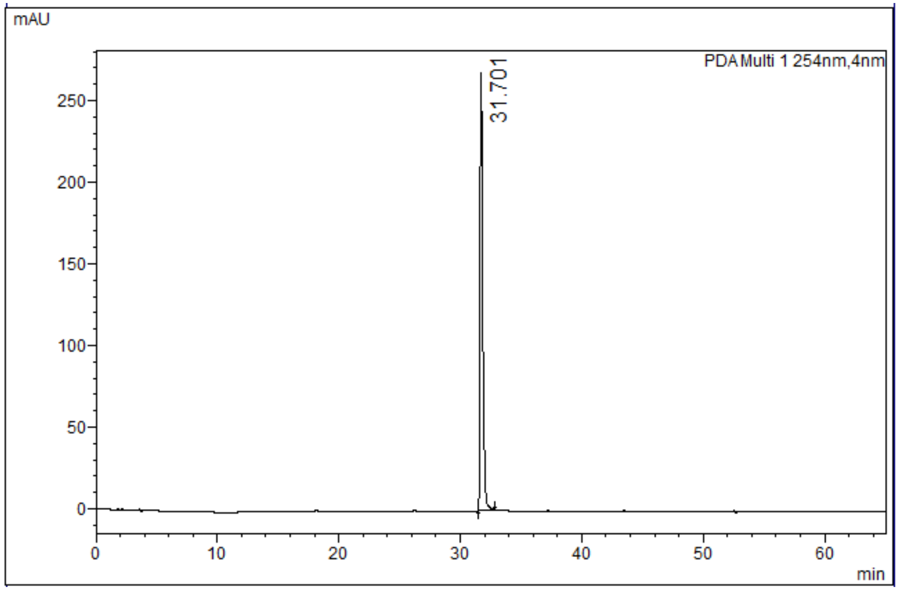

Analytical HPLC of compound 5a by Method 2

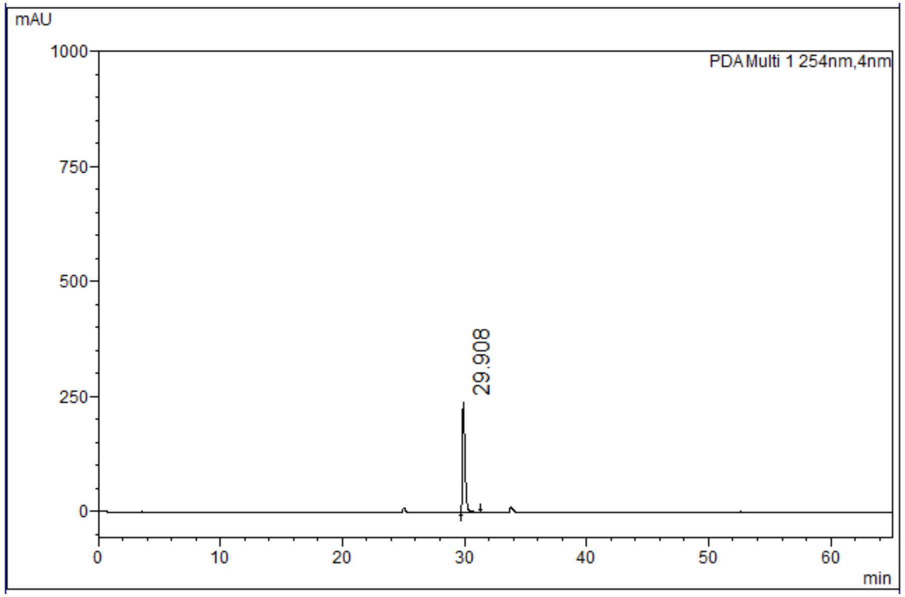

Analytical HPLC of compound 5b by Method 2

Figure S27.4. Analytical HPLC of representative compounds 5c and 5d

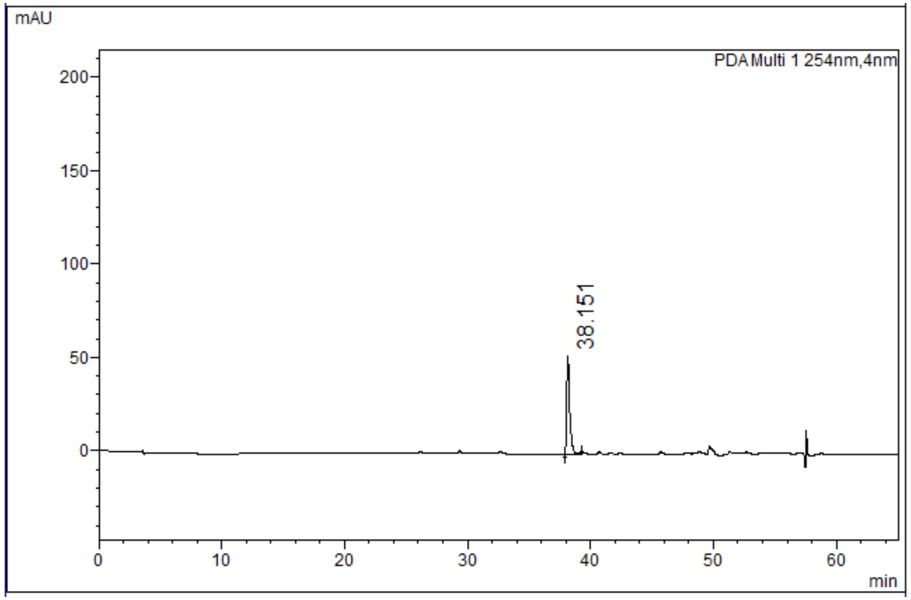

Analytical HPLC of compound 5c by Method 1

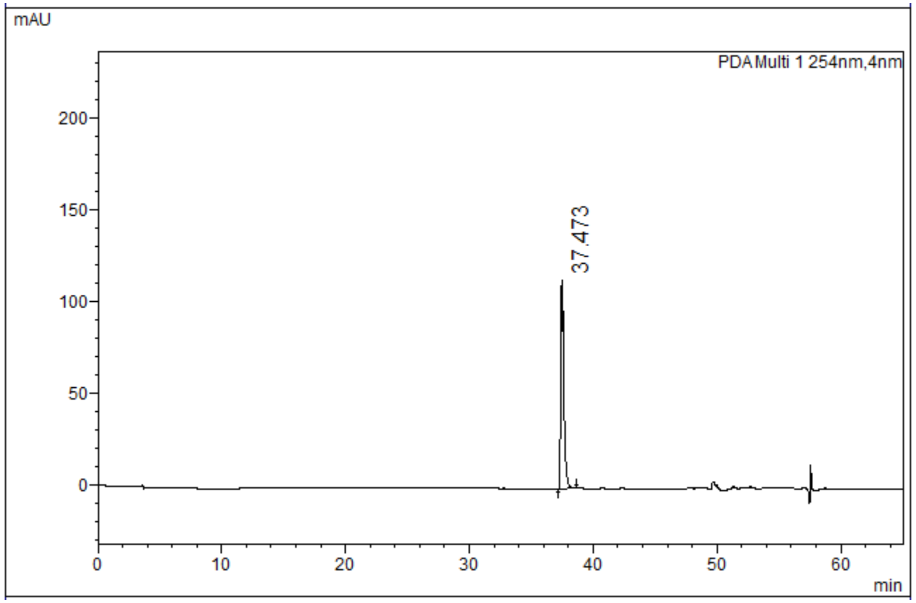

Analytical HPLC of compound 5d by Method 1

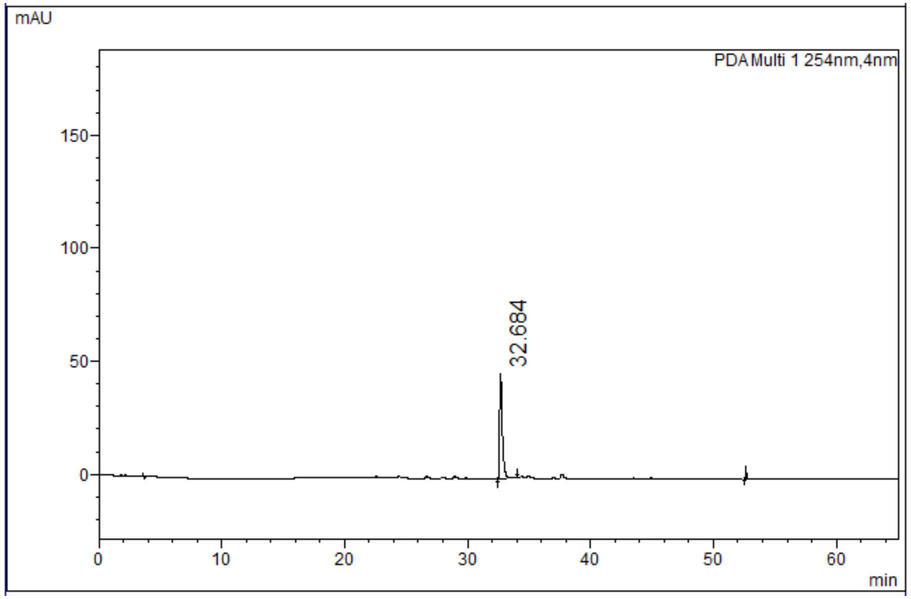

Analytical HPLC of compound 5c by Method 2

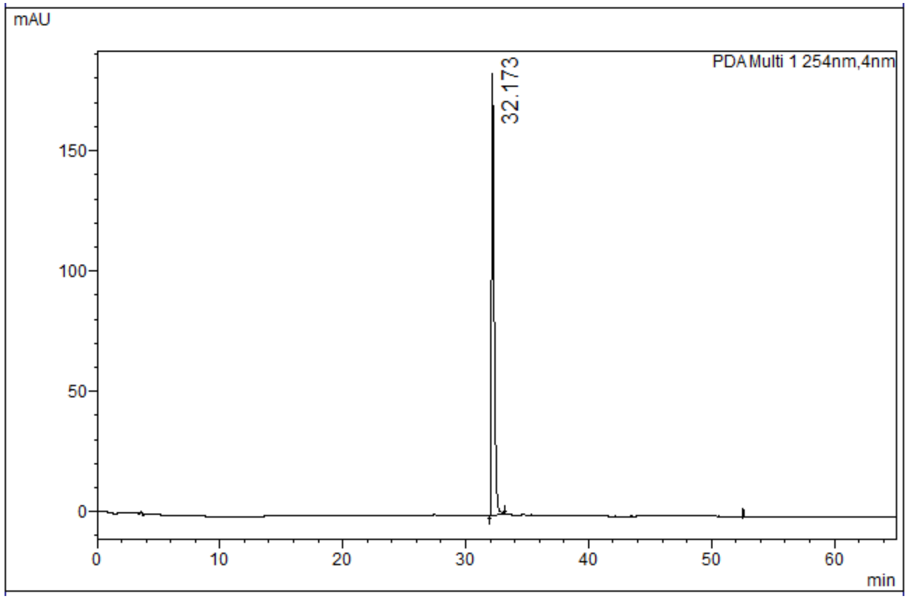

Analytical HPLC of compound 5d by Method 2

Table S1. HPLC retention time of conjugates **5a-5d** using two different methods.

	**Retention time (min)**	
	**Method 1***	**Method 2***
**5a**	36.67	31.70
**5b**	34.25	29.90
**5c**	38.15	32.15
**5d**	37.47	32.17

* Method 1 & 2: Different gradient of solvent system used with Solvent A (water + 0.1% TFA) and Solvent B (acetonitrile + 0.1% TFA) with a flow rate of 1 mL/min (cf. Supporting Information for gradient system and HPLC chromatogram).

Figure S28.1. In-vitro cytotoxicity activity of benzopyran-4-one - isoxazole starting material and intermediates (at a concentration of 25 µM) towards human leukemia carcinoma (CCRF-CEM) cell lines.

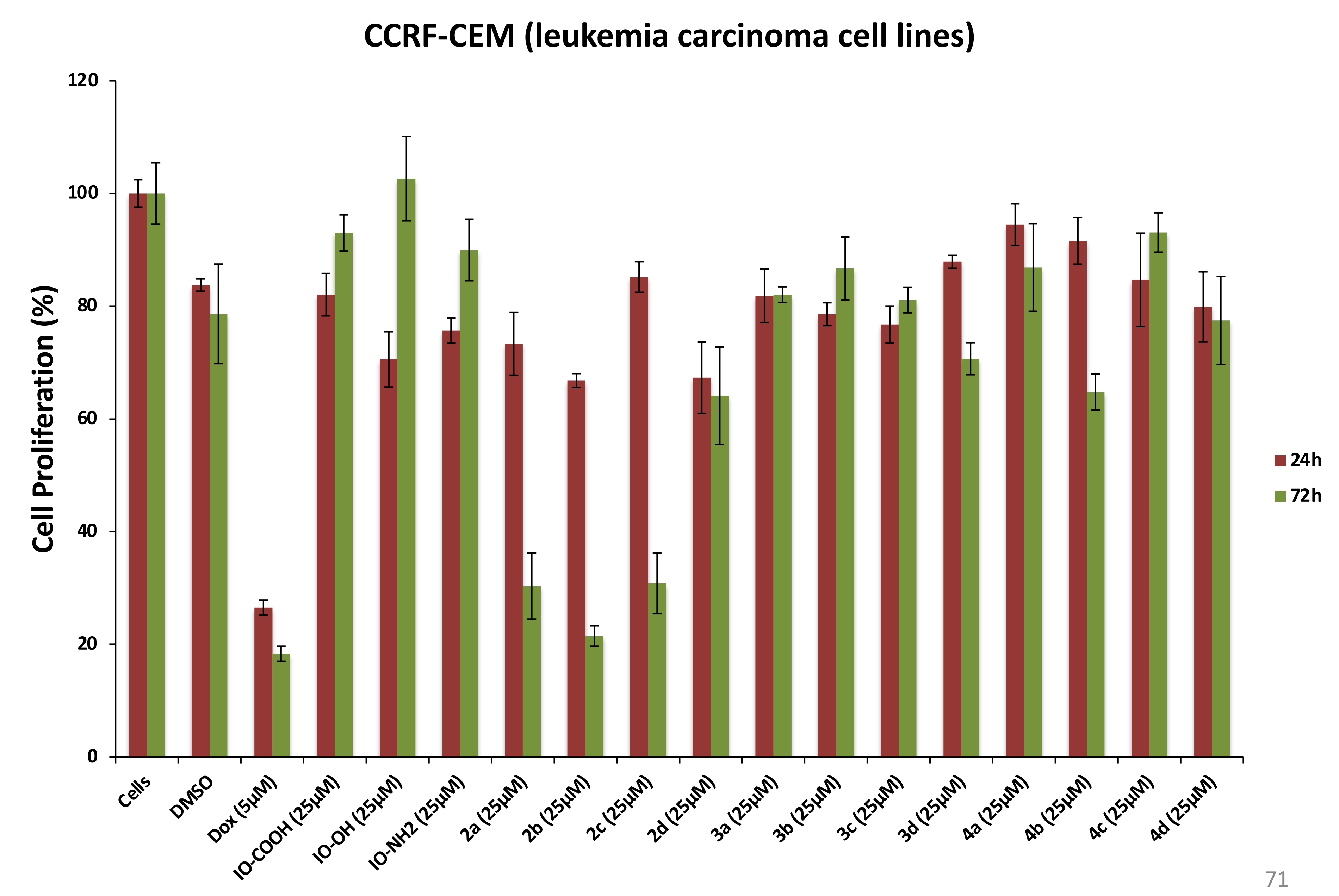

Figure S28.2. In-vitro cytotoxicity activity of benzopyran-4-one - isoxazole starting material and intermediates (at a concentration of 25 µM) towards human ovarian adenocarcinoma (SKOV-3) cell lines.

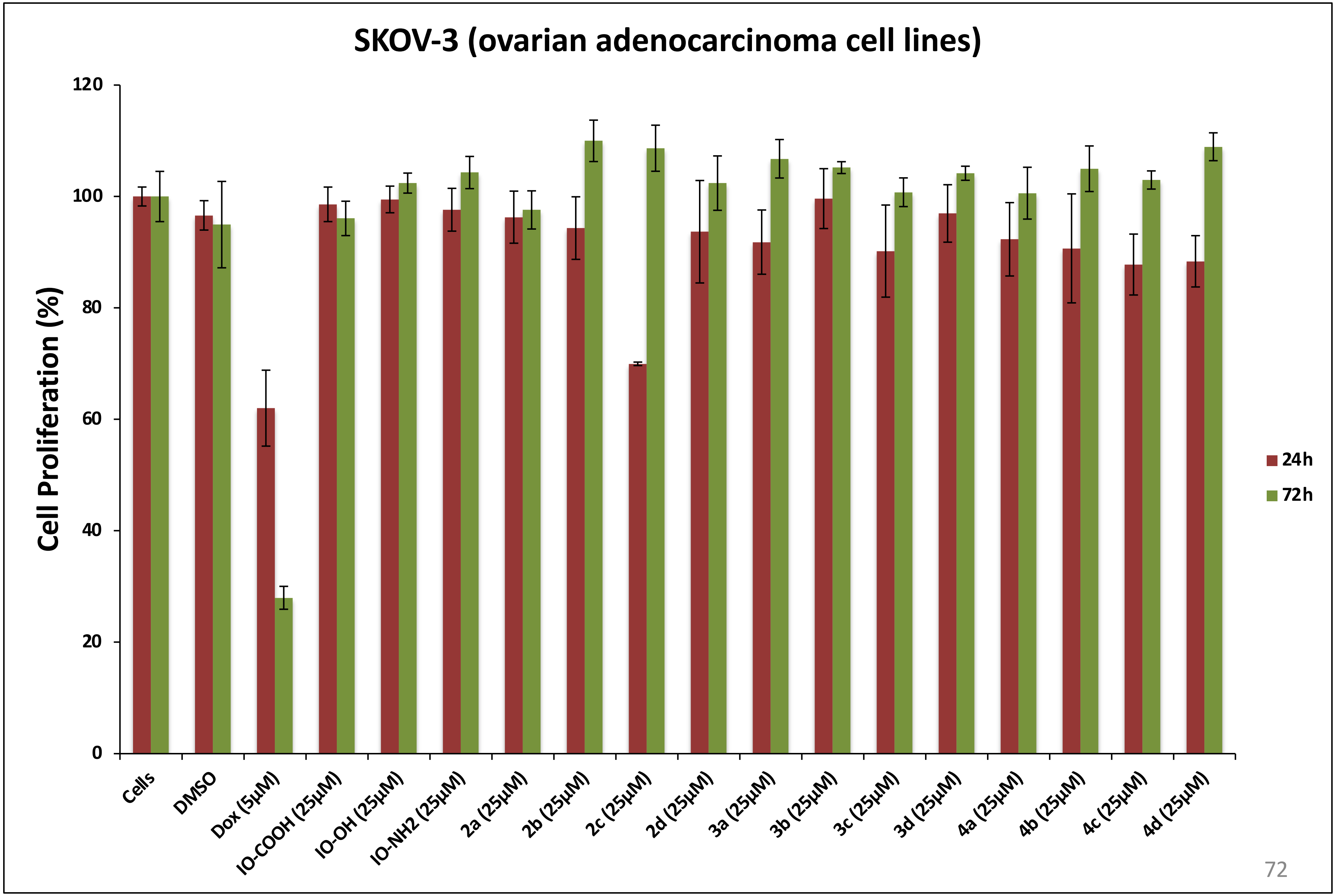

Figure S28.3. In-vitro cytotoxicity activity of benzopyran-4-one - isoxazole starting material and intermediates (at a concentration of 25 µM) towards human breast tumor (MDA-MB-231) cell lines.

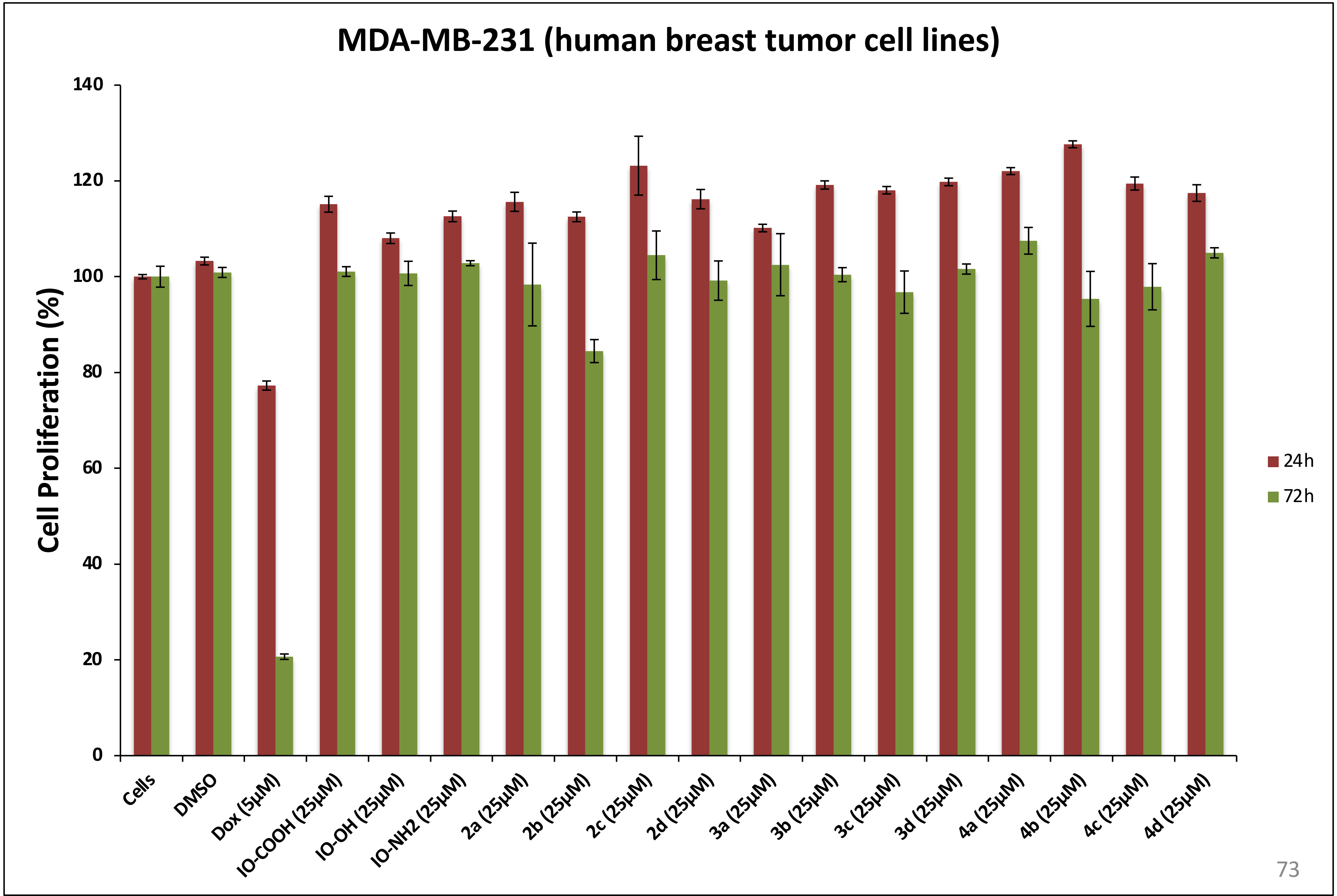

Figure S28.4. In-vitro cytotoxicity activity of benzopyran-4-one - isoxazole starting material and intermediates (at a concentration of 25 µM) towards human prostate cancer (PC-3) cell lines.

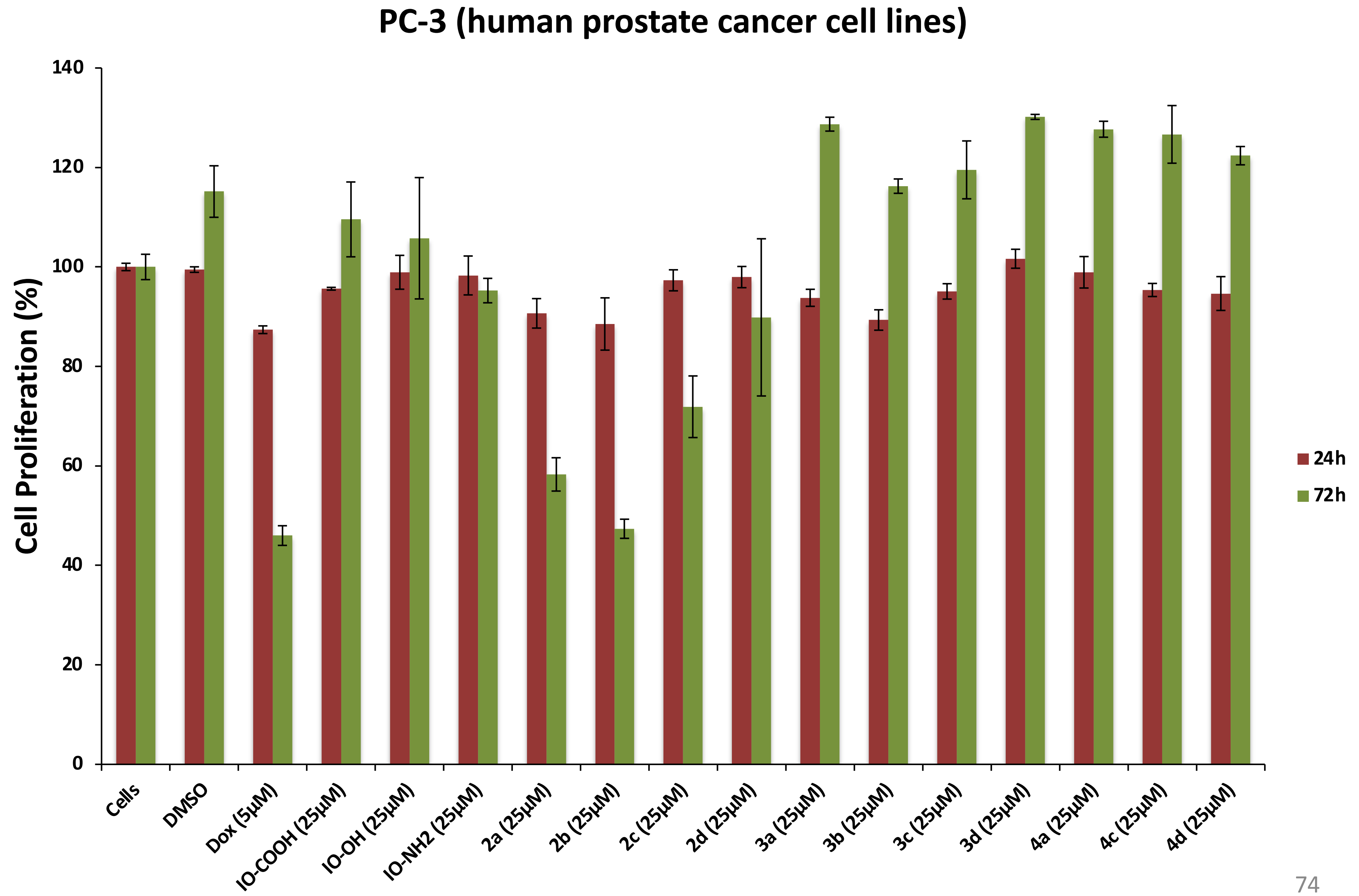

Figure S28.5. In-vitro cytotoxicity activity of benzopyran-4-one - isoxazole starting material and intermediates (at a concentration of 25 µM) towards androgen-independent human prostate cancer (DU-145) cell lines.

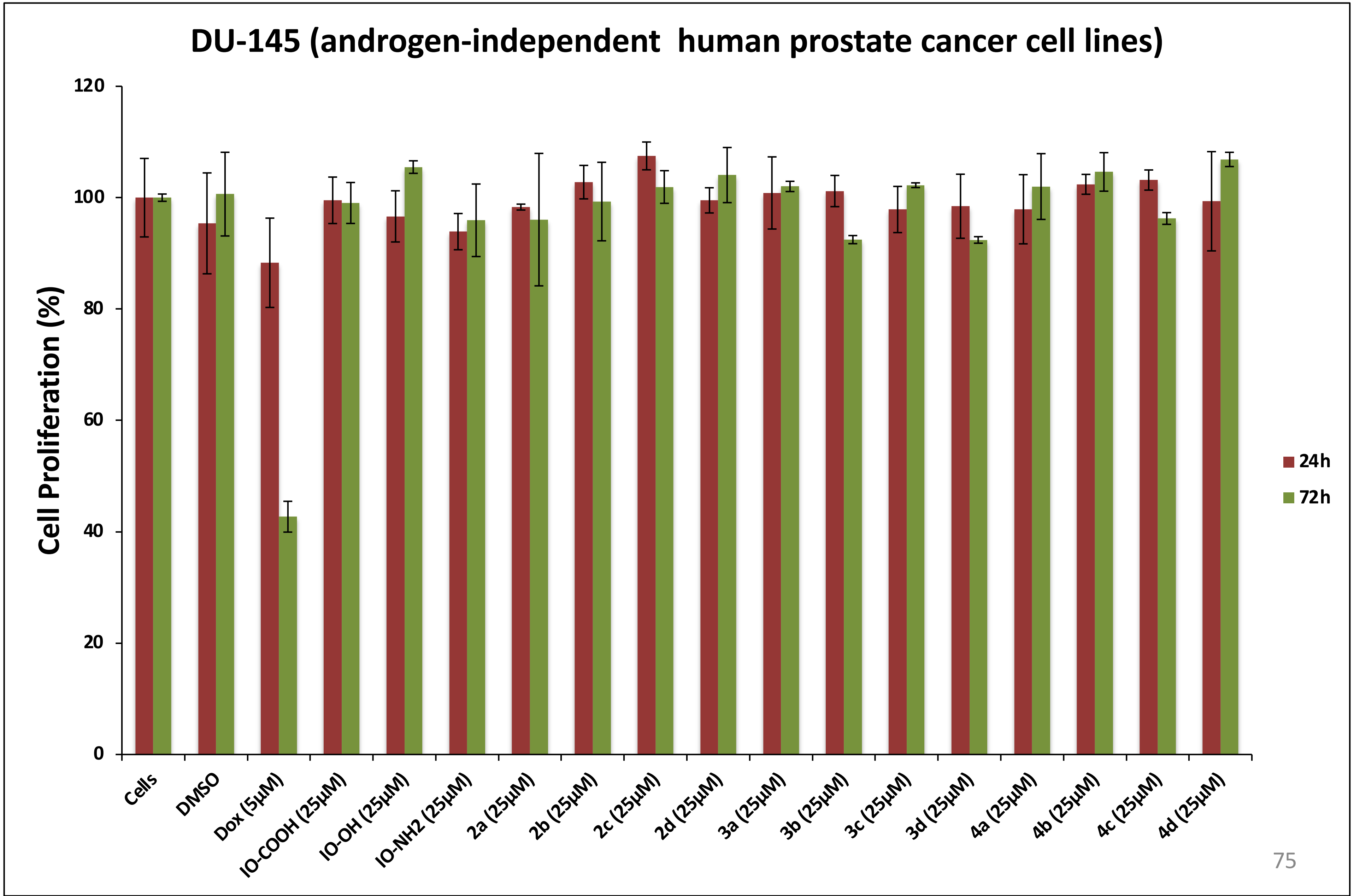

Figure S28.6. In-vitro cytotoxicity activity of benzopyran-4-one - isoxazole starting material and intermediates (at a concentration of 25 µM) towards human renal carcinoma (iSLK) cell lines.

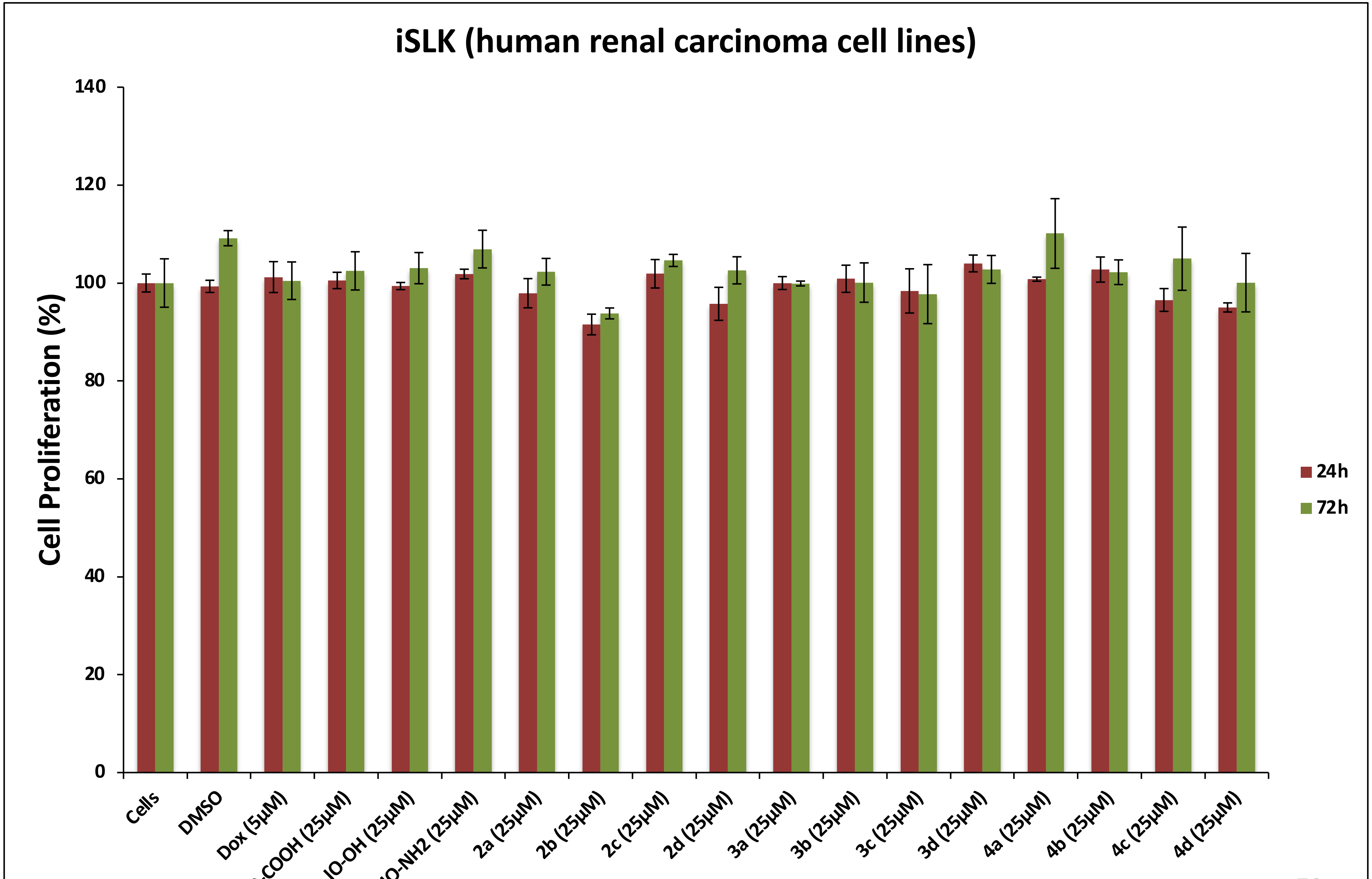

Figure S28.7. In-vitro cytotoxicity activity of benzopyran-4-one - isoxazole starting material and intermediates (at a concentration of 25 µM) towards human embryonic kidney (HEK-293) cell lines.

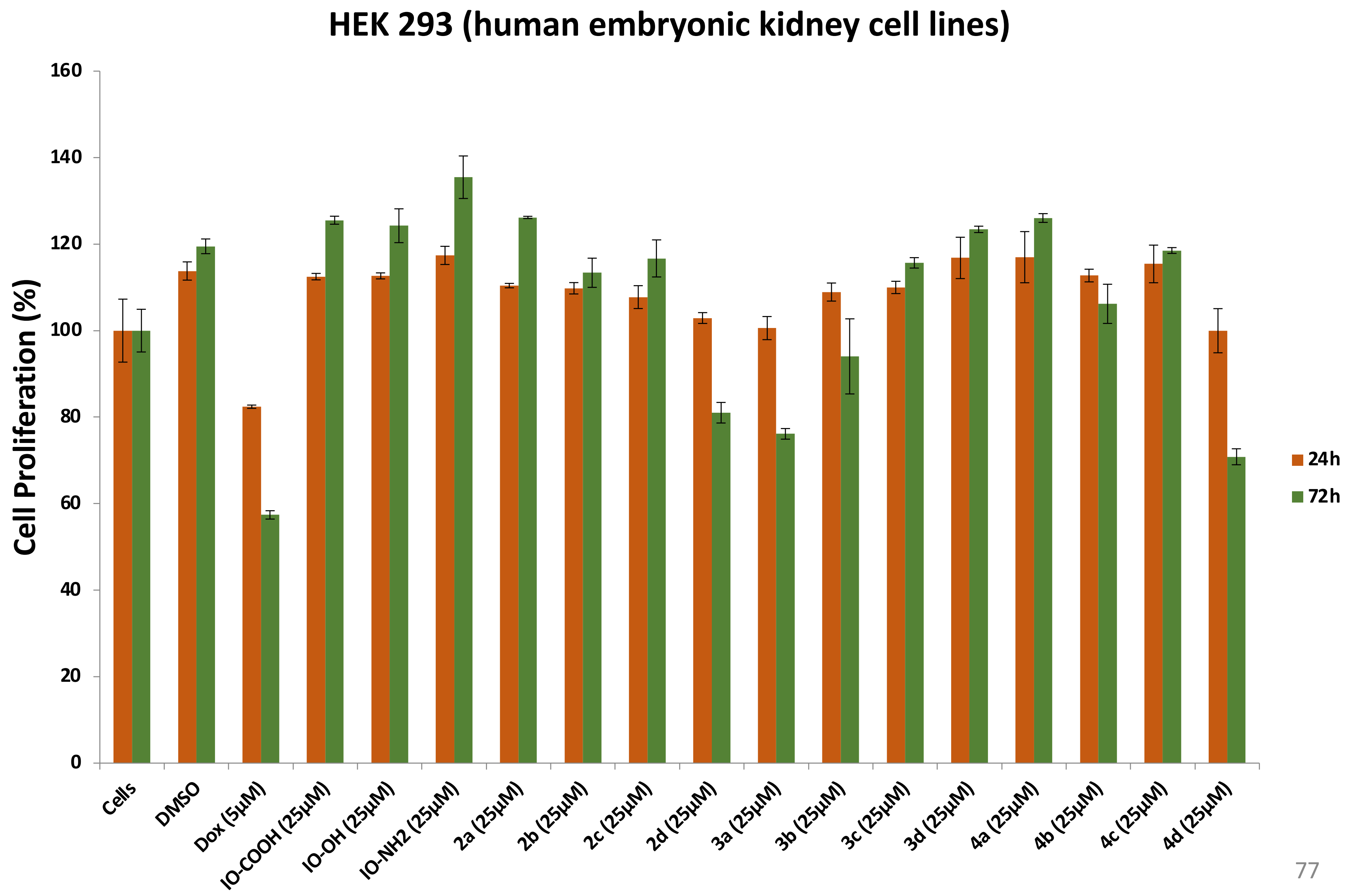

Figure S28.8. In-vitro cytotoxicity activity of benzopyran-4-one - isoxazole starting material and intermediates (at a concentration of 25 μM) towards normal mammalian kidney (LLCPK) cell lines.

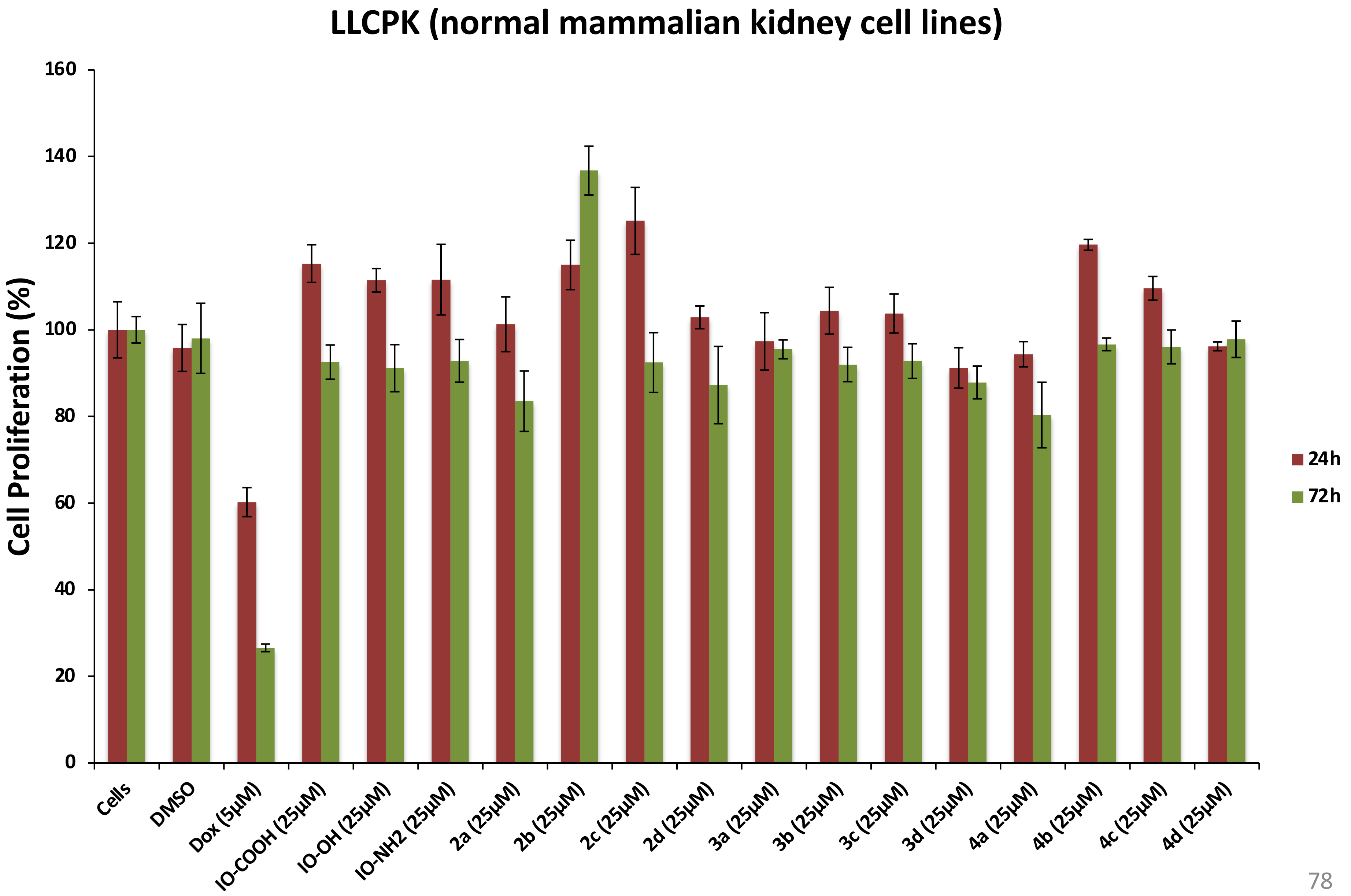

Table S2. Percentage antiproliferative activity on treatment with Dox (at a concentration of 5 µM) and conjugates **5a-5d** (at a concentration of 25 µM) 72 h of incubation.

	Inhibition of Cancer Cell Lines (%, 72 h)					
**Compounds No.**	**CCRF-CEM**	**SKOV-3**	**MDA-MB-231**	**PC-3**	**DU-145**	**iSLK**
**Dox**	82	62	73	54	57	NIL
**5a**	81	69	78	70	73	44
**5b**	83	42	60	75	50	23
**5c**	83	45	70	74	81	31
**5d**	82	48	76	75	79	42

Table S3. Percentage anti-proliferative activity of non-cancerous cell lines on treatment with doxorubicin (at a concentration of 5 µM) and conjugates 5a-5d (at a concentration of 25 µM).

	Inhibition of Non-Cancerous Cell Lines (%, 72 h)	
**Compounds No.**	**HEK 293**	**LLCPK**
**Dox**	43	74
**5a**	37	57
**5b**	34	NIL
**5c**	38	NIL
**5d**	40	18

Figure S29.1 HPLC data comparison for Standard Curve of 5a

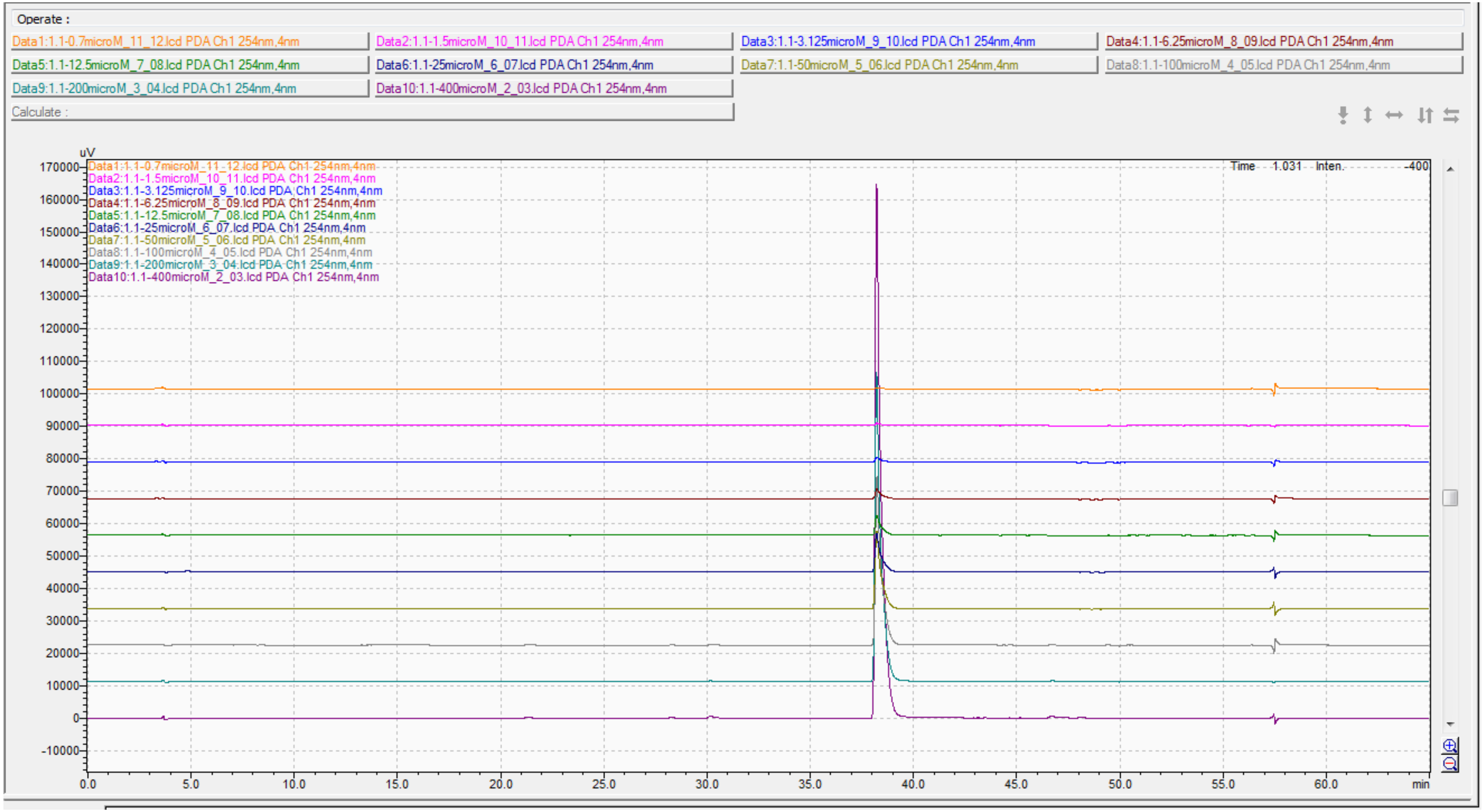

Conc. 5a (μM)	400	200	100	50	25	12.5	6.25	3.12	1.56	0.78
AUC (at 254nm)	3239256	1892892	1034689	482517	236578	122631	59281	28929	14181	4769

Figure S29.2 Plot of area under the curve vs. conc. of 5a

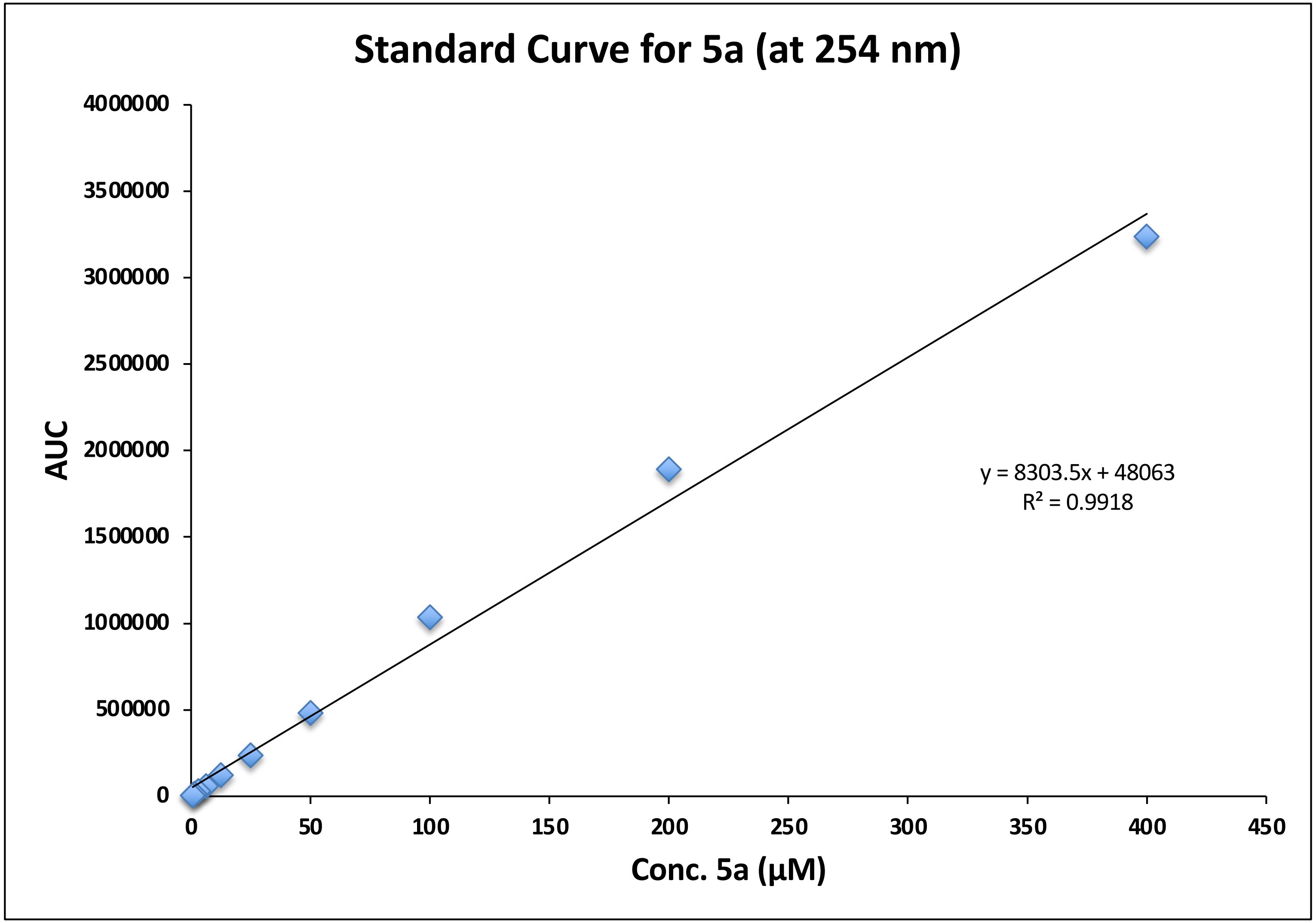

Table S4. Decrease in conc. of 5a on incubation with human serum at different time points (set of 3 independent experiments)

	Conc 5a (µM)					
Time (min)	Expt 1	Expt 2	Expt 3	Average	Std dev	ln (average)
0	76,84	81,37	84,65	100	3,921636563	4,605170186
2	64,28	54,99	50,4	69,86329575	7,071381289	4,246540415
5	52,92	51,81	58,93	67,38861899	3,830722299	4,210476146
10	40,01	43,71	42,37	51,91880098	1,873285883	3,949680978
20	34,49	24,27	34,58	38,4336655	5,926671354	3,648933781
40	18,56	11,93	8,4	16,01334103	5,158219977	2,773422189
80	9,63	0,8	8,3	7,712262212	4,760738738	2,042811557

Calculation of Half life of 5a in Human serum stability

y = -0.031x + 4.3339, where y = ln conc. of 5a and x = time (minutes)

X = (y-4.3339)/-0.031

For $t_{1/2}$

$x_{1/2}$ = (ln 50 – 4.3339)/-0.031

= (3.912-4.3339)/-0.031

= 13.609 min

Figure S30. A plot of human serum stability of **5a** is plotted as a logarithm of conc. of **5a** (μM) vs. time in minutes.

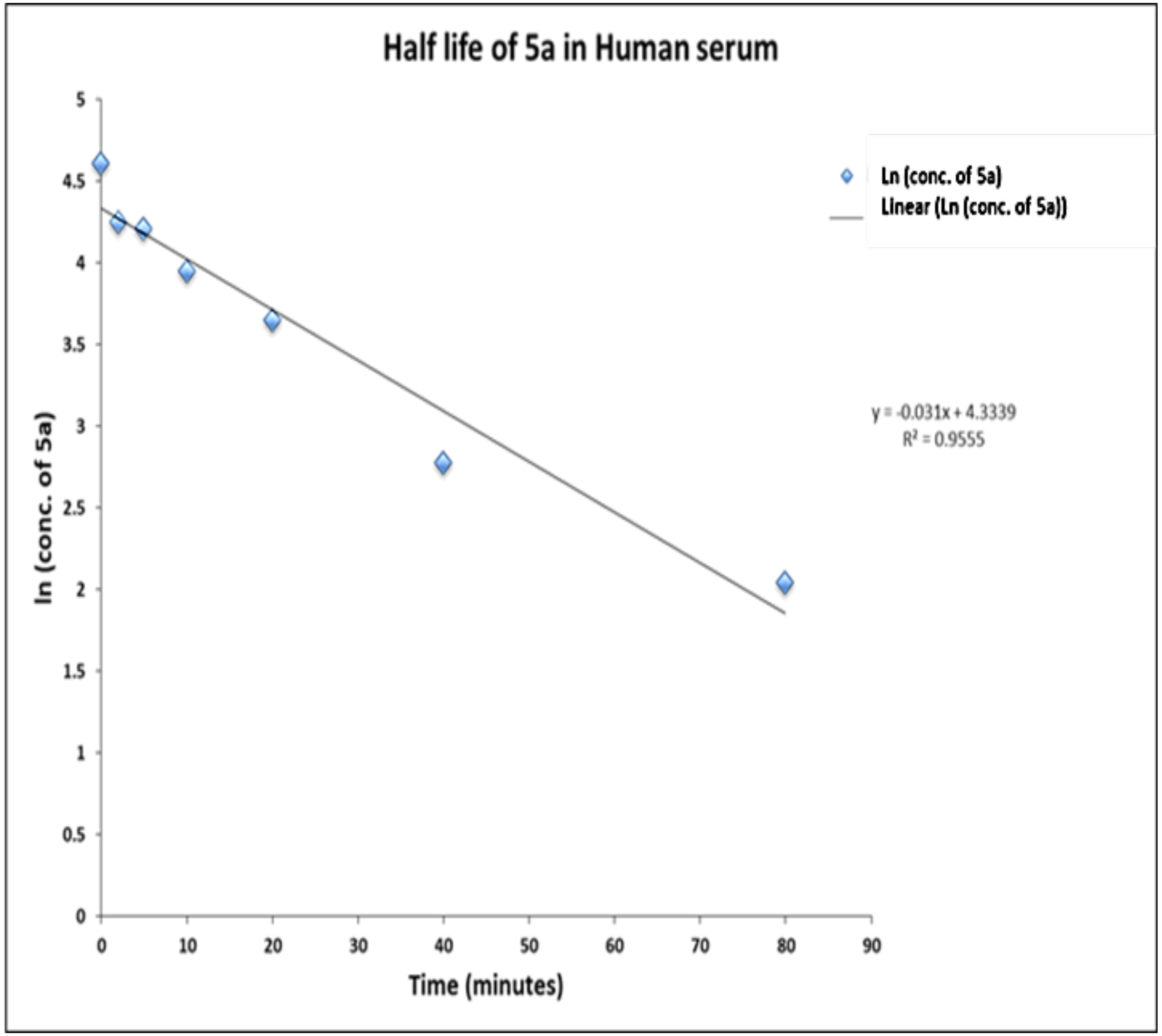

Supplement: Supplementary file 1 [file molecules-28-04220-s001.zip › molecules-2392873-supplementary.pdf]
